# Catalytic Effects of Active Site Conformational Change in the Allosteric Activation of Imidazole Glycerol Phosphate Synthase

**DOI:** 10.1021/acscatal.3c04176

**Published:** 2023-12-06

**Authors:** Heidi Klem, Juan V. Alegre-Requena, Robert S. Paton

**Affiliations:** † Department of Chemistry, Colorado State University, Fort Collins, Colorado 80523, United States; ‡ Dpto.de Química Inorgánica, Instituto de Síntesis Química y Catálisis Homogénea (ISQCH), CSIC, Universidad de Zaragoza, Zaragoza 50009, Spain

**Keywords:** allostery, conformational change, theozyme, quantum chemical cluster approach, oxyanion hole

## Abstract

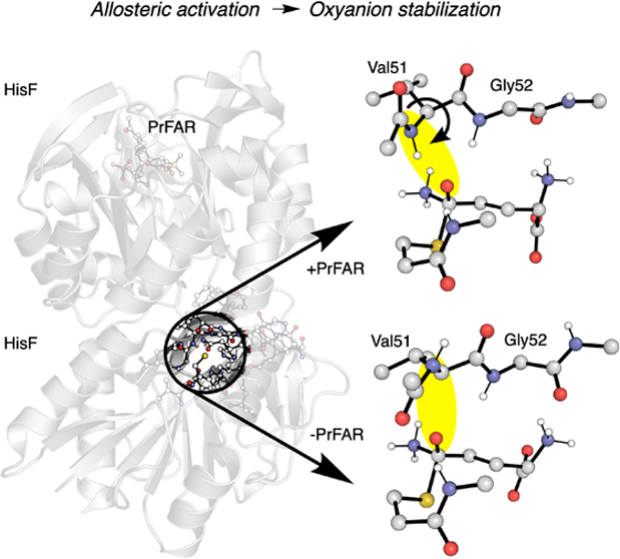

Imidazole glycerol phosphate synthase (IGPS) is a class-I glutamine amidotransferase (GAT) that hydrolyzes glutamine. Ammonia is produced and transferred to a second active site, where it reacts with N^1^-(5′-phosphoribosyl)-formimino-5-aminoimidazole-4-carboxamide ribonucleotide (PrFAR) to form precursors to purine and histidine biosynthesis. Binding of PrFAR over 25 Å away from the active site increases glutaminase efficiency by ∼4500-fold, primarily altering the glutamine turnover number. IGPS has been the focus of many studies on allosteric communication; however, atomic details for how the glutamine hydrolysis rate increases in the presence of PrFAR are lacking. We present a density functional theory study on 237-atom active site cluster models of IGPS based on crystallized structures representing the inactive and allosterically active conformations and investigate the multistep reaction leading to thioester formation and ammonia production. The proposed mechanism is supported by similar, well-studied enzyme mechanisms, and the corresponding energy profile is consistent with steady-state kinetic studies of PrFAR + IGPS. Additional active site models are constructed to examine the relationship between active site structural change and transition-state stabilization via energy decomposition schemes. The results reveal that the inactive IGPS conformation does not provide an adequately formed oxyanion hole structure and that repositioning of the oxyanion strand relative to the substrate is vital for a catalysis-competent oxyanion hole, with or without the *h*Val51 dihedral flip. These findings are valuable for future endeavors in modeling the IGPS allosteric mechanism by providing insight into the atomistic changes required for rate enhancement that can inform suitable reaction coordinates for subsequent investigations.

## Introduction

**Figure 1:**
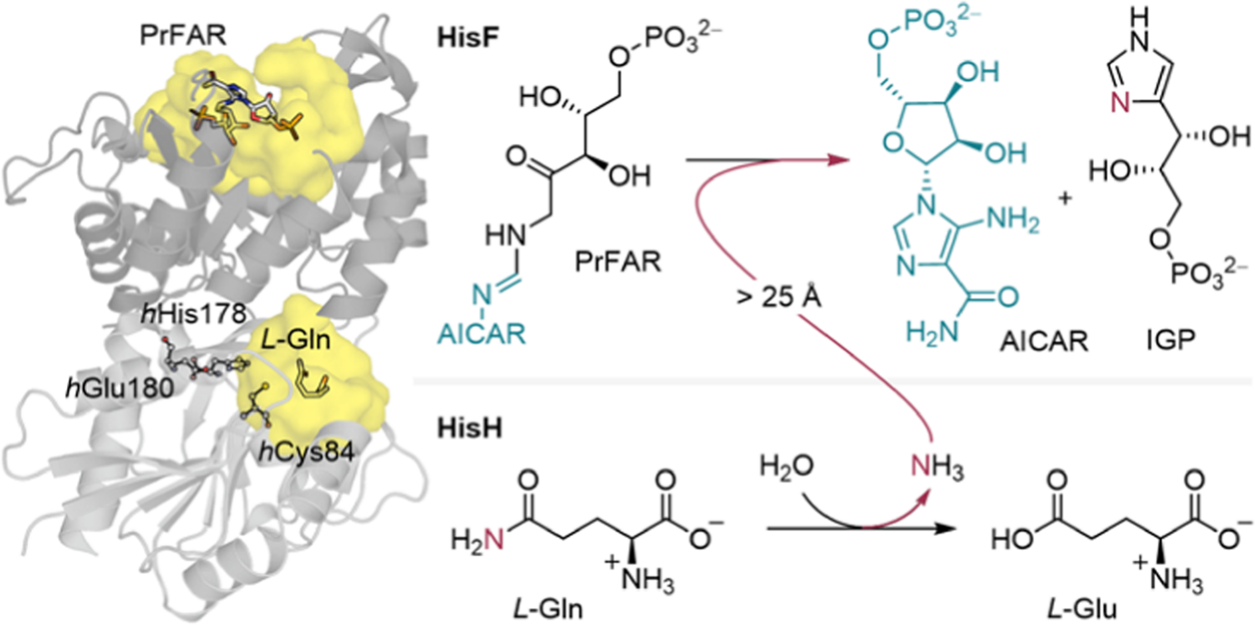
General IGPS scheme of the coupled l-Gln hydrolysis and PrFAR cyclization reactions performed in HisH and HisF, respectively. The l-Gln and PrFAR binding sites are highlighted in yellow.

Imidazole glycerol phosphate synthase (IGPS) is a glutamine amidotransferase (GAT) vital to purine and histidine biosynthetic pathways in bacteria, fungi, and plants, making it an attractive antimicrobial therapeutic target.^1^ IGPS from *Thermotoga maritima* is a heterodimer composed of HisH and HisF subunits (HisFH) (Figure 1).^2^ HisH performs glutamine (l-Gln) hydrolysis to form glutamate (l-Glu) and ammonia using a catalytic triad of *h*Cys84, *h*His178, and *h*Glu180 (*h* and *f* prefixes indicate if the residue belongs to HisH or HisF, respectively), characterizing it as a class-I GAT.^3,4^ The free ammonia is shuttled across the dimer interface to react with N^1^-(5′-phosphoribosyl)-formimino-5-aminoimidazole-4-carboxamide ribonucleotide (PrFAR) in the HisF active site over 25 Å away to form imidazole glycerol phosphate (IGP) and 5′-(5-aminoimidazole-4-carboxamide) ribonucleotide (AICAR).^5^ Binding of PrFAR to HisF elicits a V-type allosteric effect that enhances glutamine hydrolysis efficiency in HisH by approximately 4500-fold, primarily influencing the rate of glutamine turnover.^2,6^

The hydrolysis reaction mechanism has not yet been studied at the atomic level in IGPS, to the best of our knowledge. However, similar well-studied enzymes, such as other class-I GATs and cysteine/serine proteases, provide a useful theoretical foundation.^3,4,7−13^ These reactions are proposed to occur via two stages: acylation and deacylation. In the acylation stage, the nucleophilic *h*Cys84 attacks glutamine to form a glutamyl thioester, first structurally observed in another class-I GAT, carbamoyl phosphate synthetase,^10^ via a tetrahedral oxyanion intermediate. In the deacylation stage, nucleophilic attack by a water molecule breaks down the covalent enzyme–substrate intermediate thioester to yield the glutamate product. The rate-limiting step is proposed to occur during acylation in IGPS, in related class-I GATs, carbamoyl phosphate synthetase, and anthranilate synthase, as well as cysteine esterase.^3,11,14−16^

IGPS serves as a paradigmatic allosteric enzyme for experimental and methodological developments.^17−28^ An abundance of dynamical information contributes to our understanding of the allosteric mechanism in IGPS at the molecular level. A sequence of residues (*h*Pro49—*h*Gly50—*h*Val51—*h*Gly52 in IGPS) comprise the oxyanion strand, a structural motif common to class-I GATs that positions a backbone amide hydrogen to stabilize the formation of an oxyanion throughout the reaction.^29−33^ Increased flexibility in the IGPS oxyanion strand upon PrFAR binding was observed in NMR experiments and molecular dynamics (MD) simulations, supporting its mechanistic involvement.^26,34,35^ An interfacial hydrogen bond between *f*Pro10 and the backbone N–H of *h*Val51 is weakened in MD simulations with PrFAR,^27,36^ and explains the enhanced flexibility of the oxyanion strand observed in NMR experiments.^26,35^ PrFAR also reduces the opening angle of the interface, which is expected to influence the hydrolysis reaction, although it is unclear if there is a direct effect on the reaction mechanism.^6,20,34,37^

A leading hypothesis to explain the allosteric rate effect in IGPS is that PrFAR enables a conformational change in the *h*Val51 backbone that lowers the rate of glutamine hydrolysis via a catalytically competent oxyanion hole.^14,36,38,39^ The *h*Val51 amide C=O points into the active site and the N–H away in all but one crystallographic conformation of IGPS in various ligand bound states.^5,6,40−42^ The anomalous conformation is observed in a recently deposited structure 7ac8, chains E and F with bound allosteric ligand and the Gln substrate.^14^ The catalytic *h*Cys84 was mutated to alanine in this structure to disable glutamine turnover and capture IGPS in the presumably active conformation. Osuna and co-workers provide additional support for this hypothesis through MD simulations employing a biasing potential to sample the *h*Val51 dihedral flip transition. The energetic barrier of this process was estimated to be lower in simulations with PrFAR present (approximately 8 kcal/mol) compared to without PrFAR present (approximately 22 kcal/mol).^36^

Exploration of an alternative activation mechanism is warranted for a few reasons. Extensive (10 μs) unbiased MD simulations of IGPS only reproduced the *h*Val51 dihedral transition when neither Gln nor PrFAR were present, contrary to what was expected.^25^ The authors noted that the novel IGPS conformation revealed in the 7ac8 crystallographic model could be an artifact of the loss of function *h*Cys84Ala mutation rather than intrinsic to the allosteric mechanism. An alternative activation mechanism that has yet to be evaluated in IGPS involves repositioning of the oxyanion strand relative to the Gln substrate. This activation hypothesis has been proposed for another class-I GAT, aminodeoxychorismate synthase (ADCS), since the presence of two prolines in its oxyanion strand (Pro51—Gly52—Pro53) inhibits Gly52 backbone rotation.^43^ All Gln bound IGPS structures show a hydrogen bond between the *h*Gly52 N–H and Gln carbonyl; however, Gln is presumed to be too far from *h*Cys84 to facilitate the nucleophilic attack. The increased oxyanion strand flexibility observed in the presence of PrFAR could facilitate the oxyanion strand reorganization necessary to stabilize the substrate after the nucleophilic attack.

Despite various X-ray structures, mutagenesis studies, kinetic experiments, and MD simulations, a connection between the allosteric effect and the glutamine hydrolysis mechanism remains hypothetical. This work targets two essential questions regarding IGPS activation. How do local structural aspects of the active site influence the reaction energetics? Is the *h*Val51 backbone flip required for rate enhancement? To address these questions, we present a dispersion-corrected density functional theory (DFT) study on large (237 atoms) active site cluster models of IGPS in various active and inactive conformations. The multistep reaction leading to thioester formation and ammonia production is investigated, and energy decomposition analyses are performed to evaluate the relationship between active site geometry and reaction stabilization. Our results are valuable for future endeavors in modeling the allosteric behavior of this prototypical system by providing clear insights into the atomistic changes required for rate enhancement.

Although rigorous workflows have been devised to consider active site multistate effects on catalysis,^44−47^ this work affords a simplified approach, given the available crystallographic data and scope of knowledge from preceding investigations. In doing so, our application of the quantum chemical cluster approach^48^ to investigate an allosteric effect and compare the catalytic impact of active site conformational change traverses a challenge in the field, as recently noted by Himo and de Visser.^49,50^

## Results and Discussion

### Active Site Models

**Figure 2:**
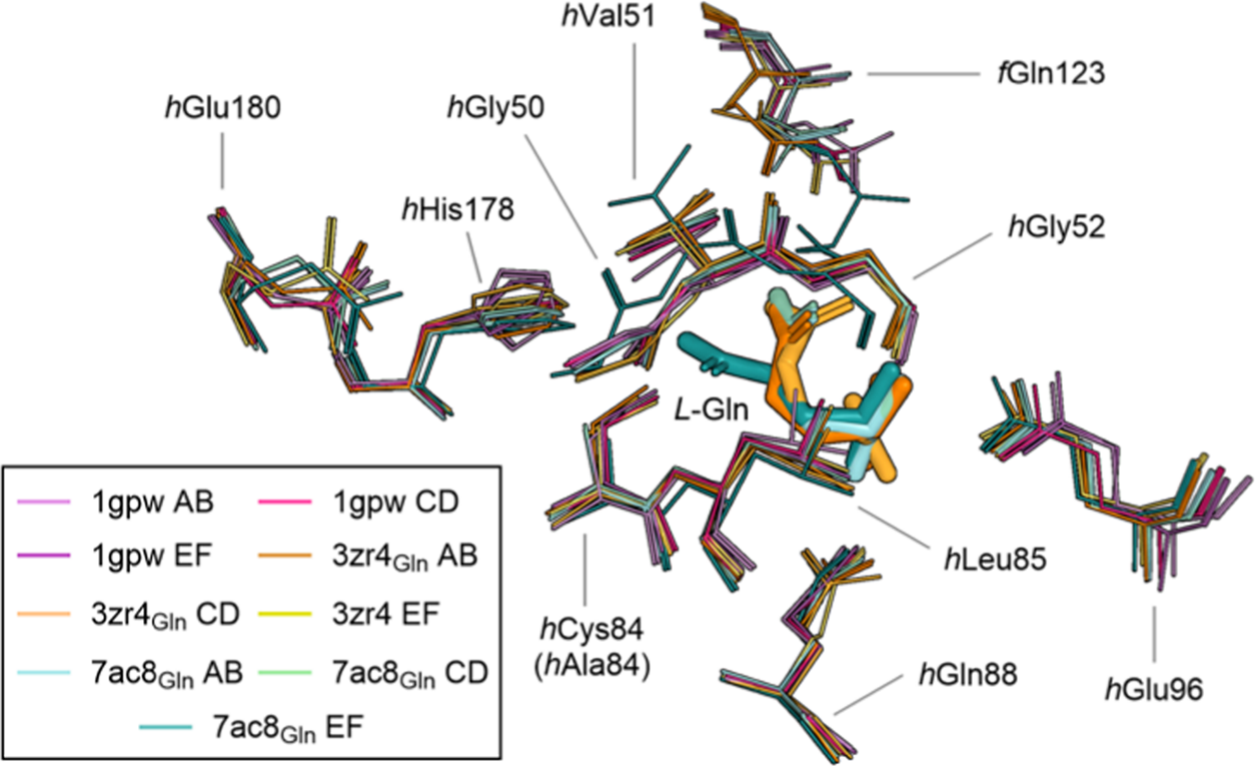
Overlap of HisH active site geometries across multiple crystallized structures.

Positions of the active site residues and the Gln substrate are highly conserved in crystallographic models except for the HisFH dimer conformation composed of chains E and F from PDB entry 7ac8 (Figure 2). There are four distinct geometric differences observed in this conformation: (1) an interfacial residue, *f*Gln123, interacts with the bound Gln as a result of subunit closure; (2) the *h*Val51 amide N–H points toward the substrate carbonyl; (3) the oxyanion strand is positioned above the catalytic thiol; and (4) the reactive carboxamide of Gln is preorganized for acylation.

Two truncated active site models were created from the 7ac8^14^ crystallized unit of IGPS using atomic positions from chains E/F and chains C/D; the deposition authors refer to these complexes as active and inactive conformations, respectively. The residue *h*Ala84 in chains F and D was reverted back to wild-type *h*Cys84 with PyMol^51^ by selecting the backbone-dependent side-chain rotamer with the least steric clash. We refer to these truncated models as Active and Inactive.

**Figure 3:**
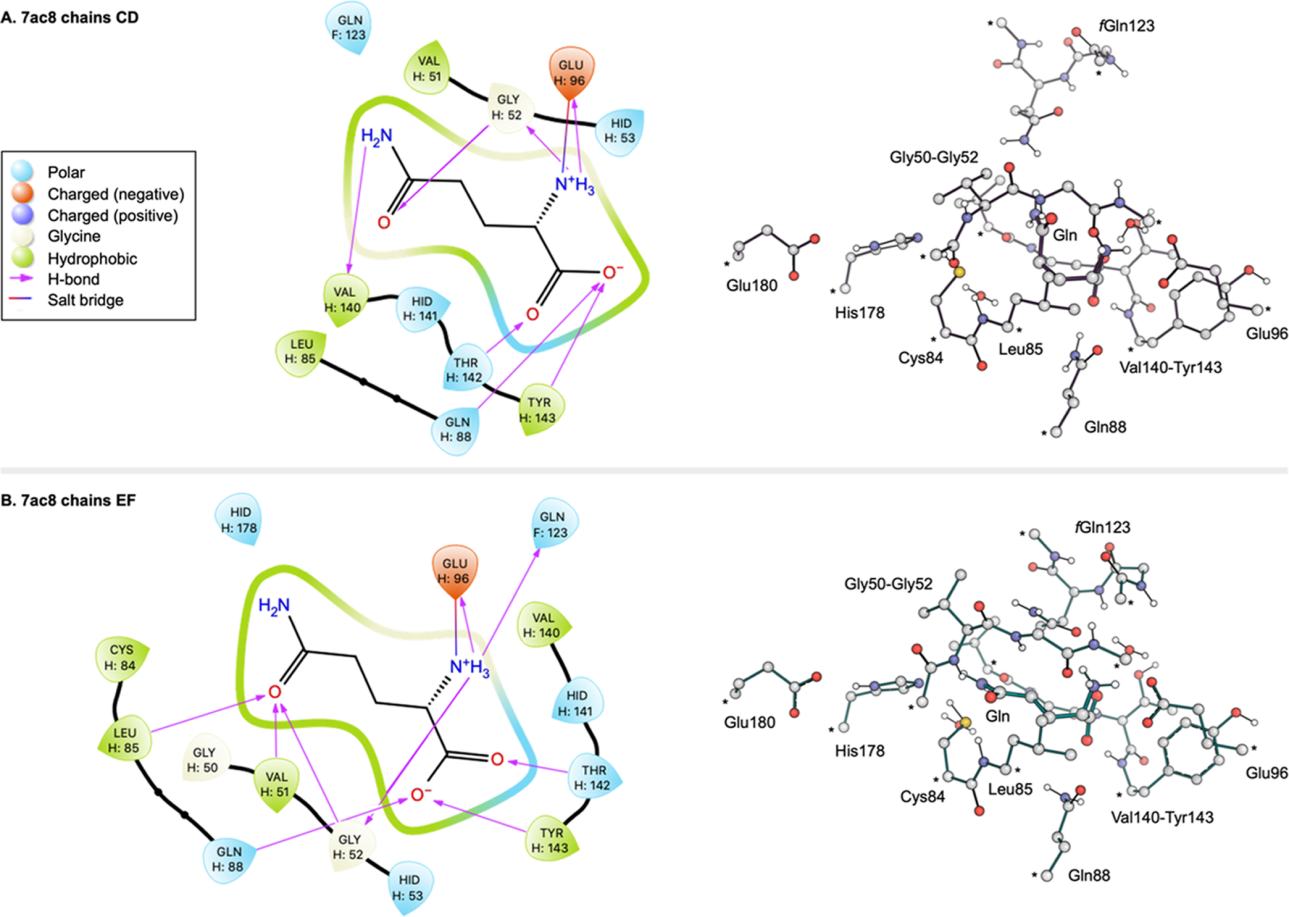
Interactions at the HisH active site and resulting QM cluster models of the (A) inactive and (B) active conformations. * indicate Cα atoms frozen during geometry optimizations. Additional details of the active site model components are provided in Table S1.

The size and residue components of the QM model are important to consider, and informed decisions based on selection criteria remain an active area of development.^52−57^ Residues were selected based on interactions (Figure 3) with the substrate and biochemical relevance indicated in the literature. The ligand interaction diagrams in Figure 3 illustrate how the C/D and E/F conformations yield different interactions with the glutamine substrate. Since the main objective of this work is to evaluate the catalytic impact of active site structural changes, we focused on building models suitable for direct comparison. Therefore, the same atoms were included in each model from the following residues: *f*Gly121, *f*Ser122, *f*Gln123, and *f*Ala124 from the HisF subunit, *h*Gly50, *h*Val51, *h*Gly52, *h*His53, *h*Cys84, *h*Leu85, *h*Gln88, *h*Glu96, *h*Val140, *h*His141, *h*Thr142, *h*Tyr143, *h*His178, and *h*Glu180 from the HisH subunit, the Gln substrate in zwitterionic form, and two conserved crystallographic waters. Hereon, all residues without a prefix are assumed to be from the HisH monomer since they are the model majority, but the HisF prefix will be kept for clarity. Amino acid side and main chains were truncated according to potential involvement in the elementary reaction steps and interactions with the substrate (see Table S1 and Supporting Information Text for a detailed list of included atoms, truncation scheme, and geometry optimization constraints).

Each model contains 237 atoms, an overall charge of −2, and 12 constrained carbon atoms (Figure 3). All energies are obtained at the B3LYP-D3(BJ)/6-311+G(2d,2p) (IEFCPCM, solvent = diethyl ether)//ωB97X-D/6-31G*(C,H,N);6-31+G*(S,O) level. Additional information is presented in the Computational Details Section and the Supporting Information, including benchmarking studies to evaluate the functional sensitivity and solvent effects on geometries and energies (Tables S2–S3).

### Acylation Reaction Mechanism

**Figure 4:**
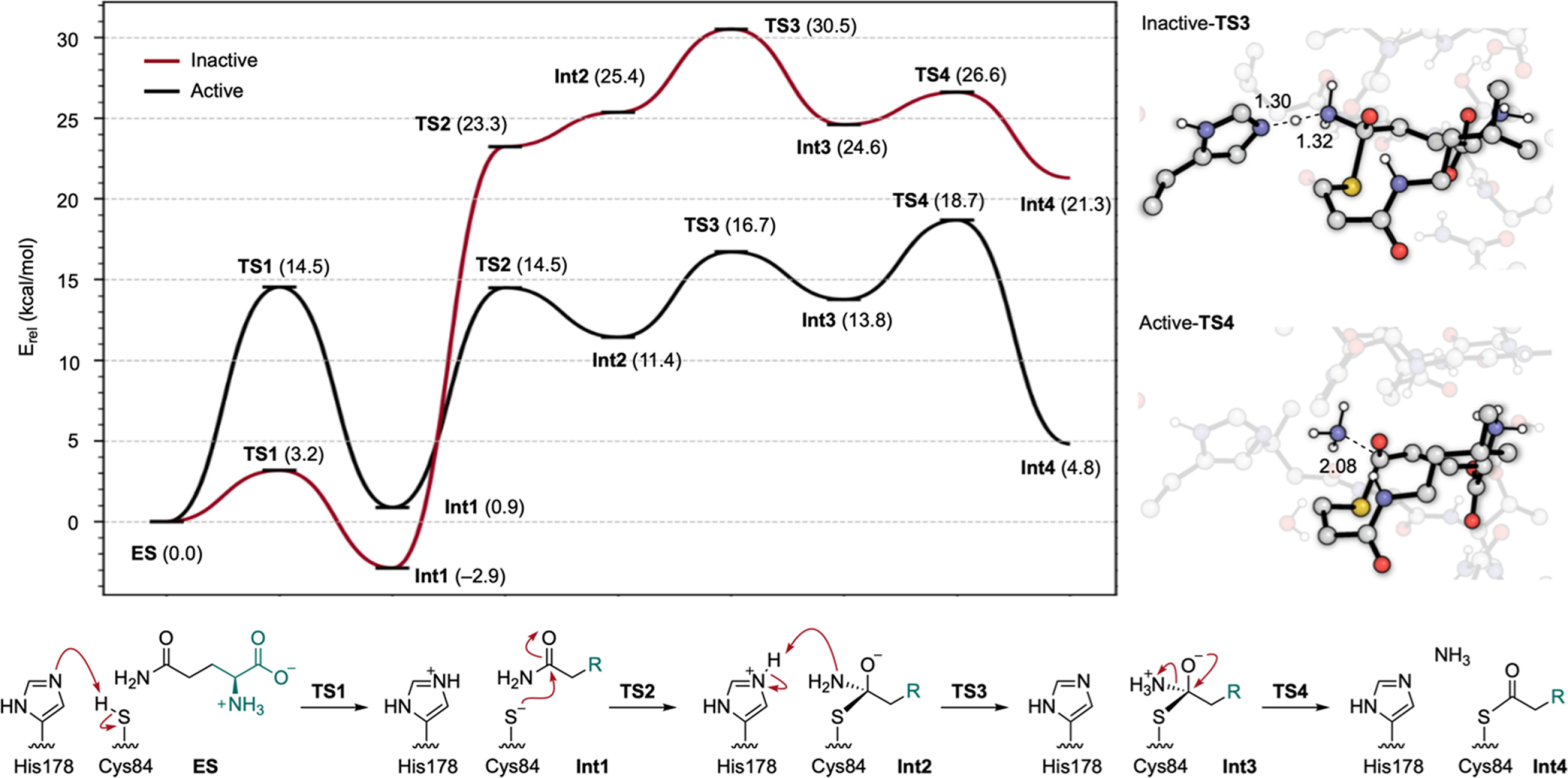
Energy profiles of active (black) and inactive (red) IGPS active site geometries in kcal/mol. Representations of the rate-limiting steps corresponding to each model with TS bonds are shown as dotted lines and distances in Å.

The thioester formation mechanism proposed in this work (Figure 4) begins from the enzyme–substrate (**ES**) complex with deprotonation of Cys84 by His178 to form the thiolate in **Int1**. The nucleophilic Cys84 attacks the substrate carbonyl, forming a tetrahedral oxyanion intermediate (**Int2**). The His178 imidazolium transfers a proton to the substrate to form an ammonium in (**Int3**). Lastly, the tetrahedral intermediate collapses, and the N–C bond breaks, forming the thioester acyl-intermediate and liberating ammonia (**Int4**). XYZ coordinates for all evaluated structures are provided in the Supporting Information, along with images of each TS with labeled bonds breaking and/or forming. A PyMol visual session of all aligned structures and a movie of the elementary steps can be found online in the GitHub repository (see Associated Content).

The electronic energy profiles of the Active and Inactive models (Figure 4) illustrate how differences in the active site geometry substantially influence the reaction. Deprotonation of Cys84 in Active has a barrier of 14.5 kcal/mol, significantly higher than the 3.2 kcal/mol required for Inactive. This difference is due to the positioning of the Gln substrate. In the optimized Active-**ES**, the substrate is observed in a near-attack conformation, with the carbonyl carbon 3.56 Å from the Cys84 sulfur and the NH_2_ group hydrogen bonding with the His178 Nε (2.01 Å). This interaction is disrupted in Active-**TS1** when the His178 Nε accepts the proton from Cys84. Alternatively, in Inactive-**ES** the substrate is positioned farther away (3.84 Å), which enables Cys84 to hydrogen bond with His178 (1.91 Å). The Gln pose optimized in Inactive is not possible in Active because the proximity of *f*Gln123 confines the substrate.

The rate of thioester formation in the Active geometry is determined by the collapse of the tetrahedral oxyanion intermediate with a TS bond-breaking distance of 2.08 Å (Active-**TS4**, Figure 4). This yields a computationally predicted barrier of Δ*E*^‡^ = 18.7 kcal/mol, which agrees well with experimental *k*_cat_ values of *T. maritima* (0.67 ± 0.02 s^–1^ at 298.15 K,^6^ corresponding to a barrier of around 17.7 kcal/mol up to 5.92 ± 0.18 s^–1^ at 343.15 K,^58^ corresponding to a barrier of around 19.0 kcal/mol). Although temperature effects are not considered in this study, previous work has found that at temperatures near the native*T. maritima* environment (343 K), the apo and PrFAR bound states exhibit similar levels of conformational flexibility measured by NMR spectroscopy; however, this does not translate to equal catalytic activities.^58^ Isotope effects of the amide nitrogen on glutamine bound to a similar class-I GAT, carbamoyl phosphate synthetase (CPS), support the release of ammonia as the rate-limiting step in the presence of reaction activators MgATP and HCO_3_^–^ (see the Supporting Information for a more in-depth discussion).^11^

In the Inactive geometry, the formation of ammonium (Inactive-**TS3**, Figure 4) is the highest in energy, yielding a barrier of Δ*E*^‡^ = 30.5 kcal/mol for thioester formation. This barrier is larger than expected from the experimental measurements across temperature ranges 298.15–343.15 K (20.1 to 22.1 kcal/mol).^6,58^ Alternative pathways were explored, such as concerted NH_3_ formation and tetrahedral collapse, but the corresponding stationary points were not located. Another possibility is that more energetically favorable structural rearrangements occur along the reaction coordinate to enable catalysis. In support of this, the barrier for the Val51 backbone flip in the absence of PrFAR is estimated by steered MD to be approximately 22 kcal/mol,^36^ which aligns well with the experimental rate (Figure S4). Therefore, our current DFT results suggest that glutamine hydrolysis in the absence of the allosteric effector is limited by the barrier for conformational conversion rather than by the elementary bond-forming and bond-breaking steps in the active site.

The elementary step most affected by active site structural change is the formation of the tetrahedral acylenzyme via **TS2**. This transformation requires 13.6 kcal/mol from **Int1** in Active and nearly twice as much in Inactive (26.1 kcal/mol). This step demonstrates the importance of the IGPS active site geometry in stabilizing the incipient oxyanion. Although it has been proposed that the IGPS structure 3zr4 (structurally consistent with our Inactive model) is in a catalytically competent conformation with the Gly52 N–H completing the oxyanion hole, our data instead indicate that the energy associated with the Inactive geometry hinders thioester formation.

There are multiple structural differences between the Active and Inactive models (Figures S1 and S8). These include not only the Val51 backbone flip and *f*Gln123 proximity but also less apparent variations, such as the relative position of the oxyanion strand. The capacity of Gly52 to serve as an H-bond donor along the reaction coordinate may depend on the subtle positioning of the oxyanion strand. Additionally, it is unclear from the energy profiles alone how these geometric differences influence the reaction. We therefore pursued additional computational studies to deconvolute the energetic effects due to specific structural variations in the IGPS acylation reaction.

### Oxyanion Hole Stabilization

To analyze how the *f*Gln123 proximity, Val51 backbone conformation, and position of the oxyanion strand separately influence TS stabilization in the Active model, we constructed two additional models, referred to as Inactive *f*Gln123 and Inactive Val51. To construct Inactive Val51, the Active model was altered by manually flipping the Val51 amide such that N–H is directed away from the binding site (Figure S2), mimicking this aspect of the Inactive conformation. Inactive *f*Gln123 was built by aligning the coordinates of Cys84 S and reactive Gln C, N, and Oε atoms of the optimized Active- and Inactive-**TS3** structures. After alignment, the atomic coordinates corresponding to the molecular fragment containing *f*Gln123 in Active were replaced with the corresponding Inactive coordinates (Figure S3).

**Figure 5:**
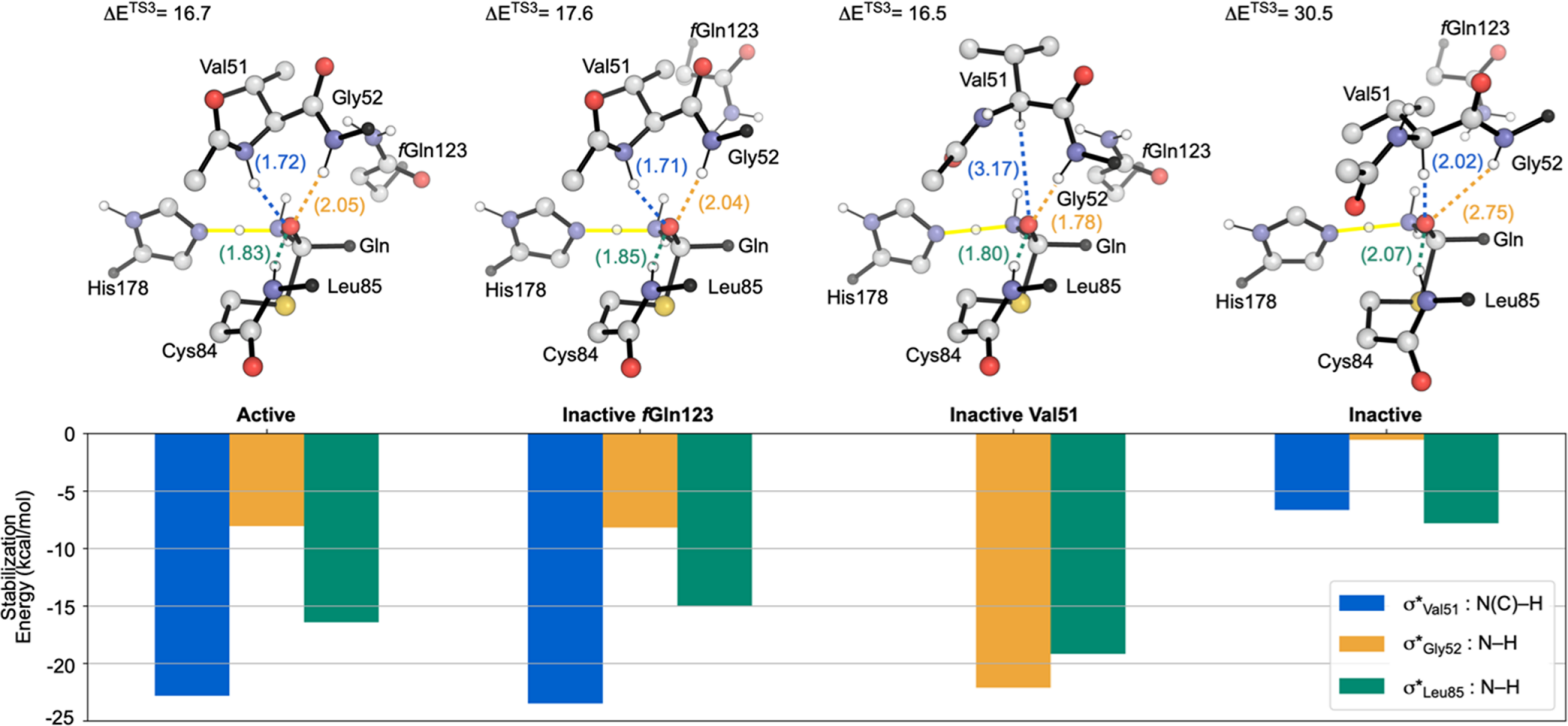
Oxyanion stabilizing interaction energies between Gln Oε lone pair electrons and σ* orbitals from Val51 (blue), Gly52 (orange), and Leu85 (green) in **TS3** measured with NBO.^59^ Reduced ball and stick representations of the Active-, Inactive *f*Gln123-, Inactive Val51-, and Inactive-**TS3** structures of relevant model components are included to aid visualizations. Black spheres indicate where residues were cut off for the image. Full 237-atom structure representations of each TS are provided in the Supporting Information. Distances between the Gln Oε and backbone H atoms are shown in Å.

This approach enables us to quantify the influence of each structural difference relative to the Active model separately. The oxyanion hole contributions were evaluated in each TS by estimating the donor–acceptor orbital stabilization energies of the Leu85, Gly52, and Val51 σ* orbitals and the Gln Oε lone pair electrons via natural bonding orbital (NBO) analysis (Figure 5).^59^ Energy barriers and stabilization energies not provided here can be found in the Supporting Information (Figures S5, S6 and Table S4).

As hypothesized, Val51 is a predominant component of the oxyanion hole structure when the Val51 N–H points toward the substrate. In Active- and Inactive *f*Gln123-**TS3**, the Val51 N–H σ* provides a favorable interaction energy of around −23 kcal/mol. Leu85 and Gly52 also contribute to oxyanion stability in these structures, however, to lesser extents. Importantly, the proximity of *f*Gln123 does not alter the oxyanion hole or the overall energetics in any of the evaluated TSs. Decomposition of the *f*Gln123 interaction energy reveals that its proximity does not aid any individual chemical step (see the Supporting Information for a more in-depth discussion and calculation details). Nevertheless, the Active and Inactive Val51 models reveal a more energetically favorable *f*Gln123 position compared to those of Inactive *f*Gln123 and Inactive.

There is a substantial difference in the models with Val51 N–H pointing away from the substrate. The oxyanion hole structure is ineffective in Inactive-**TS3** with nearly no interaction from Gly52, Leu85 contributing only −7.8 kcal/mol, and Val51 Cα–H contributing −6.7 kcal/mol. Although C–H bonds are not often considered hydrogen bond donors, the Val51 Cα–H in the Inactive model is positioned to stabilize the Gln *O*ε. Precedence for C–H oxyanion hole structures has been reported for a class of *cinchona* alkaloid catalysts.^60^

Alternatively, the Inactive Val51 conformation provides an adequate oxyanion hole without involving Val51. Instead, the Gly52 N–H is the most prevalent component, contributing −22.1 kcal/mol, an amount comparable to that of the Val51 N–H in the Active and Inactive *f*Gln123 models. Even though there are only two residues contributing to oxyanion stability in Inactive Val51, this conformation is as reactive as in the Active and Inactive *f*Gln123 conformations. Furthermore, the Inactive Val51 model shows that the Val51 dihedral flip is not the only conformational change that can explain the rate enhancement observed in IGPS. In the initial Inactive Val51 model, the Val51 φ dihedral matches that of the optimized Inactive-**ES** structure (−138°) by construction. During geometry optimizations, the dihedral adjusts to −111° in Inactive Val51. This adjustment is consistent with the description of a third oxyanion strand conformation identified in MD simulations as an intermediate conformation of the Val51 dihedral flip.^36^ The partial dihedral rotation introduces a weak interaction (less than 0.5 kcal/mol) between the Gln Oε lone pair electrons and the π* orbital of backbone Gly50 C=O. This type of *n*–π* interaction to stabilize an oxyanion tetrahedral intermediate has precedence in aspartic proteases^61^ and, therefore, could be a catalytically relevant state in IGPS.

From the energetic perspective, the Active, Inactive *f*Gln123, and Inactive Val51 models are consistent with experimental kinetic data. Two other class-I GATs, pyridoxal 5′-phosphate (PLP, PDB: 2NV2(31)) synthase and carbamoyl phosphate synthetase (CPS, PDB: 1C3O(62)), have been crystallized in their catalytic forms with the Gln substrate bound. Upon alignment of the Gln substrate reactive carboxamide, the oxyanion strand residue involved in the preformed oxyanion holes in PLP synthase and CPS is more structurally consistent with the IGPS Gly52 than Val51 in Active-**ES** (Figure S10). Alignment with Inactive Val51-**ES** shows an even closer agreement, providing evidence that this manually constructed geometry of the IGPS glutaminase active site is feasible. Lastly, glutaminase activation via oxyanion strand repositioning has been proposed for another class-I GAT, aminodeoxychorismate synthase (ADCS), which displays an allosteric response similar to IGPS.^43^ Cumulatively, these data are consistent with the computed mechanism and transition structures proposed here.

## Conclusions

This work provides new insights into local active site structural changes that yield energetics consistent with experimentally measured allosteric rate acceleration in IGPS. For a summary of the work presented here in relation to previous studies, see Figure S4. The hypothesized Val51 backbone flip leads to a more favorable reaction pathway via oxyanion hole stabilization, supporting the longstanding hypothesis to explain the observed allosteric effect. However, this work reveals that this is not the only plausible configuration to yield the expected catalytic effect, as the Gly52 N–H is also a capable oxyanion hole contributor. Furthermore, the inactive conformation yields a calculated reaction barrier beyond that expected from experimental measurements, suggesting a conformational change as the rate-limiting step in the absence of PrFAR.

Oxyanion stabilization via Gly52 has been proposed previously;^42^ however, most studies to date have focused on the Val51 dihedral flip. When the Val51 backbone of the Active model is manually modified to mimic the Inactive structure, the Gly52 N–H becomes a suitable oxyanion hole donor. These results show that multiple active site conformations are catalytically competent and reveal that the positioning of the oxyanion strand is a definitive structural change required for proper oxyanion hole formation.

The truncated active site approach uniquely simplifies the geometric modification of distinct structural features to facilitate the evaluation of their individual effects. Such modifications would be more challenging with modeling approaches that explicitly treat the entire protein environment (e.g., QM/MM). Alternatively, multiscale methods are better suited to model the interconversions of these different geometries to more directly investigate the connection between the conformational and chemical coordinates, which continues to be an important challenge in the field.^44^ The approach applied in this work is reminiscent of theozyme modeling,^64^ where residues or functional groups are hypothetically arranged in a truncated active site to explore catalytic contributions of individual atomic interactions and predict optimal catalytic scaffolds. The design of the Inactive Val51 and Inactive *f*Gln123 models enabled us to quantify the influence of each structural difference relative to the Active geometry. This approach is generalizable to other systems in efforts to probe the energetic influence of local structural aspects and highlights the unique applications available to theozyme and cluster modeling.

## Computational Details

Geometry optimizations were performed using Gaussian 16, Revision C.01^65^ with the range-separated, dispersion-corrected ωB97X-D^66^ functional using the 6-31G*^67^ basis set for all C, H, and N atoms and the 6-31+G*^68^ basis set for S and O atoms. The Cα of selected protein residues was frozen during optimizations to conserve the active site geometry in the absence of the greater protein environment. All geometries were inspected for structural distortions that could have occurred during optimization. Vibrational frequencies were computed to confirm the nature of minima (all real normal modes) and transition structures (a single imaginary normal mode). Stationary point electronic energies were further refined at the B3LYP-D3(BJ)/6-311+G(2d,2p) level of theory and the default IEF–PCM implicit solvent method with internal parameters for diethyl ether (ε = 4.24).^69−74^ This procedure is commonly applied in enzymatic QM cluster modeling.^48,75,76^ GoodVibes software was used to generate electronic energy profiles.^77^ The energetic span^78^ between the lowest energy intermediate and the highest energy transition structure (TS) was used to estimate the activation energy. Due to the necessary frozen constraints during geometry optimizations, the vibrational partition function is not considered reliable. Therefore, we focus on the electronic energy profile (i.e., rather than the Gibbs energy) throughout. This approximation is common in truncated enzyme modeling and has shown success in mechanistic investigations.

The oxyanion hole strength in **TS2**, **TS3**, and **TS4** of every model was assessed using the natural bond orbital (NBO) analysis program (version 7.0.5).^59^ NBO analysis transforms the optimized atomic orbital basis set into a localized ideal Lewis structure basis. Second-order perturbation theory analysis of the Fock matrix on the basis of the NBO is used to estimate the stabilization energy associated with electron delocalization from a filled donor NBO to an unfilled acceptor NBO. The default interaction energy minimum threshold of 0.05 kcal/mol was used. The oxyanion hole interaction energies were investigated between Gln Oε lone pair electrons and the σ* orbitals of the backbone N–H of Leu85, Gly52, and Val51. Additionally, Val51 Cα–H was investigated in Inactive Val51 and Inactive.

## Data Availability

Raw data of all calculations, the corresponding software keywords and versions, aligned *xyz* coordinates of all evaluated structures, the energy profiles of the Active and Inactive models, and additional single-point energy calculations at ωB97X-D/def2QZVPP with IEF–PCM diethyl ether are publicly accessible at https://github.com/hklem/IGPS_QM_cluster_models.

## Supporting Information Available

IGPS active site model components and protonation states; visual comparisons of each model side by side with the Active conformation; evaluation of the level of theory; summary of this work in relation to previous studies; NBO calculation details and extended data; ALMO-EDA of *f*Gln213 calculation details and data; absolute energies from geometry optimizations and single point corrections for all evaluated structures; visual representations of optimized geometries and relevant atomic distances of each evaluated transition state; structural comparisons with PLP synthase and CPS; and *xyz* coordinates of all evaluated geometries (PDF)
