# Supplementary material for: Catalytic Effects of Active Site Conformational Change in the Allosteric Activation of Imidazole Glycerol Phosphate Synthase
Source: ACS Catal. 2023 Dec 6;13(24):16249–57. doi: 10.1021/acscatal.3c04176 (PMC10729027; doi:10.1021/acscatal.3c04176)
Supplement: Supplementary file 1 — cs3c04176_si_001.pdf [file cs3c04176_si_001.pdf]

Supporting Information

The catalytic effects of active site conformational change in the allosteric activation of imidazole glycerol phosphate synthase

Heidi Klem,[1,2,*] Juan V. Alegre-Requena,[3,*] Robert S. Paton[1,*]

[1] Department of Chemistry, Colorado State University, Fort Collins, CO 80523, USA

[2] Current address: Thermodynamics Research Center, Applied Chemicals and Materials Division, National Institute of Standards and Technology, Boulder, CO 80305, USA

[3] Dpto.de Química Inorgánica, Instituto de Síntesis Química y Catálisis Homogénea (ISQCH), CSIC, Universidad de Zaragoza, Zaragoza 50009, Spain

*Correspondence: heidi.klem@nist.gov; jv.alegre@csic.es; robert.paton@colostate.edu

Contents

IGPS active site model components and protonation states **2**

Model construction and structural comparisons **3**

Evaluation of level of theory **4**

Summary of this work in relation to previous studies **5**

NBO calculation details and data **6**

Decomposition of *f*Gln123 Interaction Energy **9**

Absolute energies for all evaluated structures **12**

Geometries and relevant atomic distances of each evaluated transition structure **13**

Structural comparison with PLP synthase **15**

XYZ coordinates of optimized stationary points **16**

References **40**

IGPS active site model components and protonation states

Table S1. IGPS active site models from PDB 7ac8 chains C/D (Inactive model) and E/F (Active model)

Number	Name	Chain	Charge	Side chain	Main chain	Atom constraints
121	Gly	E (C)	0	Kept	Cut at N-terminus	Cα
122	Ser	E (C)	0	Removed	Kept	None
123	Gln	E (C)	0	Kept	Kept	None
124	Ala	E (C)	0	Removed	Cut at C-terminus	Cα
50	Gly	F (D)	0	Kept	Cut at N-terminus	Cα
51	Val	F (D)	0	Kept	Kept	None
52	Gly	F (D)	0	Kept	Kept	None
53	His	F (D)	0	Removed	Cut at C-terminus	Cα
A84C	Cys	F (D)	0	Kept	Cut at N-terminus	Cα
85	Leu	F (D)	0	Kept	Cut at C-terminus	Cα
88	Gln	F (D)	0	Kept	Removed	Cα
96	Glu	F (D)	–1	Kept	Removed	Cα
140	Val	F (D)	0	Kept	Cut at N-terminus	Cα
141	His	F (D)	0	Removed	Kept	None
142	Thr	F (D)	0	Kept	Kept	None
143	Tyr	F (D)	0	Kept	Cut at C-terminus	Cα
178	His	F (D)	0	Kept	Removed	Cα
180	Glu	F (D)	–1	Kept	Removed	Cα
425 (418)	HOH	F (D)	0	–	–	–
426 (420)	HOH	F (D)	0	–	–	–
301	L-Gln	Substrate	0	–	–	–

Components of HisF residues 121, 122 and 124 were included to expand the main chain around *f*Gln123 and allow the terminal C*α* atoms of this molecular fragment to be frozen. This is done to give the *f*Gln123 residue slight flexibility in geometry optimizations. We included the backbone atoms of His141 since Val140 and Thr142 were also included in the model, therefore we felt truncating at each of those C*α* would be more artificial than keeping the His141 backbone and removing the sidechain. Likewise, the portion of the His53 backbone adjacent to Gly52 was kept. This was also decided since we wanted to allow for some flexibility in the oxyanion strand, so we froze the His53 C*α*, and left the Gly52 C*α* unrestrained. Atoms were constrained using the "–1" optimization flag syntax in Gaussian. This enforces the gradient of these atoms to be 0 during the optimization updates.

The initial protonation states are assumed since hydrogen atoms are not resolved in the X-ray crystal structure. The His178 Nδ protonation state is supported given the position relative to Glu180 in numerous IGPS crystal structures and the expected general acid/base behavior of histidine in enzyme mechanisms involving cysteine catalytic triads.[1] The protonation state of Cys84 is interesting to consider, because it is not necessarily required to reform in the catalytic cycle. Therefore, it is possible Cys84 protonation is obviated during multiple turnover events. A hybrid QM/MM study of a cysteine protease acylation mechanism found the ionic state of the Cys–His dyad (Cys^{-}, $HisH^{+}$) to be more stable in the free enzyme, whereas binding of the substrate shifted the equilibrium such that the neutral dyad became more stable.[2]

Glu180 hydrogen bonds with His178 in all structures and remains in the carboxylate form for the results discussed here. **Int1** geometries with a Glu180 carboxylic acid were evaluated and the difference in protonation was not found to alter any mechanistic conclusions. Furthermore, site-directed mutagenesis experiments support a mechanism where Glu180 serves a structural role to position His178, as opposed to directly effecting catalysis.[3]

Model construction and structural comparisons

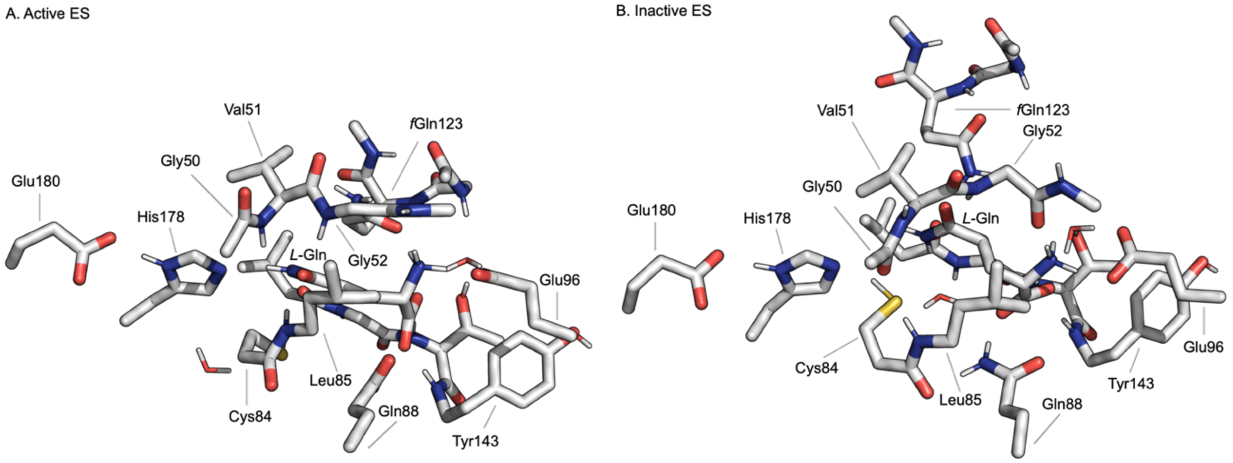

Figure S1. Geometric comparisons of the (A) Active and (B) Inactive models. Coordinates from each model are taken from their optimized **ES** structures. Inactive differs from Active mainly in the proximity of *f*Gln123, positioning of the oxyanion strand, and backbone dihedral of Val51.

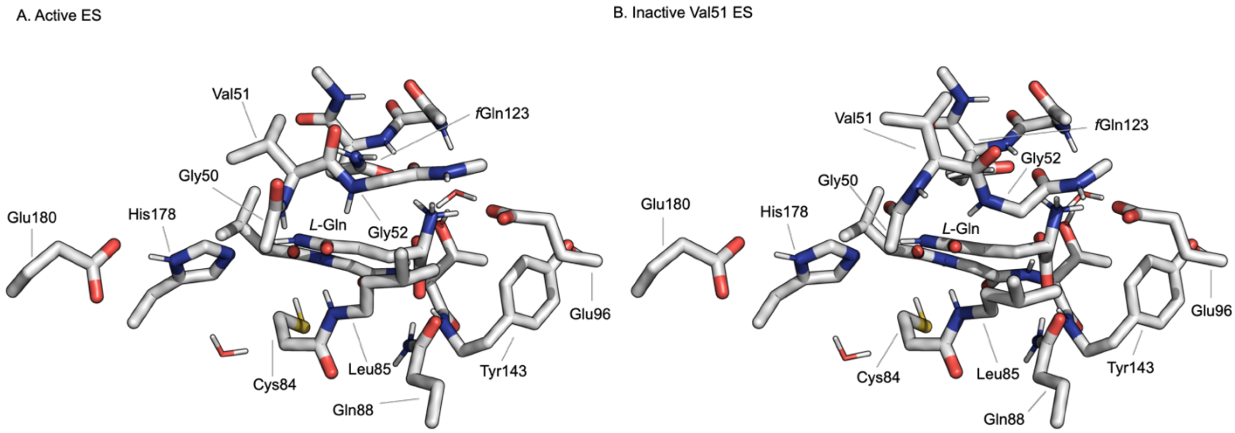

Figure S2. Geometric comparisons of the (A) Active and (B) Inactive Val51 models. Coordinates from each model are taken from their optimized **ES** structures. Inactive Val51 differs from Active in the backbone dihedral of Val51. All carbons frozen during optimization are in the same positions between the two models.

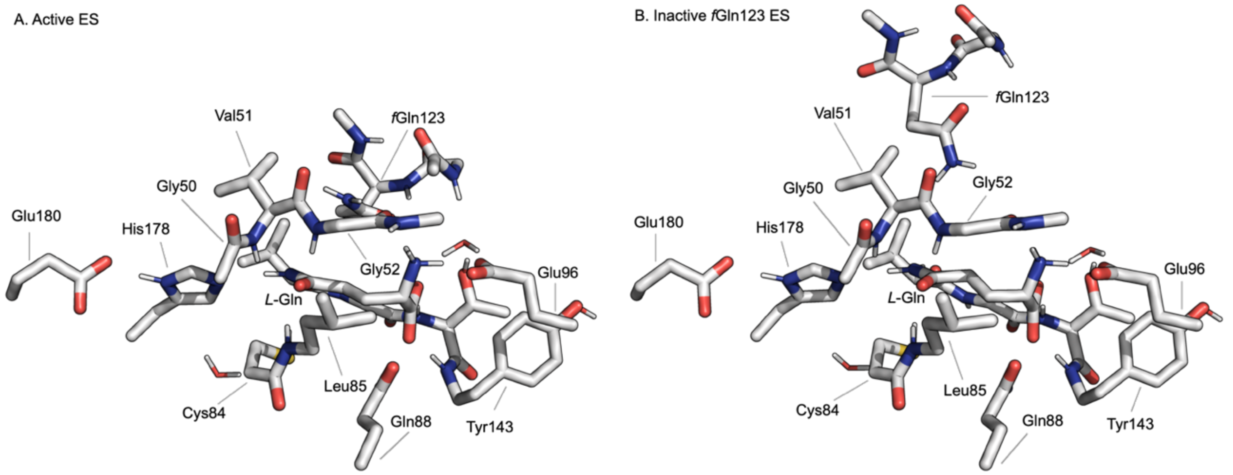

Figure S3. Geometric comparisons of the (A) Active and (B) Inactive *f*Gln123 models. Coordinates from each model are taken from their optimized **ES** structures. Inactive fGln123 differs from Active in the proximity of *f*Gln123.

Evaluation of level of theory

Table S2. Geometry optimizations (GO) at different levels of theory. Energies shown in kcal/mol. RMSDs calculated from all atoms using VMD and shown in Å.[4]

Structure	GO1 (relative)	GO2 (relative)	GO3 (relative)	RMSD GO2 and GO1	RMSD GO3 and GO1
Active-**ES**	-3581009.492 (0.00)	-3582265.226 (0.00)	-3581119.545 (0.00)	0.074	0.207
Active-**TS3**	-3580995.885 (13.61)	-3582252.222 (13.00)	-3581106.087 (13.46)	0.067	0.087
Active-**TS4**	-3580989.797 (19.70)	-3582248.267 (16.96)	-3581100.402 (19.14)	0.083	0.107

Legend:
GO1: ωB97X-D/6-31G* on C, H, N and 6-31+G* on S, O atoms
GO2: B3LYP-D3BJ/6-31G* on C, H, N and 6-31+G* on S, O atoms
GO3: ωB97X-D/6-31G* on C, H, N and 6-31+G* on S, O atoms, using CPCM with solvent=diethylether

Table S3. Single point (SP) corrections at different levels of theory. Energies shown in kcal/mol.

Structure	GO1//SP1 (relative)	GO1//SP2 (relative)	GO1//SP3 (relative)	GO2//SP1 (relative)	GO3//SP1 (relative)
Active-**ES**	-3583496.160 (0.00)	-3582171.337 (0.00)	-3582628.241 (0.00)	-3583495.593 (0.00)	-3583497.955 (0.00)
Active-**TS3**	-3583479.458 (16.70)	-3582154.165 (17.17)	-3582609.427 (18.81)	-3583479.014 (15.58)	-3583480.553 (17.40)
Active-**TS4**	-3583477.492 (18.67)	-3582149.758 (21.58)	-3582605.099 (23.14)	-3583476.681 (18.91)	-3583478.296 (19.66)

Legend:
SP1: B3LYP-D3BJ/6-311+G(2d,2p), using IEF-PCM with solvent=diethylether
SP2: ωB97X-D/6-311+G(2d,2p), using IEF-PCM with solvent=diethylether
SP3: ωB97X-D/def2QZVPP, using IEF-PCM with solvent=diethylether

The Active-**ES**, Active-**TS3** and Active-**TS4** structures were selected to evaluate the functional sensitivity and solvent effects on geometries and energies. The energies reported in the main text are obtained from the GO1//SP1 level. This was chosen since it was the most consistent with the available experimental data. In all variations **TS4** is identified as the rate limiting step.
The ωB97X-D (GO1) and B3LYP-D3BJ (GO2) geometry optimizations are consistent in both structure (all atom RMSDs < 0.1 Å) and energies ($\Delta\Delta E < 1$ kcal/mol for **TS3** and **TS4**). The difference in barriers from these optimized geometries after single point energy refinement (GO1//SP1 and GO2//SP1) are 1.12 kcal/mol and 0.24 kcal/mol for **TS3** and **TS4**, respectively, and **TS4** remains the rate-limiting TS.

The standard, and generally well-accepted, approach in truncated enzyme models is to perform gas phase geometry optimizations.[5] Nevertheless, solvent effects on the geometries may be important to consider especially for charge-separated transition states. Therefore, we reoptimized the **ES**, **TS3** and **TS4** of the Active model with implicit solvent. We encountered numerous convergence issues while attempting to optimize with the IEF-PCM method, however we were successful with CPCM (solvent = diethylether). The difference in energy barriers measured from **ES** when including solvent effects (GO3) is very minimal ($\Delta\Delta E^{TS3} = -0.15$ kcal/mol and $\Delta\Delta E^{TS4} = -0.56$ kcal/mol), even though there are subtle structural changes, as measured by all-atom RMSDs: **ES**=0.207, **TS3**=0.087, **TS4**=0.107. After single-point energy corrections on the solvent optimized geometries (GO3//SP1) there are minimal energy differences ($\Delta\Delta E^{TS3} = 0.70$ kcal/mol and $\Delta\Delta E^{TS4} = 0.99$ kcal/mol) that do not alter any discussion or conclusions in the main text.

Summary of this work in relation to previous studies

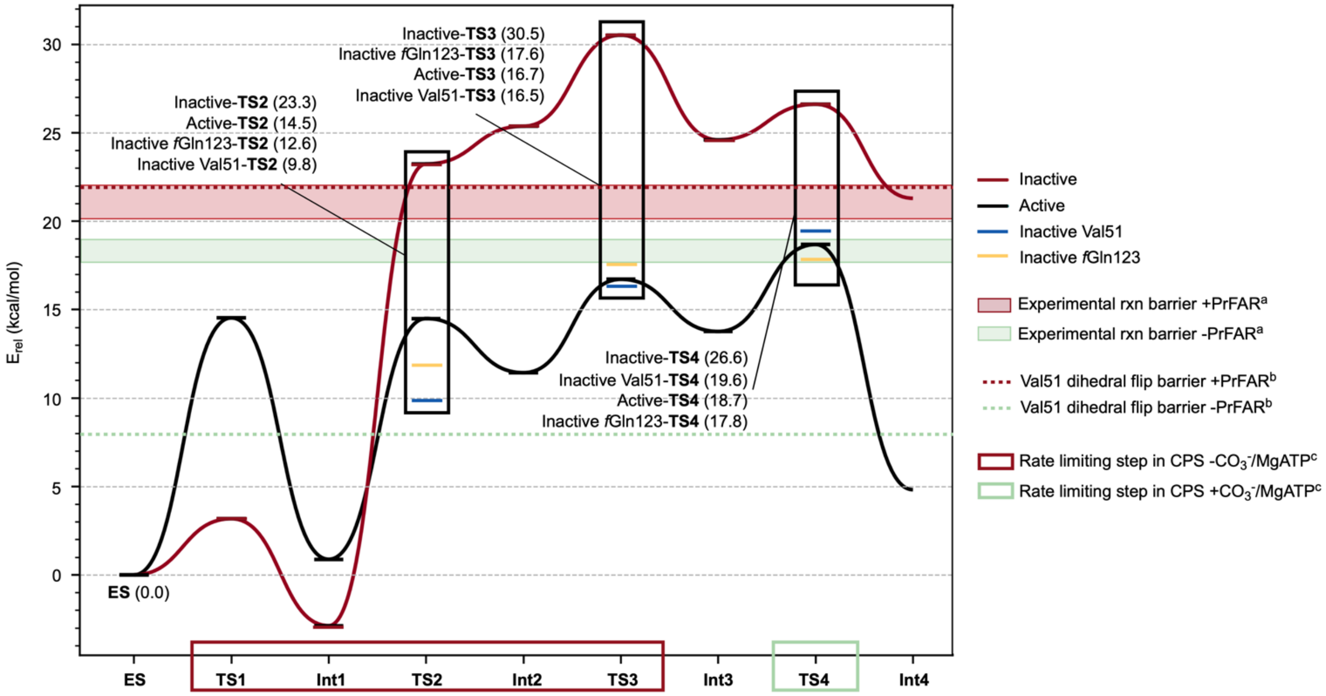

Figure S4. Energy surface summary of this work in relation to previous studies.

[a] Glutamine hydrolysis reaction barriers corresponding to experimental rate kinetics performed in the absence of PrFAR (red shaded region) and in the presence of PrFAR (green shaded region).[6,7]

[b] Steered molecular dynamics studies performed by Osuna and coworkers[8] evaluated the free energy barrier of a Val51 dihedral flip in IGPS simulations of varying substrate bound states. They estimated the conformational barrier to be 22 kcal/mol in the -PrFAR/+Gln state (red dashed line), consistent with the basal activity measured from experiments. Their result in conjunction with the high Inactive barrier calculated here support a basal reaction that is rate limited by conformational change. In the +PrFAR/+Gln state the Val51 dihedral rotation barrier was reduced to 8 kcal/mol (green dashed line), much lower than the experimental Gln hydrolysis rate indicating a chemical transformation is rate limiting in the presence of PrFAR.

[c] The ^{15}N kinetic isotope effect (KIE) was measured in carbamoyl phosphate synthetase (CPS), a class-I GAT similar to IGPS, to investigate the rate limiting step of glutamine hydrolysis in the presence and absence of the reaction activators CO_3^- and MgATP.[9] The glutamine substrate ^{15}N KIE in the presence of activators was measured to be 1.023, which is close to the KIE of 1.025 observed from organic model reactions in which the C–N bond breaking is known to be rate-determining.[10] This translates to **TS4** as the rate limiting step in Gln hydrolysis by IGPS in the presence of PrFAR (green box). In the absence of the CPS activators, the ^{15}N isotope effect is reduced by 1.57%, and the authors predict the rate limiting step to occur prior to ammonia release (red box).

NBO calculation details and data

The **ES**, **TS2**, **TS3** and **TS4** stationary points were optimized in Inactive *f*Gln123 and Inactive Val51. Energy barriers were calculated for each TS relative to the **ES** structure of the corresponding model. The stabilizing oxyanion hole strength was evaluated in each TS by donor-acceptor interactions of Leu85, Gly52 and Val51 with the Gln Oε lone pair electrons via Natural Bonding Orbital (NBO) second-order perturbation theory analysis version 7.0.5.[11]

The stabilization energies of oxyanion hole residues in **TS2** (Figure S4) are highly consistent with the results from **TS3** shown in the main text, however since only two Gln Oε lone-pair electrons were identified in each **TS2** structure the total contributions from each residue are smaller in magnitude compared to the **TS3** results with three lone-pairs.

The stabilization energies of oxyanion hole residues in **TS4** (Figure S5) reveal that the Val51 N–H in the Active and Inactive *f*Gln123 models no longer interacts with Gln Oε lone-pair electrons. Instead, the N–H shifts to interact with the new lone pair electrons on the His178 Nε. This results in oxyanion hole stabilization energy contributions to be nearly identical in Active-**TS4**, Inactive fGln123-**TS4** and Inactive Val51-**TS4**.

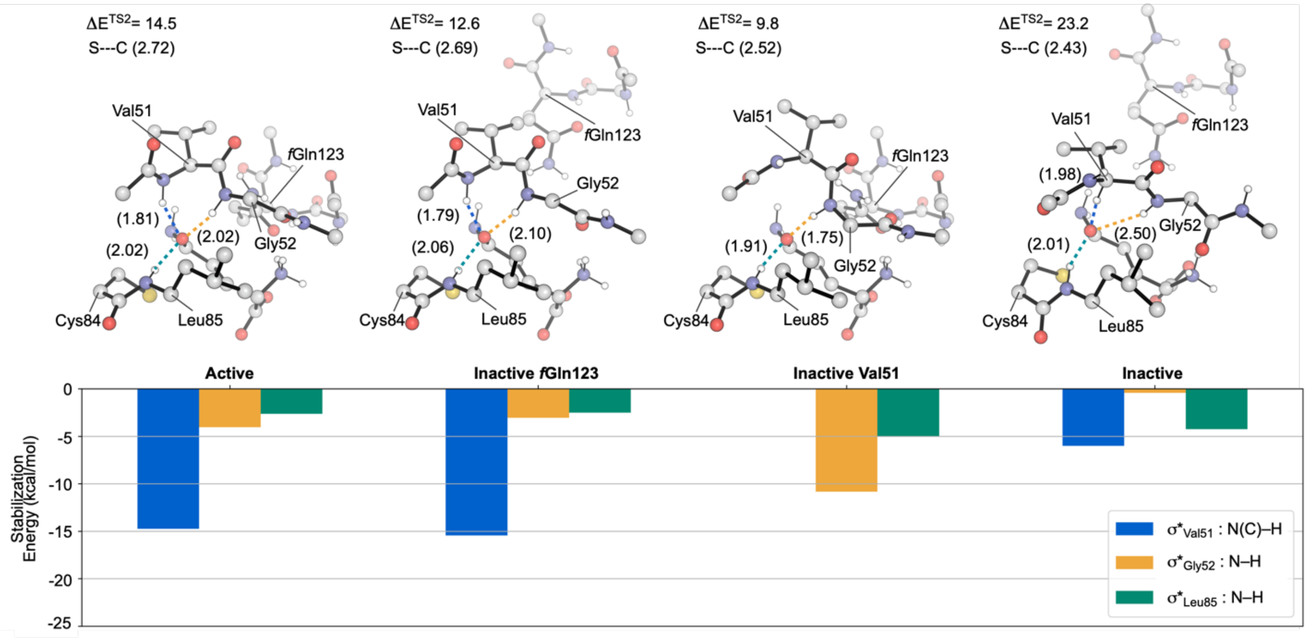

Figure S5. Oxyanion stabilizing interaction energies between Gln Oε lone pair electrons and σ* orbitals from Val51, Gly52 and Leu85 in **TS2** measured by second order perturbations in the NBO basis. Reduced ball and stick representations of the Active, Inactive *f*Gln123, Inactive Val51 and Inactive **TS2** structures of relevant model components to aid visualizations. Distances between the Gln Oε and backbone H atoms of Val51, Gly52 and Leu85 are shown in Å. Active and Inactive *f*Gln123 correspond to models with the Val51 N–H pointing into the active site. Inactive Val51 and Inactive correspond to models with the Val51 N–H directed outwards.

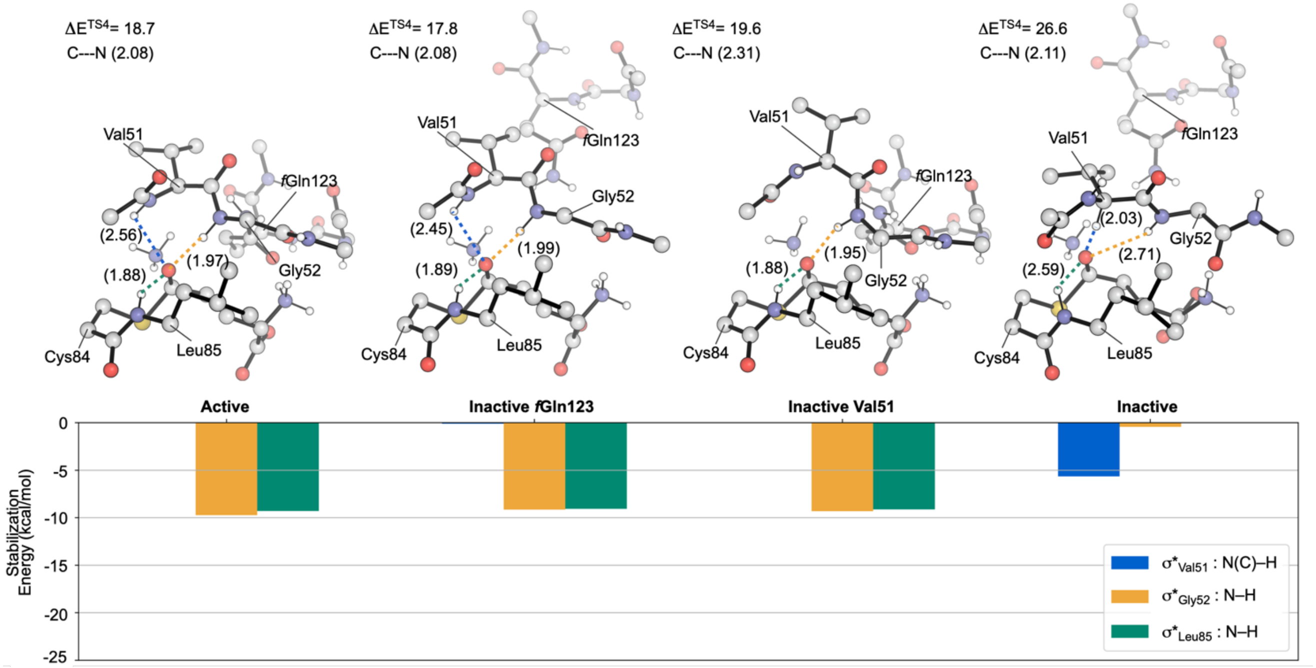

Figure S6. Oxyanion stabilizing interaction energies between Gln Oε lone pair electrons and σ^* orbitals from Val51, Gly52 and Leu85 in **TS4** measured by second order perturbations in the NBO basis. Reduced ball and stick representations of the Active, Inactive *f*Gln123, Inactive Val51 and Inactive **TS4** structures of relevant model components to aid visualizations. Distances between the Gln Oε and backbone H atoms of Val51, Gly52 and Leu85 are shown in Å. Active and Inactive *f*Gln123 correspond to models with the Val51 N–H pointing into the active site. Inactive Val51 and Inactive correspond to models with the Val51 N–H directed outwards.

Table S4. Interactions between oxyanion hole components and Gln Oε lone pair electrons. A value of 0 arises if there is either no interaction or the strength of that interaction is below the calculation cutoff (0.05 kcal/mol). “n/a” indicates the corresponding lone pair electrons were either not detected in the structures or were delocalized into antibonding orbitals connected to the Gln substrate.

**Model**	**Structure**	**Residue**	**Orbital**	**Interaction with LP 1 (kcal/mol)**	**Interaction with LP 2 (kcal/mol)**	**Interaction with LP 3 (kcal/mol)**	**Total contribution (kcal/mol)**
Active	TS2	Val51	BD*(1) N 22- C 23	4.25	10.49	n/a	14.74
Active	TS2	Gly52	BD*(1) N 29- H224	3.80	0.24	n/a	4.04
Active	TS2	Leu85	BD*(1) N 39- H154	2.63	0	n/a	2.63
Active	TS3	Val51	BD*(1) N 98- H232	7.19	11.31	4.31	22.81
Active	TS3	Gly52	BD*(1) N105- H228	3.43	1.49	3.13	8.05
Active	TS3	Leu85	BD*(1) N 5- H 53	7.71	0	8.70	16.41
Active	TS4	Val51	BD*(1) N202- H236	0	0	n/a	0
Active	TS4	Gly52	BD*(1) N203- H232	3.88	5.84	n/a	9.72
Active	TS4	Leu85	BD*(1) N 44- H 68	5.09	4.21	n/a	9.30
Inactive fGln123	TS2	Val51	BD*(1) N118- H152	4.79	10.64	n/a	15.43
Inactive fGln123	TS2	Gly52	BD*(1) N119- H148	2.74	0.30	n/a	3.04
Inactive fGln123	TS2	Leu85	BD*(1) N190- H205	2.51	0	n/a	2.51
Inactive fGln123	TS3	Val51	BD*(1) N161- H195	7.02	11.62	4.83	23.47
Inactive fGln123	TS3	Gly52	BD*(1) N162- H191	3.56	1.46	3.15	8.17
Inactive fGln123	TS3	Leu85	BD*(1) N 5- H 29	7.09	0	7.96	15.05
Inactive fGln123	TS4	Val51	BD*(1) N166- H200	0.06	0.05	n/a	0.11
Inactive fGln123	TS4	Gly52	BD*(1) N167- H196	3.70	5.44	n/a	9.14
Inactive fGln123	TS4	Leu85	BD*(1) N 8- H 32	4.89	4.17	n/a	9.06

Inactive Val51	TS2	Val51	BD*(1) C 44- H 64	0	0	n/a	0
Inactive Val51	TS2	Gly52	BD*(1) N 41- H 65	9.16	1.66	n/a	10.82
Inactive Val51	TS2	Leu85	BD*(1) N 69- H 84	4.49	0.48	n/a	4.97
Inactive Val51	TS3	Gly50	BD*(2) C232- O233	0.23	0.11	n/a	0.34
Inactive Val51	TS3	Val51	BD*(1) C212- H140	0	0	0	0
Inactive Val51	TS3	Gly52	BD*(1) N117- H141	7.31	4.97	9.82	22.1
Inactive Val51	TS3	Leu85	BD*(1) N 5- H 30	7.20	2.34	9.63	19.17
Inactive Val51	TS4	Val51	BD*(1) C205- H225	0	0	n/a	0
Inactive Val51	TS4	Gly52	BD*(1) N202- H226	4.14	5.18	n/a	9.32
Inactive Val51	TS4	Leu85	BD*(1) N 44- H 68	4.68	4.45	n/a	9.13
Inactive	TS2	Val51	BD*(1) C 23- H167	5.86	0.15	n/a	6.01
Inactive	TS2	Gly52	BD*(1) N 29- H175	0.08	0.34	n/a	0.42
Inactive	TS2	Leu85	BD*(1) N 39- H156	3.72	0.53	n/a	4.25
Inactive	TS3	Val51	BD*(1) C 23- H167	5.49	0.24	0.92	6.65
Inactive	TS3	Gly52	BD*(1) N 29- H175	0	0.21	0.33	0.54
Inactive	TS3	Leu85	BD*(1) N 39- H156	2.88	0.81	4.11	7.80
Inactive	TS4	Val51	BD*(1) C 23- H167	4.92	0.74	n/a	5.66
Inactive	TS4	Gly52	BD*(1) N 29- H175	0	0.43	n/a	0.43
Inactive	TS4	Leu85	BD*(1) N 39- H156	0	0	n/a	0

Decomposition of *f*Gln123 interaction energy

The proximity of *f*Gln123 to the HisH active site is another structural feature influenced by the binding of the allosteric effector, PrFAR. It is expected that *f*Gln123 assists in recruiting and stabilizing the glutamine substrate, but it is unclear to what extent, if any, this proximity influences the chemical coordinate. The Inactive *f*Gln123 oxyanion hole contributions (Figure 5, SI Figures S4-S5) are nearly identical to Active, with an average difference of less than 0.2 kcal/mol per residue in each TS (Table S4). Therefore, the oxyanion hole stabilization is not influenced by the proximity of *f*Gln123.

To better understand how *f*Gln123 influences the pathway energetics, we evaluated the interaction energy between the *f*Gln123 molecular fragment and the rest of the active site residues for the Active, Inactive *f*Gln123, Inactive Val51 and Inactive models. The different positions of *f*Gln123 are displayed in Figure S8.

Absolutely Localized Molecular Orbitals based Energy Decomposition Analysis[11] (ALMO–EDA) implemented in Q-Chem 5.4[12] was performed at the B3LYP-D3(BJ)/6-311+G(2d,2p) level of theory and CPCM with ε=4.24, to be consistent with the single-point energies used to produce the reaction profiles. Within this framework, the interaction energy is defined as:

$$\Delta E_{INT} = \Delta E_{FRZ} + \Delta E_{POL} + \Delta E_{CT}.$$

E_{POL} and E_{CT} refer to the polarization and charge transfer energy components, respectively. The E_{FRZ} term combines the interaction energy contributions of the unrelaxed fragment densities, namely attractive dispersion (E_{DISP}), repulsive Pauli (E_{PAULI}) and permanent electrostatics (E_{ELEC}):

$$\Delta E_{FRZ} = \Delta\ E_{DISP} + \Delta E_{PAULI} + \Delta E_{ELEC}.$$

The *f*Gln123 interaction energy results are summarized in Figure S7 and reported in Table S5. The total interaction energy (Total INT) in **ES** is much more favorable when *f*Gln123 is closer to the active site. Active- and Inactive Val51-**ES** have similar *f*Gln123 interaction energies of –29.2 and –30.6 kcal/mol, which are much lower than the Inactive *f*Gln123 and Inactive values (–5.4 and –13.1 kcal/mol, respectively). The difference in *f*Gln123 interaction energy between Inactive *f*Gln123 and Inactive can be reasoned by the presence of a hydrogen bond between the sidechain NH_2 group of *f*Gln123 and the backbone carbonyl oxygen of Val140 (Figure S9) that is present in the Inactive model but absent in the Inactive *f*Gln123model.

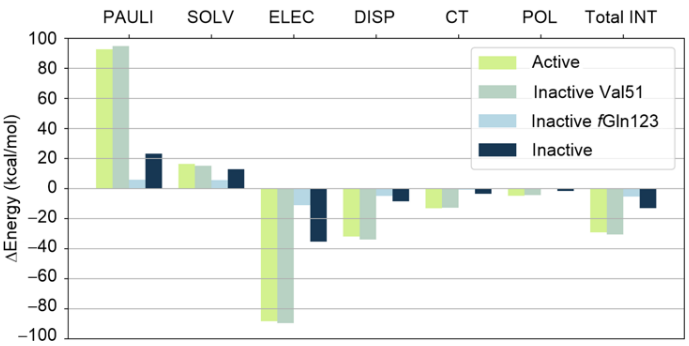

Figure S7. Decomposition of interaction energy calculated with ALMO-EDA between *f*Gln123 and the HisH active site in the optimized **ES** complex for each QM model.

The interaction energy in Inactive Val51 becomes slightly less favorable in **TS2**, **TS3**, and **TS4** because the Gly52 backbone shifts away from *f*Gln123 to interact more strongly with the Gln Oε. The Inactive model also shows interaction energy changes between **ES** and **TS2**, **TS3**, and **TS4**, because the Gln substrate moves farther from *f*Gln123 to react with Cys84. The Active and Inactive *f*Gln123 models have minimal structural rearrangements along the reaction coordinate; therefore, the *f*Gln123 interaction energy values are consistent across the evaluated structures.

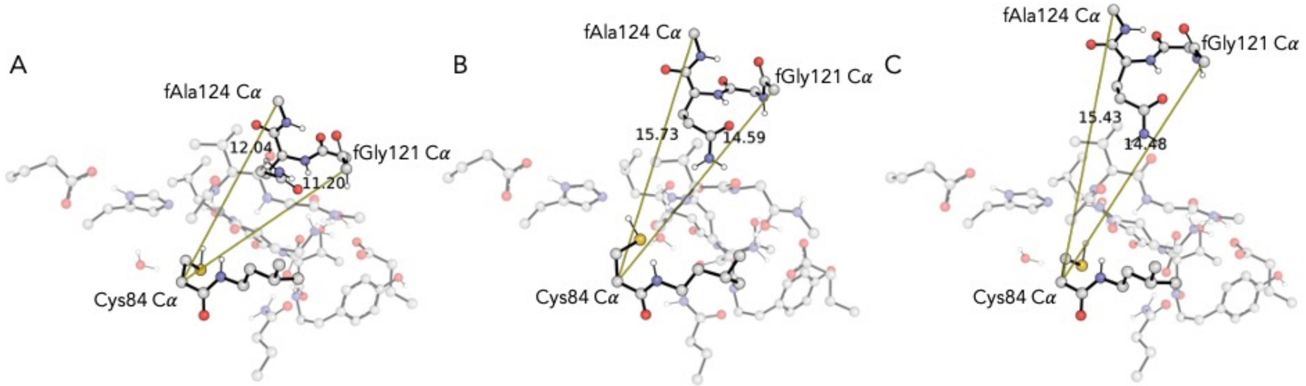

Figure S8. Distances from the frozen Cys84 Cα atom to the frozen *f*Gly121 and *f*Ala124 Cα atoms in A) Active, B) Inactive and C) Inactive *f*Gln123. These distances cannot change during geometry optimizations.

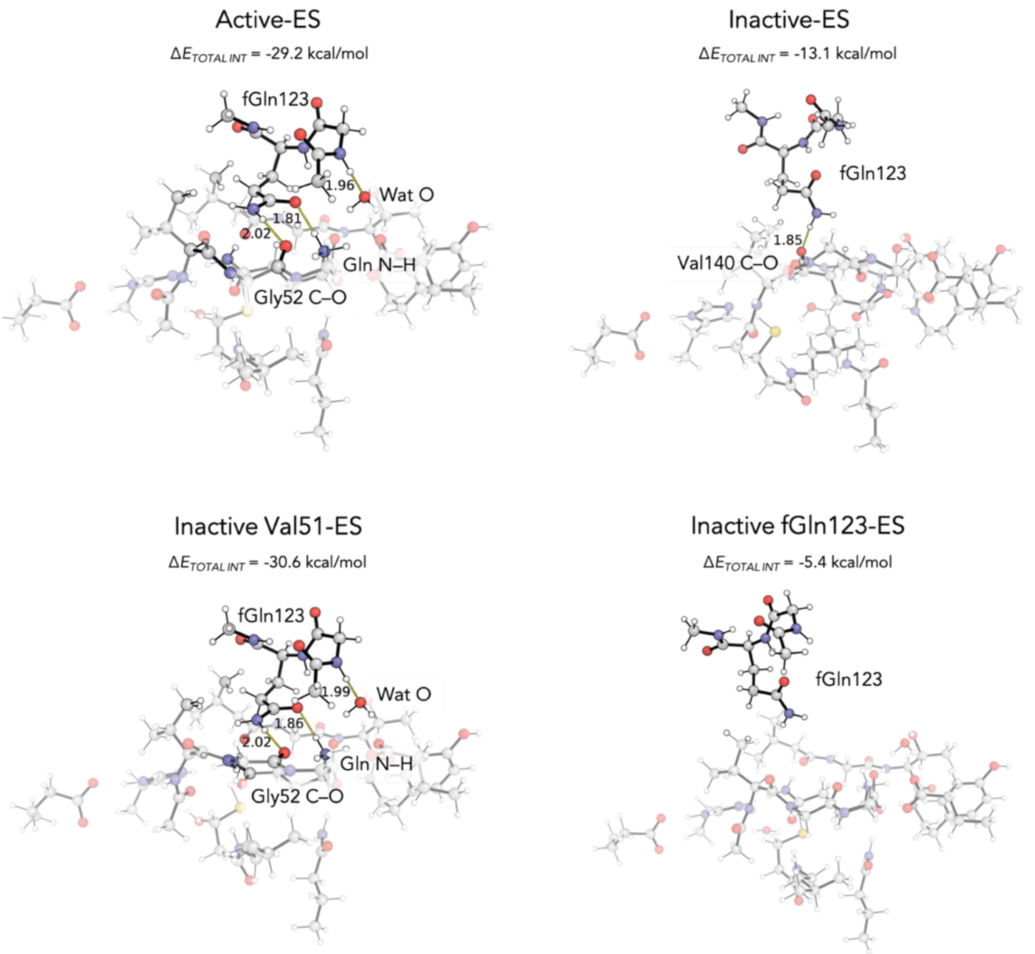

Figure S9. Differences of *f*Gln123 interactions in the **ES** structure of each active site model that effect the *f*Gln123 total interaction energy. Any atom that is not transparent is within 3 Å of an atom in the *f*Gln123 molecular fragment, and it labeled.

The hydrogen bond to Val140 is not present in crystal structures (*f*Gln123:N—*h*Val140:O distance of 5.3 Å in 7ac8 chains C/D) because of a change in the *f*Gln123 rotamer state during geometry optimization. The optimized rotamer state in the Inactive model is structurally feasible due to the open dimer interface in the inactive conformation crystal structure, however it cannot be ruled out that it may be a consequence of the truncated model used here. In the Inactive model, *f*Gln123 forms a single hydrogen bond (1.85 Å) with its NH_2 group and the backbone carbonyl oxygen of Val140. In contrast, *f*Gln123 in the Inactive *f*Gln123 model does not form hydrogen bonds with other active site residues in any of the optimized structures. The presence of this hydrogen bond is likely the reason why the *f*Gln123 interaction energy is more favorable in the Inactive model than the Inactive *f*Gln123 construct, as

opposed to the difference in oxyanion strand conformation. The models with *f*Gln123 in close proximity (Active and Inactive Val51) are highly consistent across all interaction energy terms, indicating the conformation of the oxyanion strand (and subsequently the orientation of the oxyanion hole) does not affect interactions with *f*Gln123. The absence of that hydrogen bond in the Inactive *f*Gln123 construct results from differences in the relative positioning of *f*Gln123, which was based on the Active model rather than the Inactive model.

Table S5. *f*Gln123 interaction energies for **ES** structures measured with ALMO-EDA.

**Component**	**Active**	**Inactive Val51**	**Inactive *f*Gln123**	**Inactive**
**ES**				
ΔELEC	–88.4	–89.5	–11.1	–35.4
ΔSOLV	16.3	15.2	5.6	12.9
ΔPAULI	92.7	94.8	5.9	23.2
ΔDISP	–31.9	–33.9	–4.9	–0.6
ΔPOL	–4.8	–4.4	–0.2	–1.7
ΔCT	–13.1	–12.7	–0.7	–3.5
ΔTotal INT	–29.2	–30.6	–5.4	–13.1
**TS2**				
ΔELEC	–86.6	–73.0	–9.9	–31.0
ΔSOLV	15.1	14.9	5.1	11.3
ΔPAULI	91.4	77.1	4.9	20.7
ΔDISP	–31.6	–31.7	–4.6	–8.0
ΔPOL	–4.5	–3.0	–0.1	–1.2
ΔCT	–12.8	–9.6	–0.7	–3.0
ΔTotal INT	–29.0	–25.3	–5.3	–11.2
**TS3**				
ΔELEC	–86.3	–72.1	–9.5	–30.3
ΔSOLV	15.3	14.7	4.5	10.8
ΔPAULI	90.6	75.8	5.6	20.5
ΔDISP	–31.4	–31.8	–4.7	–7.9
ΔPOL	–4.4	–2.9	–0.1	–1.2
ΔCT	–12.6	–9.6	–0.7	–3.0
ΔTotal INT	–28.8	–5.8	–5.0	–11.1
**TS4**				
ΔELEC	–87.2	–76.4	–9.3	–31.9
ΔSOLV	15.3	14.5	4.3	11.3
ΔPAULI	92.1	81.0	5.7	21.5
ΔDISP	–32.2	–33.5	–4.9	–7.5
ΔPOL	–4.5	–3.0	–0.2	–1.5
ΔCT	–12.7	–10.1	–0.7	–3.2
ΔTotal INT	–29.1	–27.5	–5.1	–11.4

Absolute energies for all evaluated structures

Table S6. Electronic energies at the geometry optimization, ωB97X-D/6-31G*(C,H,N);6-31+G*(S,O), and single point correction, B3LYP-D3(BJ)/6-311+G(2d,2p), levels of theory of each model evaluated.

Model	Structure	Geometry Optimization (kcal/mol)	Single Point Correction (kcal/mol)	Relative to ES Structure (kcal/mol)
**Inactive**	**ES**	-3580984.06	-3583477.89	0.00
	**TS1**	-3580981.05	-3583474.72	3.17
	**Int1**	-3580987.97	-3583480.76	-2.87
	**TS2**	-3580960.80	-3583454.67	23.22
	**Int2**	-3580961.64	-3583452.54	25.35
	**TS3**	-3580956.06	-3583447.39	30.51
	**Int3**	-3580962.05	-3583453.31	24.58
	**TS4**	-3580955.91	-3583451.29	26.60
	**Int4**	-3580959.27	-3583456.61	21.28
**Active**	**ES**	-3581009.49	-3583496.16	0.00
	**TS1**	-3580993.41	-3583481.64	14.52
	**Int1**	-3581008.53	-3583495.28	0.88
	**TS2**	-3580993.62	-3583481.67	14.49
	**Int2**	-3581002.68	-3583484.74	11.42
	**TS3**	-3580995.88	-3583479.46	16.70
	**Int3**	-3580997.45	-3583482.41	13.75
	**TS4**	-3580989.80	-3583477.49	18.67
	**Int4**	-3581003.51	-3583491.35	4.81
**Inactive *f*Gln123**	**ES**	-3580977.63	-3583472.08	0.00
	**TS2**	-3580963.53	-3583459.52	12.56
	**TS3**	-3580962.40	-3583454.44	17.64
	**TS4**	-3580957.53	-3583454.32	17.76
**Inactive Val51**	**ES**	-3581001.97	-3583490.92	0.00
	**TS2**	-3580991.34	-3583481.09	9.83
	**TS3**	-3580988.46	-3583474.47	16.45
	**TS4**	-3580977.34	-3583471.30	19.62

Visual representations of each transition state structure

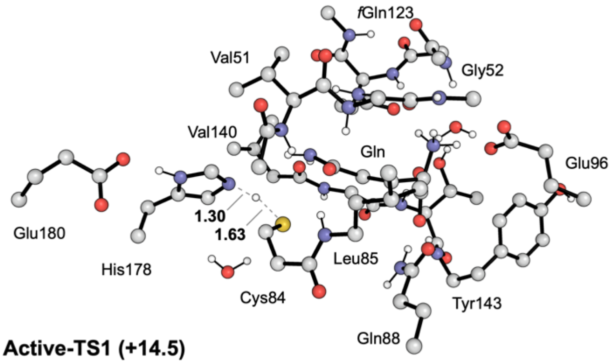

Active-TS1 (+14.5)

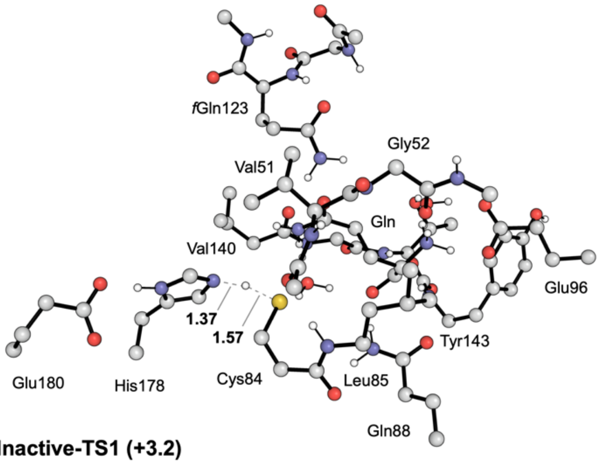

Inactive-TS1 (+3.2)

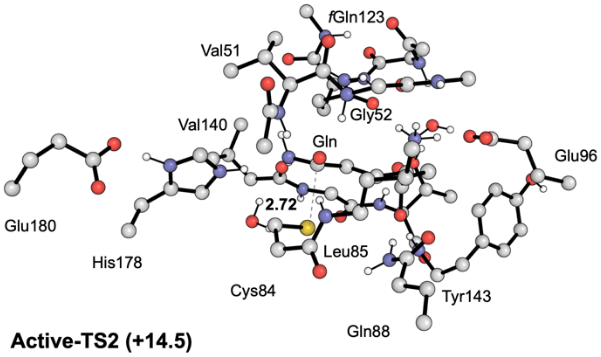

Active-TS2 (+14.5)

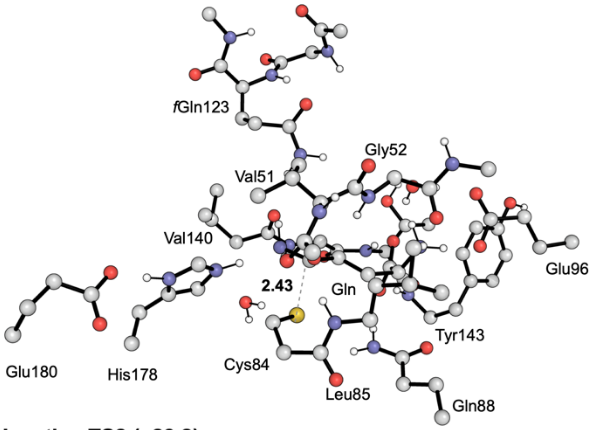

Inactive-TS2 (+23.2)

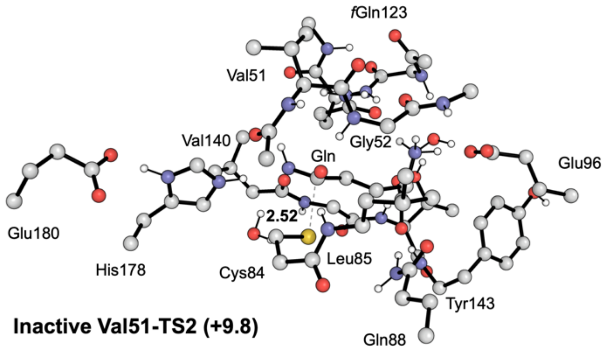

Inactive Val51-TS2 (+9.8)

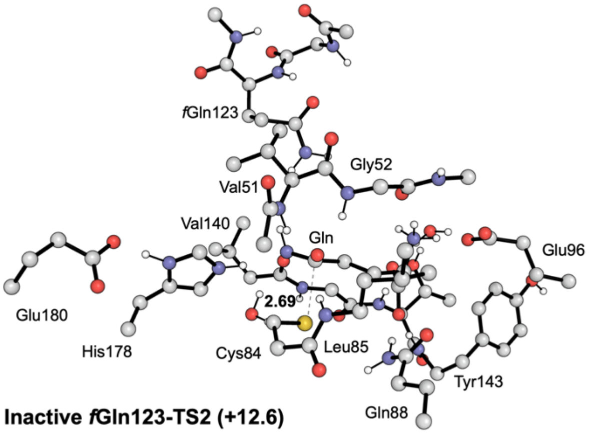

Inactive *f*Gln123-TS2 (+12.6)

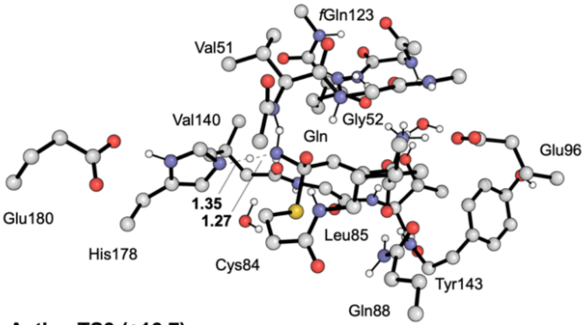

Active-TS3 (+16.7)

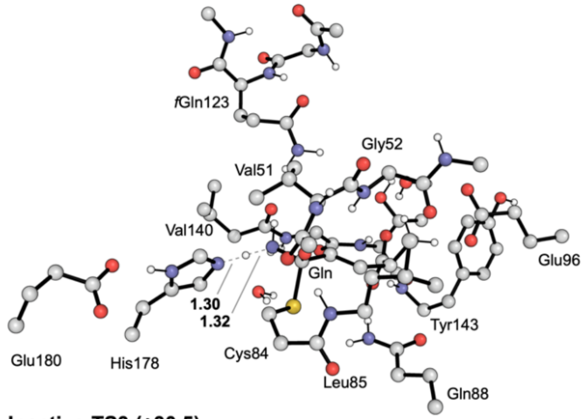

Inactive-TS3 (+30.5)

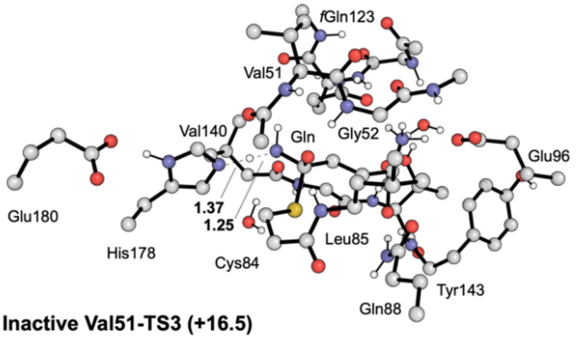

Inactive Val51-TS3 (+16.5)

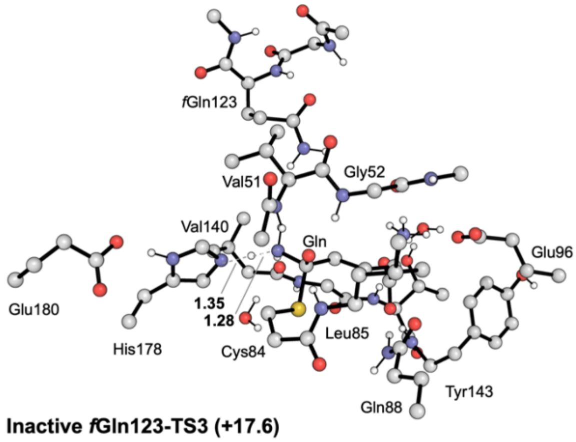

Inactive *f*Gln123-TS3 (+17.6)

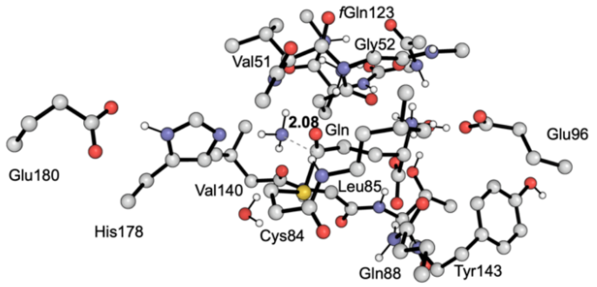

Active-TS4 (+18.7)

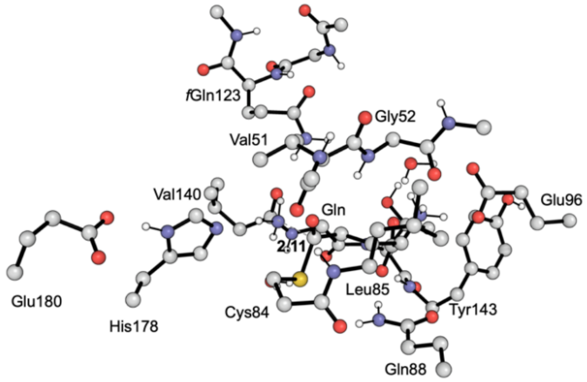

Inactive-TS4 (+26.6)

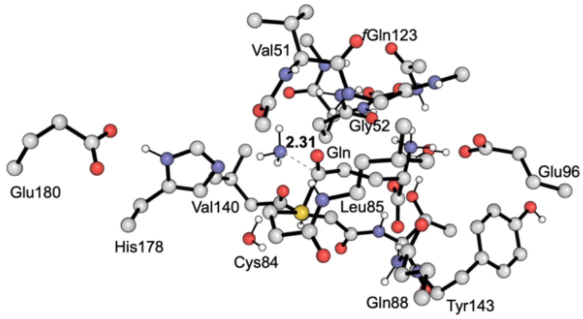

Inactive Val51-TS4 (+19.6)

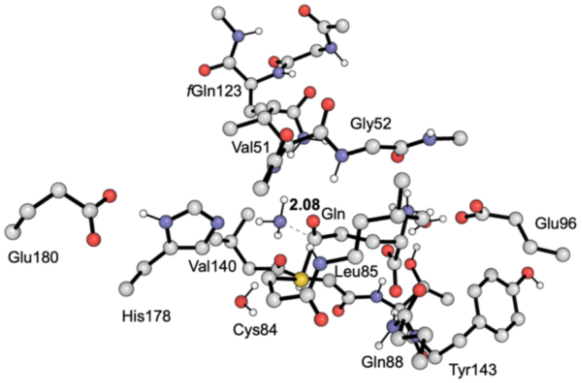

Inactive *f*Gln123-TS4 (+17.8)

Structural comparison with PLP synthase

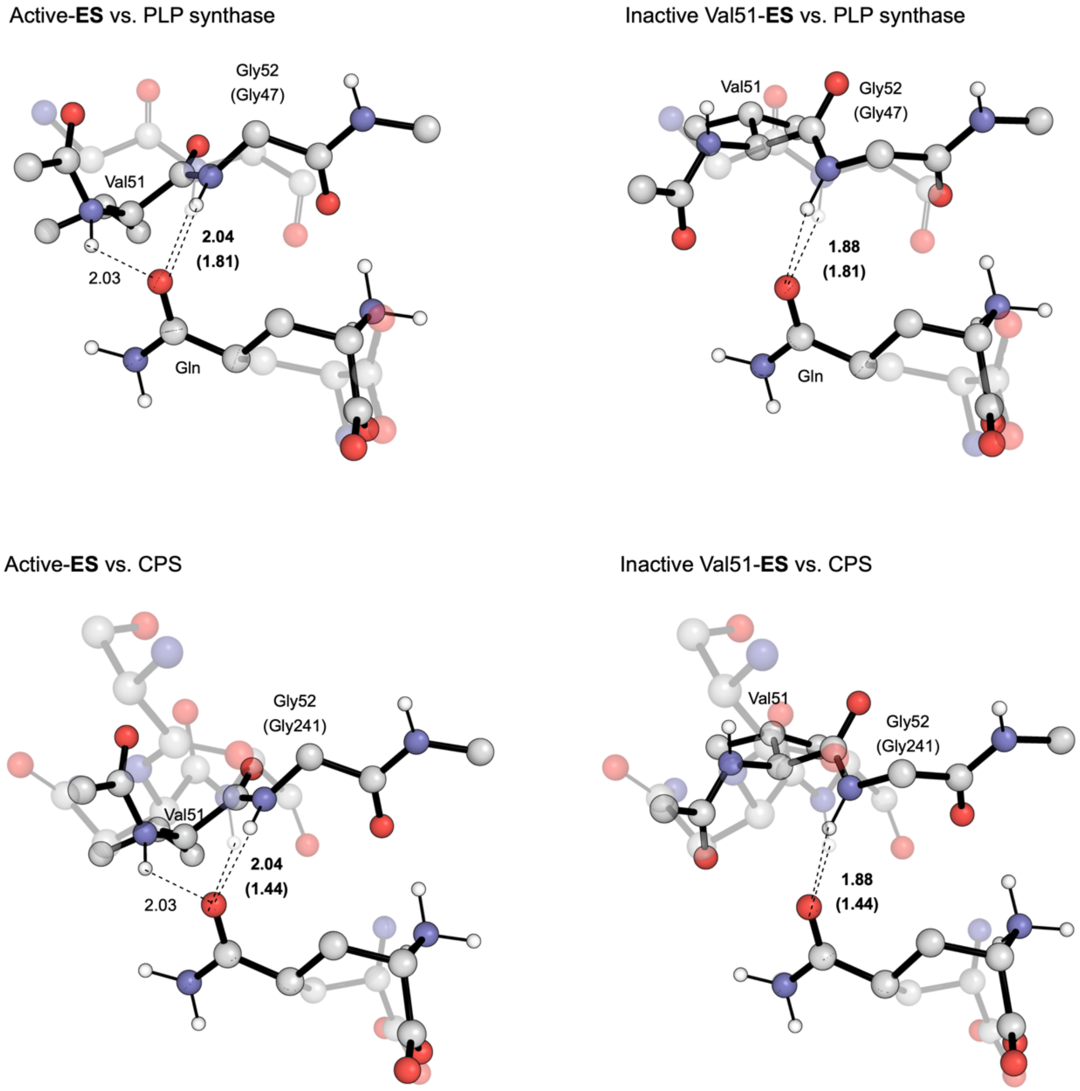

Figure S10. Optimized IGPS geometries of Active-**ES** (left) and Inactive Val51-**ES** (right) models aligned to crystallographic models of PLP synthase (PDB: 2NV2,[14] transparent) and CPS (PDB: 1C3O,[15] transparent) via Gln substrate carboxamide atoms. The preformed oxyanion holes from PLP and CPS are more structurally consistent with the IGPS Gly52 than with Val51, especially in the manually constructed Inactive Val51 model. Interestingly, the backbone dihedral of Gly47 in PLP synthase rotates upon dimer complexation and Gln binding, similar to what is proposed for Val51 in IGPS upon PrFAR binding. The H atoms of PLP synthase Gly47 and CPS Gly241 were added with the PyMol add_hydrogen function since they are not resolved in the crystal structures. Hydrogen bond distances are shown in Å and are in parentheses for the non-optimized structures.

XYZ coordinates of optimized stationary points

237
active_int1
C 3.239367 2.612442 4.156946
C 3.525325 3.997915 3.629575
O 2.917520 4.986066 4.055649
N 4.504883 4.107501 2.698140
C 4.879313 5.406221 2.183593
C 3.817368 6.105091 1.332560
O 3.880839 7.305955 1.092721
N 2.842931 5.288397 0.881145
C 1.753955 5.720546 0.038889
C 0.610722 6.412734 0.792396
O -0.283708 6.975989 0.160847
C 1.177573 4.501292 -0.703401
C 0.247112 3.607765 0.123597
C 0.906408 2.787108 1.217905
N 0.110520 2.317092 2.193174
O 2.117004 2.516601 1.190751
N 0.580315 6.263882 2.131324
C -0.525157 6.800764 2.899386
C -5.231657 -2.385745 2.496524
C -4.962255 -1.137234 3.322485
O -5.661006 -0.833354 4.286558
N -3.927788 -0.385725 2.874581
C -3.459465 0.871792 3.437542
C -2.609659 0.637716 4.708305
O -2.923840 1.031687 5.825057
C -4.516978 1.969029 3.639780
C -3.813805 3.319878 3.821132
C -5.477591 2.030425 2.453490
N -1.437903 -0.010102 4.476562
C -0.517566 -0.238412 5.554114
C 0.891205 -0.491430 5.037174
O 1.268637 -0.039677 3.949313
N 1.679948 -1.182876 5.875028
C 3.092511 -1.467666 5.658504
C -3.820475 -3.833852 -1.682532
C -2.861336 -4.660814 -0.826063
O -2.571023 -5.820085 -1.145423
C -4.669665 -2.764552 -1.001582
N -2.357462 -4.063779 0.272543
C -1.442810 -4.715496 1.193090
C -1.522948 -4.009995 2.548123
C -0.489386 -4.473134 3.585141
C 0.919492 -3.955059 3.276754
C -0.930095 -4.012498 4.977980
C 0.644453 -8.633250 -0.954201
C 1.296895 -7.331986 -0.493622
C 0.930677 -6.165326 -1.406708
C 1.657966 -4.881438 -1.041711
N 1.776043 -3.971542 -2.030600
O 2.101118 -4.676216 0.091460
C 8.884343 -3.342576 1.835642
C 7.480905 -2.788496 2.086557
C 7.499009 -1.373468 2.687403
C 6.095692 -0.768915 2.699090
O 5.393017 -0.860937 3.723225
O 5.698578 -0.240056 1.598722
C -2.034866 3.639425 -4.440863
C -1.124414 2.738801 -3.615517
O -1.577702 2.028340 -2.700623
C -2.846870 4.655703 -3.607372
C -3.982274 4.012449 -2.805566
C -1.942127 5.490933 -2.699682
N 0.182492 2.734409 -3.915023
C 1.127922 1.818967 -3.312676
C 2.419841 1.828330 -4.147257
O 2.436338 2.375445 -5.251059
N 3.438404 1.181827 -3.565800
C 4.790648 1.136139 -4.091356
C 5.160447 -0.217794 -4.732609
O 5.901791 -0.258155 -5.714229
C 5.774711 1.441918 -2.932551
C 7.231833 1.415506 -3.375749
O 5.437386 2.710840 -2.398449
N 4.689679 -1.316011 -4.099579
C 5.158909 -2.659711 -4.388597
C 5.732599 -3.330306 -3.130272
C 6.882328 -2.552081 -2.538285
C 8.147870 -2.583047 -3.124902
C 6.702066 -1.734378 -1.420106
C 9.202014 -1.832304 -2.612566
C 7.739259 -0.969420 -0.901061
C 8.993421 -1.022058 -1.498579
O 9.994981 -0.257134 -0.953497
C -8.895646 -0.538666 -3.810084
C -9.107813 0.554139 -2.756909
C -8.193586 0.349204 -1.591435
C -6.826925 0.357470 -1.539420
N -8.640718 -0.009105 -0.340187
C -7.590532 -0.224249 0.440292
N -6.476659 0.002455 -0.259453
C -13.971699 -0.147882 -2.003423
C -13.961203 -1.547020 -1.389026
C -13.081188 -1.620739 -0.139771
C -11.619840 -1.271338 -0.448476
O -11.074905 -1.798849 -1.434053
O -11.065251 -0.434797 0.347929
N 3.001892 -0.212113 1.657115
C 2.608297 -1.198883 0.616587
C 3.082929 -0.679274 -0.745685
O 3.352201 0.545927 -0.826508
C 1.092049 -1.429409 0.601408
C 0.290979 -0.344426 -0.127176
C -0.996521 0.013520 0.580193
N -1.748821 0.971567 -0.007606
O -1.308753 -0.483004 1.665884
O 3.091925 -1.496608 -1.696911
O -4.024484 0.530570 -3.166850
O 5.158409 2.237023 0.494264
H -7.857362 -0.553647 -4.158760
H -0.446180 -8.529975 -0.985885
H 8.840888 -4.363250 1.439262
H 3.543894 2.577255 5.208224
H 5.775762 5.282485 1.569832
H -2.721385 2.978158 -4.984246
H 0.714102 0.803629 -3.296725
H 4.335430 -3.269293 -4.783563
H -12.963806 0.135490 -2.324216
H -14.315502 0.599227 -1.277295
H -9.547240 -0.371271 -4.675253
H -4.466841 -4.573947 -2.168622
H -1.727908 -5.768963 1.277707
H 0.889618 -9.461657 -0.279688
H 9.476918 -3.361163 2.759207
H 3.619123 -1.413845 6.613911
H 3.230782 -2.460827 5.219823
H -5.954623 -2.154841 1.708439
H -0.381709 6.526952 3.946107
H -0.576728 7.890934 2.809622
H -14.630207 -0.098356 -2.879083
H -9.143890 -1.512009 -3.378483
H 0.982097 -8.909787 -1.960857
H 5.919210 -2.573290 -5.169075
H -1.473228 6.386503 2.541770
H 3.530528 -0.740947 4.973506
H 9.414567 -2.720114 1.105246
H -0.415422 -4.687177 0.807153
H -3.226155 -3.366902 -2.478780
H -1.437358 4.177652 -5.185966
H 1.345146 2.094539 -2.275001
H 5.114090 6.084412 3.007599
H 2.165561 2.428321 4.114230
H 3.769567 1.822927 3.617876
H -14.986553 -1.847928 -1.132523
H -13.583246 -2.264108 -2.125470
H -13.453123 -0.947044 0.639566
H -13.100277 -2.640862 0.265393
H -8.929900 1.544177 -3.194211
H -10.142244 0.522460 -2.403995
H -9.702565 -0.192362 -0.053742
H -6.069395 0.567212 -2.285535
H -7.634426 -0.530009 1.474137
H -4.334561 -2.775944 2.011582
H -5.671533 -3.142387 3.149791
H -2.518373 -3.058444 0.363833
H -1.404787 -2.926510 2.396822
H -2.534540 -4.159125 2.949897
H -0.460708 -5.573078 3.578568
H -0.168312 -4.236954 5.735259
H -1.102061 -2.928648 4.972354
H -1.868800 -4.488101 5.283683
H 1.645611 -4.358427 3.993701
H 1.259611 -4.224507 2.271677
H 0.944583 -2.860289 3.362953
H 2.387238 -7.445806 -0.461590
H 0.985459 -7.091521 0.528294
H 1.148590 -6.420257 -2.452944
H -0.152678 -5.980330 -1.351552
H 6.934030 -2.767180 1.138128
H 6.925260 -3.453585 2.759412
H 7.885886 -1.409662 3.711437
H 8.161279 -0.736850 2.089355
H 6.055347 -4.343841 -3.402471
H 4.934241 -3.437138 -2.386985
H 4.081580 -1.207196 -3.285128
H 1.358698 -4.135711 -2.932363
H 2.243424 -3.076045 -1.853258
H 5.591348 1.425014 0.834998
H 4.316549 1.882707 0.146061
H -3.864501 -0.140153 -2.465675
H -3.262783 1.128164 -3.077324
H -3.588711 3.375301 -2.008598
H -4.602396 4.790145 -2.344981
H -1.094165 5.921912 -3.244781
H -2.492525 6.314332 -2.234749
H -3.303746 5.328254 -4.346952
H 0.538102 3.209955 -4.738239
H 3.340133 0.940689 -2.572224
H 0.455861 1.594728 2.824317
H -0.890249 2.417370 2.113075
H 4.890514 1.895409 -4.870543
H 5.622581 0.673450 -2.162936
H 7.531549 0.422656 -3.723261
H 7.395076 2.135019 -4.184979
H 7.864337 1.690154 -2.525536
H 8.316894 -3.201337 -4.003886
H 10.181432 -1.872723 -3.086403
H 7.558980 -0.334934 -0.040122
H 5.728549 -1.678928 -0.940464
H 10.772395 -0.303579 -1.521739
H 5.498463 2.656969 -1.427775
H 4.795653 3.290217 2.158072
H 2.925243 4.292422 1.058047
H 1.384501 5.876055 2.615864
H 4.066134 -0.179236 1.725820
H 2.681575 0.740615 1.415573
H 2.610226 -0.436166 2.580585
H 3.109928 -2.141199 0.849753
H 0.908379 -2.405052 0.146472
H 0.738085 -1.500612 1.633327
H 0.872116 0.580108 -0.238982
H 0.040961 -0.657785 -1.147396
H -1.597422 1.192198 -0.991068
H -2.720901 1.002102 0.275927
H -1.544051 4.878678 -1.883134
H -4.623569 3.387838 -3.436392
H -6.090329 1.129753 2.391641
H -6.153820 2.886244 2.554860
H -4.939754 2.144382 1.502076
H -3.259877 3.596619 2.911537
H -4.548898 4.110773 4.005376
H -3.118061 3.307515 4.664549
H -5.080952 1.734801 4.546261
H -2.758442 1.241450 2.679355
H -1.211141 -0.321617 3.534049
H -0.845435 -1.066532 6.197865
H -0.490915 0.654077 6.192903
H 1.236595 -1.591303 6.683835
H -3.416151 -0.727226 2.064578
H 0.604981 4.866512 -1.557969
H 2.014708 3.914648 -1.097329
H 2.129472 6.446574 -0.688209
H -0.216196 2.879458 -0.552755
H -0.585293 4.187046 0.538467
S -3.688241 -1.302557 -0.517202
H -5.495071 -0.258879 -0.018929
H -5.455571 -2.456849 -1.700886

237
active_int2
C 3.193217 2.596243 4.236793
C 3.367241 3.934710 3.555645
O 2.765759 4.943575 3.943300

N 4.262132 3.984186 2.538832
C 4.593905 5.241755 1.906549
C 3.472869 5.889357 1.093077
O 3.505410 7.079797 0.799435
N 2.479897 5.047344 0.739670
C 1.312243 5.458565 -0.003095
C 0.290896 6.242099 0.826989
O -0.641647 6.819637 0.267435
C 0.606170 4.219254 -0.580830
C -0.245435 3.415910 0.407756
C 0.532351 2.655898 1.465780
N -0.139856 2.252938 2.555882
O 1.715742 2.335001 1.286918
N 0.395086 6.146826 2.167002
C -0.595027 6.773872 3.013640
C -5.242244 -2.437621 2.500946
C -4.832054 -1.203641 3.284149
O -5.179830 -1.006696 4.444498
N -4.064491 -0.334919 2.568351
C -3.329528 0.753914 3.196517
C -2.283171 0.229709 4.211315
O -2.165522 0.681197 5.344246
C -4.194005 1.886253 3.780957
C -3.366672 3.172788 3.886013
C -5.441776 2.129911 2.931974
N -1.480650 -0.760859 3.733506
C -0.616288 -1.474823 4.629173
C 0.837238 -0.985825 4.692227
O 1.231504 0.012981 4.082114
N 1.638697 -1.739320 5.468915
C 3.068375 -1.499727 5.696047
C -3.809242 -3.832981 -1.688881
C -2.926851 -4.689084 -0.765874
O -2.664695 -5.850231 -1.094530
C -3.658541 -2.322555 -1.555025
N -2.504845 -4.132079 0.396022
C -1.435435 -4.729286 1.184159
C -1.683264 -4.653355 2.684029
C -0.599658 -5.387681 3.491827
C 0.812497 -4.835944 3.252460
C -0.946181 -5.398294 4.984213
C 8.881928 -3.297886 1.868152
C 7.374286 -3.052687 1.931924
C 7.029732 -1.705080 2.581452
C 5.541711 -1.380578 2.448842
O 4.717598 -2.099713 3.045615
O 5.219338 -0.390280 1.701786
C -2.066570 3.680456 -4.364397
C -1.125789 2.805451 -3.551532
O -1.527839 2.161021 -2.565748
C -3.159039 4.427659 -3.573776
C -4.286157 3.514079 -3.081945
C -2.583647 5.258244 -2.424521
N 0.138157 2.701907 -3.974360
C 1.070792 1.746434 -3.415610
C 2.384675 1.829169 -4.203739
O 2.413954 2.379888 -5.306686
N 3.410420 1.234178 -3.583893
C 4.777772 1.216373 -4.060064
C 5.195265 -0.131209 -4.683690
O 6.028946 -0.165642 -5.588714
C 5.707341 1.523899 -2.856447
C 7.184306 1.534712 -3.228452
O 5.326642 2.771846 -2.304778
N 4.662474 -1.234946 -4.111053
C 5.168643 -2.574506 -4.356975
C 5.625782 -3.237140 -3.046886
C 6.731375 -2.460254 -2.375479
C 8.043694 -2.537542 -2.843623
C 6.467783 -1.591436 -1.313743
C 9.062974 -1.780800 -2.274092
C 7.470687 -0.817138 -0.742790
C 8.771140 -0.914548 -1.223063
O 9.733800 -0.138451 -0.626321
C -8.900950 -0.548155 -3.794792
C -8.155363 0.737862 -3.410444
C -7.197074 0.572984 -2.271464
C -5.832016 0.500460 -2.261640
N -7.612309 0.486878 -0.958654
C -6.539303 0.377979 -0.182101
N -5.442867 0.384796 -0.945447
C -13.977628 -0.139263 -2.012368
C -13.580025 -0.339109 -0.547850
C -12.397002 0.522721 -0.102086
C -11.009560 0.064769 -0.587957
O -10.883795 -1.023423 -1.171854
O -10.062978 0.883714 -0.308683
N 2.519685 -0.394726 1.500250
C 2.136405 -1.296252 0.383463
C 2.655194 -0.672136 -0.920062
O 2.996811 0.539250 -0.887280
C 0.626588 -1.569991 0.328646
C -0.256530 -0.378273 -0.040884
C -1.758438 -0.730568 -0.130885
N -2.597764 0.470523 -0.379874
O -2.200570 -1.391161 0.926345
O 2.675930 -1.401372 -1.940491
O -3.214661 0.364721 -4.005693
O 4.886700 2.030916 0.474692
H -8.191948 -1.329767 -4.090286
H 9.106816 -4.272728 1.420249
H 2.147444 2.458815 4.511902
H 5.440767 5.068339 1.236586
H -2.538762 3.014831 -5.100589
H 0.669997 0.727319 -3.494640
H 4.389647 -3.189894 -4.825185
H -13.139016 -0.394030 -2.668043
H -14.258879 0.904402 -2.204809
H -9.568181 -0.353048 -4.641784
H -4.854069 -4.111126 -1.497004
H -1.345952 -5.775609 0.877047
H 9.332419 -3.272963 2.869020
H 3.359303 -2.034739 6.602525
H 3.669270 -1.832194 4.842576
H -5.945711 -3.023848 3.093043
H -0.356431 6.540256 4.052937
H -0.605238 7.860573 2.876493
H -14.829650 -0.773966 -2.284043
H -9.504526 -0.911623 -2.956072
H 5.998451 -2.480611 -5.061734
H -1.594693 6.395367 2.775853
H 3.223297 -0.428083 5.844525
H 9.370399 -2.526951 1.260541
H -0.492955 -4.232749 0.925928
H -3.577166 -4.162022 -2.705635
H -1.471426 4.405515 -4.933415
H 1.240125 1.937132 -2.352248
H 4.892112 5.975800 2.659399
H 3.515123 1.756486 3.615763
H 3.790074 2.601084 5.155830
H -14.444759 -0.111940 0.091893
H -13.324886 -1.391791 -0.385804
H -12.534310 1.567200 -0.410539
H -12.341866 0.545149 0.994166
H -7.587795 1.118455 -4.267264
H -8.885028 1.509274 -3.137980
H -8.678512 0.564294 -0.649702
H -5.098107 0.509524 -3.059302
H -6.538968 0.308865 0.894216
H -5.708079 -2.161993 1.548315
H -4.363604 -3.049064 2.263977
H -2.554927 -3.113695 0.514185
H -1.746203 -3.599358 2.990384
H -2.663117 -5.097543 2.904323
H -0.602638 -6.433526 3.149248
H -0.206823 -5.971866 5.556024
H -0.971075 -4.381271 5.397062
H -1.932776 -5.841472 5.161802
H 1.541909 -5.358729 3.882795
H 1.141506 -4.942596 2.213948
H 0.878645 -3.768462 3.500142
H 6.967991 -3.078852 0.914977
H 6.874321 -3.851755 2.490308
H 7.281470 -1.746119 3.649208
H 7.624454 -0.906672 2.127192
H 5.959634 -4.257215 -3.277424
H 4.765513 -3.326989 -2.373290
H 3.945886 -1.133303 -3.389247
H 5.292130 1.230350 0.869980
H 4.060407 1.666282 0.093669
H -2.779104 -0.416153 -3.618857
H -2.785770 1.082661 -3.508940
H -3.933401 2.843734 -2.293319
H -5.108878 4.110665 -2.671618
H -1.745836 5.887661 -2.744865
H -3.347029 5.917580 -1.998732
H -3.601104 5.123047 -4.301572
H 0.460841 3.154217 -4.823793
H 3.263604 0.967798 -2.602620
H 0.290952 1.591128 3.203743
H -1.064099 2.598545 2.761320
H 4.897079 1.981939 -4.830393
H 5.535031 0.735809 -2.111722
H 7.521754 0.557329 -3.584042
H 7.371409 2.276641 -4.011923
H 7.767282 1.802914 -2.341482
H 8.277173 -3.198553 -3.675510
H 10.079902 -1.859372 -2.654941
H 7.236863 -0.139422 0.070250
H 5.458705 -1.505357 -0.918365
H 10.558862 -0.226486 -1.117133
H 5.299793 2.666362 -1.336659
H 4.533813 3.132281 2.043318
H 2.568155 4.062261 0.968279
H 1.233429 5.757370 2.589695
H 3.578766 -0.398691 1.621695
H 2.234696 0.581295 1.318882
H 2.114142 -0.664183 2.400712
H 2.644184 -2.251365 0.547425
H 0.475557 -2.375493 -0.394531
H 0.309452 -1.972538 1.298686
H -0.188813 0.381225 0.747430
H 0.079589 0.091306 -0.974458
H -2.236580 0.992983 -1.179442
H -2.483290 1.082696 0.429523
H -2.228823 4.609841 -1.617287
H -4.687790 2.897629 -3.894215
H -6.130280 1.282600 2.989898
H -5.975166 3.017357 3.290070
H -5.183225 2.298859 1.878067
H -3.082846 3.535266 2.885200
H -3.950709 3.971057 4.356258
H -2.467485 3.025001 4.492069
H -4.501283 1.583814 4.785523
H -2.743522 1.184950 2.373118
H -1.594379 -1.076377 2.762585
H -0.607996 -2.529065 4.342448
H -1.035483 -1.405136 5.639006
H 1.247631 -2.596383 5.831768
H -3.564104 -0.744650 1.761199
H -0.050308 4.551882 -1.386087
H 1.366990 3.568749 -1.022313
H 1.618946 6.116325 -0.821596
H -0.775146 2.648864 -0.173840
H -1.015129 4.045008 0.870604
S -1.942441 -1.751122 -1.789784
H -4.442111 0.337192 -0.635717
H -4.314308 -1.828192 -2.276730
H -3.963339 -1.990644 -0.556612
C 0.684052 -8.611113 -1.000635
C 1.259087 -7.268621 -0.562725
C 0.807836 -6.142372 -1.487244
C 1.518790 -4.832661 -1.198988
N 1.602498 -3.973849 -2.237242
O 1.999227 -4.580869 -0.091598
H -0.411492 -8.577271 -1.021976
H 0.985933 -9.416823 -0.321341
H 1.027610 -8.876790 -2.008214
H 2.354421 -7.313743 -0.546522
H 0.950420 -7.035923 0.462291
H 0.980881 -6.423387 -2.535025
H -0.275434 -5.982895 -1.384614
H 1.056698 -4.139903 -3.068310
H 1.979430 -3.030154 -2.088136

237
active_int3
C 0.684720 -8.610678 -1.000548
C 1.256405 -7.262514 -0.576868
C 0.798912 -6.148004 -1.512612
C 1.479188 -4.824891 -1.216238
N 1.613445 -3.987354 -2.265498
O 1.883992 -4.539256 -0.086550
H -0.410967 -8.580617 -1.018373
H 0.991342 -9.408828 -0.314723
H 1.025774 -8.885010 -2.006598
H 2.352094 -7.304031 -0.561964
H 0.947531 -7.019398 0.445456
H 0.989142 -6.431481 -2.556683
H -0.287919 -6.006791 -1.421006
H 1.160336 -4.198827 -3.140208
H 1.983025 -3.039103 -2.124745

C 3.192806 2.596281 4.236875
C 3.387882 3.935511 3.564271
O 2.798356 4.949547 3.955419
N 4.287560 3.978217 2.551270
C 4.639053 5.236632 1.931292
C 3.525669 5.910754 1.129431
O 3.570910 7.105319 0.856841
N 2.524146 5.085828 0.758162
C 1.371288 5.522815 0.006985
C 0.341900 6.297051 0.836615
O -0.575114 6.893742 0.273589
C 0.661325 4.301796 -0.602340
C -0.203330 3.486836 0.365112
C 0.551060 2.685587 1.409392
N -0.148632 2.248768 2.473021
O 1.729213 2.347695 1.235710
N 0.415251 6.164727 2.176845
C -0.595634 6.772713 3.014651
C -3.809795 -3.832763 -1.688923
C -2.904877 -4.669310 -0.779150
O -2.651130 -5.835358 -1.095651
C -3.717588 -2.313423 -1.581839
N -2.458207 -4.097516 0.364290
C -1.434781 -4.729810 1.183086
C -1.720517 -4.634834 2.675728
C -0.683382 -5.397328 3.517383
C 0.750668 -4.888248 3.317134
C -1.073262 -5.389083 4.998864
C 8.882579 -3.297863 1.868454
C 7.371341 -3.062368 1.903397
C 7.002636 -1.721508 2.554557
C 5.510899 -1.413853 2.415219
O 4.691705 -2.133923 3.017076
O 5.180536 -0.432801 1.658195
C -2.065485 3.680699 -4.363251
C -1.137918 2.788215 -3.547658
O -1.469616 2.262300 -2.470217
C -3.133966 4.465765 -3.581154
C -4.271537 3.576220 -3.074180
C -2.539870 5.309804 -2.450908
N 0.078170 2.570627 -4.065894
C 1.036517 1.664776 -3.475941
C 2.347067 1.745161 -4.270936
O 2.359974 2.227057 -5.405246
N 3.393795 1.226652 -3.617212
C 4.760840 1.216701 -4.093281
C 5.189536 -0.131989 -4.706927
O 6.030787 -0.170182 -5.604146
C 5.685370 1.531457 -2.887555
C 7.162763 1.560855 -3.256616
O 5.288080 2.772380 -2.330530
N 4.655032 -1.233037 -4.129942
C 5.167876 -2.572175 -4.358699
C 5.629530 -3.218456 -3.042120
C 6.734810 -2.432799 -2.380450
C 8.045228 -2.507681 -2.854526
C 6.473392 -1.561028 -1.320763
C 9.064790 -1.746021 -2.292383
C 7.476796 -0.782098 -0.756940
C 8.775418 -0.877320 -1.242545
O 9.738036 -0.096832 -0.652362
C -8.900717 -0.548210 -3.794825
C -8.029216 0.684674 -3.508947
C -7.079675 0.529124 -2.360121
C -5.707763 0.537155 -2.300439
N -7.504461 0.393898 -1.061493
C -6.409295 0.336591 -0.281395
N -5.291570 0.421737 -0.990091
C -13.977306 -0.139427 -2.012292
C -13.614336 -0.424763 -0.553713
C -12.399896 0.363681 -0.062091
C -11.031454 -0.096638 -0.612525
O -10.957279 -1.184586 -1.217046
O -10.070301 0.700352 -0.364230
N 2.488725 -0.404949 1.456535
C 2.095967 -1.292763 0.333117
C 2.635712 -0.672632 -0.963498
O 2.992287 0.532768 -0.920752
C 0.581903 -1.538618 0.254128
C -0.264913 -0.339704 -0.174530
C -1.762620 -0.691686 -0.254868
N -2.535810 0.626702 -0.494152
O -2.246470 -1.284333 0.802428
O 2.646112 -1.398946 -1.986472
O -2.926688 0.434675 -4.520535
O 4.883266 2.017790 0.470104
H -8.273236 -1.412456 -4.043590
H 9.121888 -4.268408 1.418902
H 2.143999 2.471437 4.507670
H 5.480452 5.055966 1.256378
H -2.558409 3.008462 -5.077640
H 0.669098 0.629759 -3.508663
H 4.392365 -3.197441 -4.819919
H -13.137653 -0.402507 -2.663061
H -14.206069 0.924446 -2.159786
H -9.562737 -0.350654 -4.646159
H -4.844911 -4.130163 -1.476213
H -1.382871 -5.779961 0.881683
H 9.312459 -3.276815 2.878291
H -0.389065 6.505352 4.052747
H -0.596336 7.863194 2.911681
H -14.853029 -0.719708 -2.328925
H -9.521000 -0.808769 -2.929320
H 5.996819 -2.482779 -5.065143
H -1.589499 6.408185 2.735191
H 9.378261 -2.520071 1.275556
H -0.463927 -4.278735 0.947288
H -3.593826 -4.168070 -2.707558
H -1.448472 4.378225 -4.943522
H 1.203834 1.911375 -2.423178
H 4.952312 5.957434 2.690729
H 3.505400 1.754925 3.612985
H 3.785378 2.587670 5.158527
H -14.478537 -0.190683 0.085567
H -13.404607 -1.493738 -0.440105
H -12.512218 1.432749 -0.283996
H -12.328003 0.293890 1.031712
H -7.436568 0.944799 -4.394837
H -8.685134 1.540472 -3.304853
H -8.523140 0.409673 -0.757729
H -4.996657 0.638909 -3.111404
H -6.453998 0.244226 0.794460
H -2.519996 -3.082118 0.477971
H -1.757332 -3.577824 2.977555
H -2.719987 -5.044219 2.872959
H -0.707936 -6.444038 3.178651
H -0.367767 -5.980314 5.594750
H -1.080273 -4.369341 5.405442
H -2.076896 -5.802413 5.149793
H 1.446986 -5.438481 3.961161
H 1.100892 -4.996617 2.285852
H 0.843858 -3.825307 3.575850
H 6.985843 -3.084557 0.878155
H 6.866693 -3.868711 2.447100
H 7.247640 -1.762968 3.623806
H 7.589908 -0.914139 2.106625
H 5.965013 -4.240414 -3.261950
H 4.770787 -3.302879 -2.365815
H 3.931851 -1.125770 -3.416025
H 5.275357 1.205310 0.854705
H 4.053242 1.671317 0.082621
H -2.533371 0.104726 -3.693817
H -3.107634 -0.361477 -5.032886
H -3.919183 2.880026 -2.309470
H -5.070290 4.184062 -2.634155
H -1.691807 5.916964 -2.788327
H -3.290249 5.992248 -2.038310
H -3.566096 5.157612 -4.318823
H 0.354014 2.950155 -4.965053
H 3.253164 0.991953 -2.628023
H 0.286268 1.600988 3.131939
H -1.037873 2.656687 2.718688
H 4.877940 1.979071 -4.867336
H 5.521003 0.738596 -2.146174
H 7.512607 0.588022 -3.612596
H 7.342746 2.306126 -4.038501
H 7.740781 1.834730 -2.368113
H 8.277015 -3.170892 -3.685087
H 10.080064 -1.822861 -2.677806
H 7.245460 -0.101798 0.054544
H 5.466574 -1.476053 -0.919535
H 10.561746 -0.184035 -1.145638
H 5.285307 2.668690 -1.362299
H 4.550069 3.125741 2.052200
H 2.605081 4.095185 0.961827
H 1.251099 5.778426 2.607008
H 3.550531 -0.427991 1.579262
H 2.225280 0.577746 1.279784
H 2.078227 -0.670443 2.356473
H 2.579170 -2.260118 0.498140
H 0.429688 -2.362210 -0.448222
H 0.233461 -1.901956 1.228415
H -0.182502 0.450158 0.581221
H 0.086725 0.078152 -1.125721
H -2.180615 1.165727 -1.303851
H -2.426378 1.211244 0.337808
H -2.194305 4.673060 -1.630718
H -4.704033 2.980510 -3.884696
H 0.007480 4.655968 -1.400806
H 1.416098 3.652573 -1.057500
H 1.695148 6.193064 -0.794426
H -0.732388 2.748061 -0.250615
H -0.971090 4.112468 0.835512
S -2.043751 -1.644016 -1.900933
H -3.589483 0.466388 -0.629339
H -4.418706 -1.852787 -2.280634
H -4.008036 -1.969585 -0.585045
C -5.241600 -2.437071 2.500815
C -4.782757 -1.237203 3.309925
O -5.047107 -1.084771 4.498060
N -4.059390 -0.341082 2.584287
C -3.323134 0.757762 3.184942
C -2.267466 0.255631 4.198842
O -2.125056 0.735019 5.315784
C -4.189788 1.897834 3.751469
C -3.365578 3.188440 3.829158
C -5.440784 2.117966 2.900174
N -1.478738 -0.754344 3.734573
C -0.617053 -1.463868 4.635912
C 0.841120 -0.986702 4.681854
O 1.241280 -0.011121 4.038704
N 1.635691 -1.724975 5.478973
C 3.069128 -1.498626 5.695419
H 3.362824 -2.042774 6.595227
H 3.658831 -1.832624 4.834539
H -5.912678 -3.045750 3.107589
H 3.236876 -0.429558 5.849623
H -5.759816 -2.117986 1.590041
H -4.379890 -3.041962 2.193835
H -6.125819 1.269516 2.971124
H -5.976719 3.010149 3.242074
H -5.190103 2.262586 1.841198
H -3.087770 3.531585 2.819577
H -3.951125 3.994328 4.283713
H -2.463865 3.056458 4.435593
H -4.490166 1.612918 4.763689
H -2.742145 1.176668 2.350519
H -1.607315 -1.081808 2.775447
H -0.621319 -2.523111 4.367450
H -1.032248 -1.373245 5.645658
H 1.230556 -2.557777 5.880609
H -3.644289 -0.699867 1.719392

237
active_int4
C 3.192669 2.596278 4.237633
C 3.433554 3.964530 3.639858
O 2.859388 4.970520 4.069818
N 4.321579 4.038222 2.616350
C 4.659497 5.305843 2.009727
C 3.575557 5.947789 1.143944
O 3.673384 7.117573 0.782724
N 2.552127 5.132647 0.822684
C 1.427334 5.541985 0.019324
C 0.292355 6.186466 0.828405
O -0.687709 6.656932 0.246118
C 0.890169 4.355439 -0.806497
C -0.077916 3.399657 -0.105183
C 0.471334 2.516246 1.000198
N -0.406701 1.650012 1.519270
O 1.651782 2.567536 1.387143
N 0.410539 6.164624 2.168522
C -0.595487 6.773441 3.014307
C -5.241421 -2.438466 2.501185
C -4.510479 -1.385653 3.301322
O -4.324763 -1.497579 4.511490
N -4.034897 -0.343987 2.578057
C -3.347296 0.795849 3.163197
C -2.269498 0.335008 4.163344
O -2.128726 0.831992 5.269340
C -4.270933 1.876182 3.764426
C -3.522994 3.213785 3.806726

C -5.562502 2.009444 2.954806
N -1.428984 -0.641724 3.698056
C -0.623049 -1.374608 4.635251
C 0.859539 -0.985054 4.647423
O 1.299438 -0.092489 3.920437
N 1.629024 -1.700990 5.490078
C 3.068016 -1.500489 5.695612
C -3.809590 -3.832991 -1.688709
C -2.877522 -4.699833 -0.830058
O -2.475285 -5.786129 -1.245532
C -3.528052 -2.323322 -1.640464
N -2.545776 -4.207939 0.392520
C -1.434943 -4.729502 1.183749
C -1.721321 -4.660513 2.678627
C -0.658688 -5.374037 3.529437
C 0.752779 -4.807797 3.332766
C -1.062238 -5.361710 5.007106
C 0.684319 -8.610512 -0.999262
C 1.298454 -7.308110 -0.491050
C 0.978679 -6.130959 -1.409696
C 1.677227 -4.851654 -0.980791
N 1.840584 -3.913683 -1.937967
O 2.058448 -4.673077 0.178080
C 8.882127 -3.297330 1.868725
C 7.427732 -2.842922 1.968205
C 7.297587 -1.428987 2.549397
C 5.854948 -0.935679 2.478264
O 4.989375 -1.533138 3.149657
O 5.601699 0.049605 1.700642
C -2.064930 3.680696 -4.362898
C -1.201985 2.656996 -3.638717
O -1.685336 1.783016 -2.897423
C -3.195661 4.314934 -3.527413
C -4.360653 3.358872 -3.250371
C -2.665353 4.935960 -2.233833
N 0.118063 2.724611 -3.856554
C 1.042702 1.719366 -3.379228
C 2.344380 1.835083 -4.183969
O 2.351335 2.421660 -5.266855
N 3.384117 1.219044 -3.606190
C 4.732523 1.214281 -4.139223
C 5.129842 -0.141522 -4.753047
O 5.867414 -0.192720 -5.736363
C 5.713839 1.580532 -2.997810
C 7.170974 1.558152 -3.441791
O 5.357907 2.862429 -2.515245
N 4.675144 -1.233583 -4.096342
C 5.167975 -2.571572 -4.358191
C 5.759534 -3.206099 -3.087853
C 6.886505 -2.388621 -2.503813
C 8.161134 -2.413688 -3.069983
C 6.674090 -1.541743 -1.412489
C 9.193807 -1.629901 -2.563296
C 7.689248 -0.742810 -0.900706
C 8.953963 -0.791228 -1.477022
O 9.932096 0.007701 -0.940692
C -8.900905 -0.547887 -3.795175
C -7.966980 0.550119 -3.245213
C -7.245570 0.244726 -1.960934
C -5.903755 0.166127 -1.665082
N -7.885842 0.078938 -0.758071
C -6.938987 -0.085073 0.183465
N -5.710329 -0.042992 -0.313511
C -13.977325 -0.139521 -2.012157
C -13.794819 -1.027492 -0.781942
C -12.680178 -0.527209 0.134546
C -11.274172 -0.582627 -0.496464
O -11.040968 -1.460876 -1.352071
O -10.448125 0.279269 -0.058086
N 2.885637 0.008696 1.661941
C 2.608443 -1.066807 0.681357
C 3.034912 -0.571890 -0.704999
O 3.132694 0.673172 -0.858800
C 1.130772 -1.524840 0.679632
C 0.263824 -0.907416 -0.427543
C -1.162694 -1.401518 -0.364284
N -2.926982 1.594071 -0.105791
O -1.804366 -1.453548 0.675080
O 3.188600 -1.435519 -1.601547
O -3.467893 -0.020613 -4.179748
O 5.047877 2.333767 0.298973
H -8.327330 -1.445782 -4.052096
H -0.404262 -8.518682 -1.088621
H 8.953610 -4.319746 1.480284
H 3.266992 2.674470 5.325367
H 5.538433 5.153644 1.377749
H -2.497127 3.163436 -5.230936
H 0.615888 0.720019 -3.528436
H 4.356386 -3.206403 -4.738243
H -13.057020 -0.126316 -2.604122
H -14.210909 0.892939 -1.720941
H -9.402279 -0.188161 -4.701066
H -4.846477 -3.994331 -1.368663
H -1.279711 -5.767002 0.874290
H 0.901678 -9.442854 -0.320435
H 9.374948 -3.272648 2.849229
H 3.249475 -0.557221 6.221539
H 3.438547 -2.330180 6.299772
H -6.166285 -2.701342 3.020528
H -0.299557 6.630534 4.054837
H -0.688735 7.843710 2.801798
H -14.790909 -0.501027 -2.653630
H -9.658394 -0.829262 -3.053973
H 1.078331 -8.873343 -1.988801
H 5.924460 -2.487305 -5.142649
H -1.571560 6.306607 2.848123
H 3.604197 -1.490255 4.742133
H 9.444301 -2.641346 1.193216
H -0.520068 -4.177467 0.936562
H -3.716903 -4.211776 -2.708695
H -1.420675 4.475315 -4.757097
H 1.235663 1.830290 -2.307021
H 4.910788 6.034416 2.784569
H 2.179116 2.270756 3.990388
H 3.899106 1.841732 3.877798
H -14.738520 -1.076389 -0.219037
H -13.551163 -2.044870 -1.105559
H -12.874128 0.500874 0.462445
H -12.641778 -1.142483 1.044318
H -7.204104 0.797845 -3.995315
H -8.556918 1.464297 -3.095514
H -8.931820 0.106326 -0.566290
H -5.065785 0.241384 -2.345619
H -7.186841 -0.229227 1.226054
H -5.474819 -2.119895 1.480998
H -4.619881 -3.339764 2.456068
H -2.783819 -3.242961 0.589343
H -1.811185 -3.608613 2.989313
H -2.700401 -5.119793 2.867319
H -0.639984 -6.424430 3.201481
H -0.336518 -5.913301 5.616410
H -1.116782 -4.336968 5.396312
H -2.048541 -5.815300 5.156672
H 1.464006 -5.315831 3.995015
H 1.118241 -4.926068 2.308018
H 0.795636 -3.738065 3.573684
H 2.385788 -7.414609 -0.398127
H 0.930442 -7.082342 0.515779
H 1.258938 -6.370084 -2.444596
H -0.105517 -5.946557 -1.417005
H 6.979529 -2.866309 0.968409
H 6.846899 -3.533479 2.589279
H 7.607635 -1.442702 3.602163
H 7.954663 -0.737082 2.013705
H 6.110879 -4.214224 -3.343948
H 4.964347 -3.323065 -2.343003
H 4.085644 -1.117561 -3.270691
H 1.489153 -4.061213 -2.870611
H 2.332304 -3.038424 -1.729768
H 5.538667 1.582480 0.688818
H 4.236512 1.902060 -0.037642
H -2.825731 0.532713 -3.695528
H -4.170847 0.584601 -4.440887
H -4.064087 2.543508 -2.586076
H -5.188265 3.893324 -2.771474
H -1.853334 5.648334 -2.413345
H -3.457697 5.471973 -1.701573
H -3.587036 5.128907 -4.153678
H 0.495263 3.327683 -4.580020
H 3.283071 0.975288 -2.613862
H -0.123143 1.052150 2.292404
H -1.333578 1.535985 1.084741
H 4.799518 1.960923 -4.933774
H 5.576167 0.838607 -2.198297
H 7.486447 0.557171 -3.749939
H 7.321300 2.246977 -4.279599
H 7.799327 1.878016 -2.604497
H 8.354953 -3.054218 -3.927619
H 10.181144 -1.666453 -3.020575
H 7.491357 -0.081396 -0.063848
H 5.689764 -1.494248 -0.954863
H 10.720353 -0.043066 -1.493424
H 5.395541 2.838548 -1.541333
H 4.607692 3.203845 2.108253
H 2.554951 4.164155 1.134209
H 1.258805 5.802436 2.592538
H 3.937051 0.122148 1.733424
H 2.461935 0.914396 1.399767
H 2.536090 -0.224190 2.601204
H 3.236140 -1.912548 0.972909
H 1.137803 -2.612404 0.564063
H 0.689414 -1.304359 1.657009
H 0.217911 0.182805 -0.330395
H 0.683654 -1.130573 -1.412300
H -2.516319 1.518247 -1.038485
H -3.325357 2.530968 -0.062097
H -2.283754 4.161443 -1.561526
H -4.746955 2.923835 -4.181432
H -6.184233 1.113265 3.034090
H -6.151657 2.857507 3.320472
H -5.359850 2.176025 1.888950
H -3.278360 3.549688 2.789399
H -4.144792 3.987077 4.271604
H -2.594062 3.135126 4.377074
H -4.519461 1.577258 4.788045
H -2.824811 1.255353 2.319057
H -1.633766 -1.055831 2.793295
H -0.697147 -2.443359 4.415931
H -1.035266 -1.209890 5.636868
H 1.164923 -2.402433 6.047429
H -4.300292 -0.280233 1.598853
H 0.347668 4.767554 -1.661216
H 1.752973 3.808751 -1.202923
H 1.775386 6.317980 -0.669424
H -0.510009 2.729997 -0.859439
H -0.926173 3.960632 0.305842
S -1.785992 -1.873173 -1.947709
H -3.732044 0.960619 -0.098296
H -4.086535 -1.817035 -2.430369
H -3.832671 -1.858160 -0.697480

237
active_int4
C 3.192669 2.596278 4.237633
C 3.433554 3.964530 3.639858
O 2.859388 4.970520 4.069818
N 4.321579 4.038222 2.616350
C 4.659497 5.305843 2.009727
C 3.575557 5.947789 1.143944
O 3.673384 7.117573 0.782724
N 2.552127 5.132647 0.822684
C 1.427334 5.541985 0.019324
C 0.292355 6.186466 0.828405
O -0.687709 6.656932 0.246118
C 0.890169 4.355439 -0.806497
C -0.077916 3.399657 -0.105183
C 0.471334 2.516246 1.000198
N -0.406701 1.650012 1.519270
O 1.651782 2.567536 1.387143
N 0.410539 6.164624 2.168522
C -0.595487 6.773441 3.014307
C -5.241421 -2.438466 2.501185
C -4.510479 -1.385653 3.301322
O -4.324763 -1.497579 4.511490
N -4.034897 -0.343987 2.578057
C -3.347296 0.795849 3.163197
C -2.269498 0.335008 4.163344
O -2.128726 0.831992 5.269340
C -4.270933 1.876182 3.764426
C -3.522994 3.213785 3.806726
C -5.562502 2.009444 2.954806
N -1.428984 -0.641724 3.698056
C -0.623049 -1.374608 4.635251
C 0.859539 -0.985054 4.647423
O 1.299438 -0.092489 3.920437
N 1.629024 -1.700990 5.490078
C 3.068016 -1.500489 5.695612
C -3.809590 -3.832991 -1.688709
C -2.877522 -4.699833 -0.830058
O -2.475285 -5.786129 -1.245532
C -3.528052 -2.323322 -1.640464
N -2.545776 -4.207939 0.392520

C -1.434943 -4.729502 1.183749
C -1.721321 -4.660513 2.678627
C -0.658688 -5.374037 3.529437
C 0.752779 -4.807797 3.332766
C -1.062238 -5.361710 5.007106
C 0.684319 -8.610512 -0.999262
C 1.298454 -7.308110 -0.491050
C 0.978679 -6.130959 -1.409696
C 1.677227 -4.851654 -0.980791
N 1.840584 -3.913683 -1.937967
O 2.058448 -4.673077 0.178080
C 8.882127 -3.297330 1.868725
C 7.427732 -2.842922 1.968205
C 7.297587 -1.428987 2.549397
C 5.854948 -0.935679 2.478264
O 4.989375 -1.533138 3.149657
O 5.601699 0.049605 1.700642
C -2.064930 3.680696 -4.362898
C -1.201985 2.656996 -3.638717
O -1.685336 1.783016 -2.897423
C -3.195661 4.314934 -3.527413
C -4.360653 3.358872 -3.250371
C -2.665353 4.935960 -2.233833
N 0.118063 2.724611 -3.856554
C 1.042702 1.719366 -3.379228
C 2.344380 1.835083 -4.183969
O 2.351335 2.421660 -5.266855
N 3.384117 1.219044 -3.606190
C 4.732523 1.214281 -4.139223
C 5.129842 -0.141522 -4.753047
O 5.867414 -0.192720 -5.736363
C 5.713839 1.580532 -2.997810
C 7.170974 1.558152 -3.441791
O 5.357907 2.862429 -2.515245
N 4.675144 -1.233583 -4.096342
C 5.167975 -2.571572 -4.358191
C 5.759534 -3.206099 -3.087853
C 6.886505 -2.388621 -2.503813
C 8.161134 -2.413688 -3.069983
C 6.674090 -1.541743 -1.412489
C 9.193807 -1.629901 -2.563296
C 7.689248 -0.742810 -0.900706
C 8.953963 -0.791228 -1.477022
O 9.932096 0.007701 -0.940692
C -8.900905 -0.547887 -3.795175
C -7.966980 0.550119 -3.245213
C -7.245570 0.244726 -1.960934
C -5.903755 0.166127 -1.665082
N -7.885842 0.078938 -0.758071
C -6.938987 -0.085073 0.183465
N -5.710329 -0.042992 -0.313511
C -13.977325 -0.139521 -2.012157
C -13.794819 -1.027492 -0.781942
C -12.680178 -0.527209 0.134546
C -11.274172 -0.582627 -0.496464
O -11.040968 -1.460876 -1.352071
O -10.448125 0.279269 -0.058086
N 2.885637 0.008696 1.661941
C 2.608443 -1.066807 0.681357
C 3.034912 -0.571890 -0.704999
O 3.132694 0.673172 -0.858800
C 1.130772 -1.524840 0.679632
C 0.263824 -0.907416 -0.427543
C -1.162694 -1.401518 -0.364284
N -2.926982 1.594071 -0.105791
O -1.804366 -1.453548 0.675080
O 3.188600 -1.435519 -1.601547
O -3.467893 -0.020613 -4.179748
O 5.047877 2.333767 0.298973
H -8.327330 -1.445782 -4.052096
H -0.404262 -8.518682 -1.088621
H 8.953610 -4.319746 1.480284
H 3.266992 2.674470 5.325367
H 5.538433 5.153644 1.377749
H -2.497127 3.163436 -5.230936
H 0.615888 0.720019 -3.528436
H 4.356386 -3.206403 -4.738243
H -13.057020 -0.126316 -2.604122
H -14.210909 0.892939 -1.720941
H -9.402279 -0.188161 -4.701066
H -4.846477 -3.994331 -1.368663
H -1.279711 -5.767002 0.874290
H 0.901678 -9.442854 -0.320435
H 9.374948 -3.272648 2.849229
H 3.249475 -0.557221 6.221539
H 3.438547 -2.330180 6.299772
H -6.166285 -2.701342 3.020528
H -0.299557 6.630534 4.054837
H -0.688735 7.843710 2.801798
H -14.790909 -0.501027 -2.653630
H -9.658394 -0.829262 -3.053973
H 1.078331 -8.873343 -1.988801
H 5.924460 -2.487305 -5.142649
H -1.571560 6.306607 2.848123
H 3.604197 -1.490255 4.742133
H 9.444301 -2.641346 1.193216
H -0.520068 -4.177467 0.936562
H -3.716903 -4.211776 -2.708695
H -1.420675 4.475315 -4.757097
H 1.235663 1.830290 -2.307021
H 4.910788 6.034416 2.784569
H 2.179116 2.270756 3.990388
H 3.899106 1.841732 3.877798
H -14.738520 -1.076389 -0.219037
H -13.551163 -2.044870 -1.105559
H -12.874128 0.500874 0.462445
H -12.641778 -1.142483 1.044318
H -7.204104 0.797845 -3.995315
H -8.556918 1.464297 -3.095514
H -8.931820 0.106326 -0.566290
H -5.065785 0.241384 -2.345619
H -7.186841 -0.229227 1.226054
H -5.474819 -2.119895 1.480998
H -4.619881 -3.339764 2.456068
H -2.783819 -3.242961 0.589343
H -1.811185 -3.608613 2.989313
H -2.700401 -5.119793 2.867319
H -0.639984 -6.424430 3.201481
H -0.336518 -5.913301 5.616410
H -1.116782 -4.336968 5.396312
H -2.048541 -5.815300 5.156672
H 1.464006 -5.315831 3.995015
H 1.118241 -4.926068 2.308018
H 0.795636 -3.738065 3.573684
H 2.385788 -7.414609 -0.398127
H 0.930442 -7.082342 0.515779
H 1.258938 -6.370084 -2.444596
H -0.105517 -5.946557 -1.417005
H 6.979529 -2.866309 0.968409
H 6.846899 -3.533479 2.589279
H 7.607635 -1.442702 3.602163
H 7.954663 -0.737082 2.013705
H 6.110879 -4.214224 -3.343948
H 4.964347 -3.323065 -2.343003
H 4.085644 -1.117561 -3.270691
H 1.489153 -4.061213 -2.870611
H 2.332304 -3.038424 -1.729768
H 5.538667 1.582480 0.688818
H 4.236512 1.902060 -0.037642
H -2.825731 0.532713 -3.695528
H -4.170847 0.584601 -4.440887
H -4.064087 2.543508 -2.586076
H -5.188265 3.893324 -2.771474
H -1.853334 5.648334 -2.413345
H -3.457697 5.471973 -1.701573
H -3.587036 5.128907 -4.153678
H 0.495263 3.327683 -4.580020
H 3.283071 0.975288 -2.613862
H -0.123143 1.052150 2.292404
H -1.333578 1.535985 1.084741
H 4.799518 1.960923 -4.933774
H 5.576167 0.838607 -2.198297
H 7.486447 0.557171 -3.749939
H 7.321300 2.246977 -4.279599
H 7.799327 1.878016 -2.604497
H 8.354953 -3.054218 -3.927619
H 10.181144 -1.666453 -3.020575
H 7.491357 -0.081396 -0.063848
H 5.689764 -1.494248 -0.954863
H 10.720353 -0.043066 -1.493424
H 5.395541 2.838548 -1.541333
H 4.607692 3.203845 2.108253
H 2.554951 4.164155 1.134209
H 1.258805 5.802436 2.592538
H 3.937051 0.122148 1.733424
H 2.461935 0.914396 1.399767
H 2.536090 -0.224190 2.601204
H 3.236140 -1.912548 0.972909
H 1.137803 -2.612404 0.564063
H 0.689414 -1.304359 1.657009
H 0.217911 0.182805 -0.330395
H 0.683654 -1.130573 -1.412300
H -2.516319 1.518247 -1.038485
H -3.325357 2.530968 -0.062097
H -2.283754 4.161443 -1.561526
H -4.746955 2.923835 -4.181432
H -6.184233 1.113265 3.034090
H -6.151657 2.857507 3.320472
H -5.359850 2.176025 1.888950
H -3.278360 3.549688 2.789399
H -4.144792 3.987077 4.271604
H -2.594062 3.135126 4.377074
H -4.519461 1.577258 4.788045
H -2.824811 1.255353 2.319057
H -1.633766 -1.055831 2.793295
H -0.697147 -2.443359 4.415931
H -1.035266 -1.209890 5.636868
H 1.164923 -2.402433 6.047429
H -4.300292 -0.280233 1.598853
H 0.347668 4.767554 -1.661216
H 1.752973 3.808751 -1.202923
H 1.775386 6.317980 -0.669424
H -0.510009 2.729997 -0.859439
H -0.926173 3.960632 0.305842
S -1.785992 -1.873173 -1.947709
H -3.732044 0.960619 -0.098296
H -4.086535 -1.817035 -2.430369
H -3.832671 -1.858160 -0.697480

237
active_TS2
C 3.192891 2.596576 4.237136
C 3.356058 3.938882 3.560087
O 2.732861 4.936555 3.943505
N 4.261602 4.003756 2.555024
C 4.587295 5.266326 1.929230
C 3.469530 5.904988 1.104830
O 3.493705 7.096908 0.816935
N 2.486915 5.056532 0.738130
C 1.315717 5.468646 0.001810
C 0.311654 6.272828 0.831605
O -0.613248 6.864165 0.274601
C 0.580867 4.228450 -0.536980
C -0.259342 3.457881 0.486129
C 0.523007 2.671825 1.521845
N -0.161210 2.220291 2.588053
O 1.706675 2.357083 1.340296
N 0.408087 6.164104 2.171702
C -0.595319 6.773573 3.013624
C -5.241320 -2.438182 2.501169
C -4.846388 -1.202424 3.287830
O -5.239161 -0.974594 4.425463
N -4.046673 -0.342700 2.589972
C -3.352302 0.777257 3.210951
C -2.378286 0.313216 4.324156
O -2.346587 0.823023 5.436057
C -4.254504 1.935437 3.677467
C -3.437267 3.230014 3.762183
C -5.449590 2.126368 2.744203
N -1.533029 -0.687583 3.951358
C -0.677902 -1.325821 4.909616
C 0.803893 -0.942612 4.816367
O 1.203855 -0.010351 4.111258
N 1.618847 -1.689551 5.581131
C 3.068260 -1.500085 5.695484
C -3.809651 -3.832942 -1.688746
C -3.005177 -4.696055 -0.709177
O -2.917350 -5.911806 -0.886560
C -3.481529 -2.346810 -1.726032
N -2.460225 -4.076738 0.380516
C -1.434772 -4.729443 1.183833
C -1.584920 -4.515075 2.684395
C -0.453424 -5.208381 3.464133
C 0.906959 -4.533231 3.243998
C -0.787680 -5.317416 4.954976
C 0.684305 -8.610567 -0.999009
C 1.208474 -7.255448 -0.528330
C 0.748991 -6.117180 -1.436341
C 1.511400 -4.823002 -1.188906
N 1.422057 -3.900520 -2.171267
O 2.197467 -4.651946 -0.177185
C 8.882203 -3.297328 1.868388

C 7.384931 -3.023907 1.980947
C 7.094678 -1.668849 2.639206
C 5.616897 -1.307426 2.528601
O 4.778933 -2.028209 3.104830
O 5.311822 -0.288214 1.814268
C -2.065067 3.680357 -4.363510
C -1.130208 2.833844 -3.511276
O -1.514706 2.299085 -2.454449
C -3.173449 4.442882 -3.614000
C -4.297312 3.530398 -3.112331
C -2.624067 5.320109 -2.486102
N 0.104882 2.638148 -3.980731
C 1.037873 1.696222 -3.399292
C 2.352443 1.774753 -4.188497
O 2.370608 2.288092 -5.309429
N 3.392737 1.223419 -3.553031
C 4.755822 1.214666 -4.044483
C 5.177026 -0.127652 -4.677036
O 5.998977 -0.150825 -5.593410
C 5.696203 1.528496 -2.852421
C 7.169929 1.535333 -3.237342
O 5.319594 2.782222 -2.308746
N 4.660720 -1.235320 -4.100224
C 5.168025 -2.571645 -4.358346
C 5.643863 -3.237311 -3.056290
C 6.746224 -2.453360 -2.387113
C 8.054418 -2.502037 -2.870286
C 6.482158 -1.607281 -1.307207
C 9.068521 -1.740942 -2.296899
C 7.479287 -0.829387 -0.730717
C 8.775922 -0.899525 -1.226159
O 9.734542 -0.121735 -0.624219
C -8.900796 -0.547948 -3.795144
C -8.130486 0.747203 -3.514040
C -7.135065 0.586085 -2.412057
C -5.777016 0.449463 -2.451237
N -7.518027 0.505480 -1.088665
C -6.432054 0.336732 -0.345708
N -5.360847 0.304168 -1.147235
C -13.977371 -0.139509 -2.012151
C -13.517637 -0.123024 -0.551018
C -12.300349 0.766353 -0.279642
C -10.930331 0.182562 -0.671447
O -10.847281 -0.970723 -1.118351
O -9.947432 0.985910 -0.470311
N 2.634146 -0.362374 1.579081
C 2.273021 -1.287683 0.472596
C 2.777861 -0.675847 -0.842504
O 3.081070 0.547924 -0.827714
C 0.761924 -1.542740 0.410999
C -0.042584 -0.348703 -0.091386
C -1.531964 -0.501543 0.103421
N -2.386332 0.496134 -0.396760
O -1.973415 -1.214881 1.028220
O 2.800924 -1.420705 -1.847209
O -3.078640 0.329589 -3.953709
O 4.948326 2.079750 0.497952
H -8.210732 -1.348195 -4.084898
H -0.411264 -8.615098 -1.041190
H 9.074830 -4.277856 1.417889
H 3.571232 1.765911 3.635625
H 5.443693 5.102484 1.269200
H -2.520250 2.987015 -5.084491
H 0.642143 0.673575 -3.464672
H 4.384754 -3.187517 -4.817676
H -13.172934 -0.508840 -2.656273
H -14.251774 0.868422 -2.349830
H -9.610268 -0.394592 -4.616220
H -4.870120 -3.981141 -1.434742
H -1.488206 -5.798258 0.958773
H 1.001402 -9.415748 -0.326067
H 9.366622 -3.276772 2.853527
H 3.413183 -2.069845 6.560409
H 3.594400 -1.828074 4.792672
H -5.957086 -2.171115 1.715099
H -1.589726 6.389158 2.761902
H -0.365451 6.532281 4.053261
H -14.849590 -0.790207 -2.146422
H -9.464451 -0.868812 -2.912570
H 1.056327 -8.846839 -2.003644
H 5.988508 -2.472267 -5.073345
H -0.615026 7.861427 2.887594
H 3.275242 -0.438838 5.857422
H 9.364787 -2.538540 1.241160
H -0.451244 -4.374236 0.850874
H -3.650969 -4.281745 -2.672718
H -1.462656 4.389385 -4.945109
H 1.202655 1.912040 -2.340022
H 4.869314 6.001647 2.687095
H 3.744831 2.623459 5.183339
H 2.140213 2.427438 4.463976
H -14.350790 0.220088 0.078671
H -13.277904 -1.145267 -0.239464
H -12.405475 1.737690 -0.780776
H -12.236199 0.996730 0.791502
H -7.593613 1.078499 -4.409794
H -8.834830 1.541229 -3.241120
H -8.590037 0.616624 -0.765595
H -5.053070 0.427510 -3.258836
H -6.401180 0.250933 0.728767
H -4.378769 -2.908826 2.014164
H -5.716402 -3.151907 3.175083
H -2.358431 -3.068880 0.328874
H -1.591338 -3.437895 2.908217
H -2.559005 -4.908202 3.003799
H -0.377631 -6.235701 3.077479
H 0.007882 -5.841015 5.498596
H -0.909775 -4.328919 5.416685
H -1.724896 -5.862729 5.114035
H 1.240575 -4.582155 2.202422
H 0.872573 -3.474007 3.531299
H 1.684288 -5.009812 3.852719
H 2.303305 -7.268447 -0.493046
H 0.877910 -7.055047 0.497537
H 0.882795 -6.395970 -2.489971
H -0.326688 -5.930966 -1.307895
H 6.944329 -3.042188 0.978347
H 6.886085 -3.811358 2.556190
H 7.364022 -1.718539 3.702313
H 7.702312 -0.885858 2.175221
H 5.987295 -4.251945 -3.296375
H 4.790809 -3.340464 -2.375689
H 3.963181 -1.140337 -3.359184
H 1.873596 -2.993796 -2.023299
H 0.629424 -3.920090 -2.799379
H 5.380173 1.302827 0.909687
H 4.141983 1.676871 0.110394
H -2.609704 -0.471184 -3.617139
H -2.691884 1.027140 -3.402449
H -3.942762 2.875853 -2.311422
H -5.128563 4.126996 -2.719095
H -1.787611 5.945242 -2.818692
H -3.401049 5.987123 -2.097887
H -3.612986 5.109133 -4.370209
H 0.405665 3.020943 -4.871709
H 3.261830 0.976710 -2.565092
H 0.279111 1.563685 3.234180
H -1.065740 2.597739 2.823227
H 4.861381 1.982216 -4.814952
H 5.530572 0.746474 -2.099683
H 7.504363 0.553490 -3.583185
H 7.350411 2.266822 -4.032011
H 7.760325 1.815013 -2.358751
H 8.288640 -3.143599 -3.716986
H 10.082265 -1.796834 -2.690119
H 7.244716 -0.170138 0.097579
H 5.474059 -1.543566 -0.906119
H 10.553704 -0.185108 -1.128506
H 5.330964 2.695676 -1.338954
H 4.552665 3.157336 2.060665
H 2.576026 4.071857 0.967633
H 1.240882 5.767167 2.598990
H 3.694362 -0.333868 1.703178
H 2.324074 0.602976 1.386473
H 2.234992 -0.636325 2.481855
H 2.784164 -2.237073 0.655110
H 0.577562 -2.395312 -0.246425
H 0.414677 -1.835819 1.409475
H 0.218153 0.543838 0.490897
H 0.180089 -0.116929 -1.134607
H -1.980169 1.072673 -1.133757
H -2.767727 1.065291 0.354490
H -2.273280 4.706532 -1.650760
H -4.685815 2.895426 -3.916330
H -6.135650 1.276886 2.799458
H -6.009424 3.024352 3.026341
H -5.130734 2.251170 1.700235
H -3.094244 3.539896 2.762453
H -4.050243 4.048818 4.152912
H -2.575841 3.120959 4.428439
H -4.621370 1.685967 4.676641
H -2.706402 1.166233 2.410268
H -1.556182 -1.028345 2.992157
H -0.769491 -2.411819 4.809721
H -1.036206 -1.046434 5.905780
H 1.214876 -2.499064 6.028281
H -3.539763 -0.752780 1.803997
H -0.092282 4.556423 -1.330460
H 1.319064 3.557782 -0.986992
H 1.616453 6.105813 -0.834755
H -0.837866 2.709831 -0.070969
H -0.991266 4.111945 0.974608
S -1.752981 -1.982714 -2.169172
H -4.379306 0.176285 -0.873683
H -4.164024 -1.871717 -2.438985
H -3.708912 -1.902466 -0.743038

237
active_TS3
C -3.809790 -3.832767 -1.688920
C -2.911835 -4.673129 -0.773400
O -2.654093 -5.837540 -1.094552
C -3.720870 -2.321847 -1.574004
N -2.474489 -4.107668 0.376813
C -1.434876 -4.729891 1.183110
C -1.717407 -4.661344 2.677652
C -0.668077 -5.420283 3.507413
C 0.763481 -4.911321 3.289382
C -1.039886 -5.408985 4.993537
C -8.900696 -0.548208 -3.794838
C -8.014174 0.670378 -3.485963
C -7.039246 0.499423 -2.357627
C -5.668777 0.476330 -2.346524
N -7.428560 0.414776 -1.040410
C -6.323269 0.355915 -0.284831
N -5.228189 0.392824 -1.040859
C -13.977334 -0.139426 -2.012282
C -13.558110 -0.244180 -0.543569
C -12.327066 0.591665 -0.184293
C -10.963517 0.030771 -0.645063
O -10.914044 -1.094866 -1.174862
O -9.974979 0.802871 -0.410740
N 2.456140 -0.411819 1.456666
C 2.037037 -1.281262 0.327592
C 2.580101 -0.660334 -0.967204
O 2.955964 0.540141 -0.917924
C 0.519471 -1.512031 0.267414
C -0.346428 -0.309770 -0.121990
C -1.841568 -0.686519 -0.222247
N -2.680723 0.558998 -0.477577
O -2.297987 -1.314366 0.839069
O 2.584736 -1.379112 -1.995108
H -8.285479 -1.408998 -4.081630
H -13.162231 -0.480210 -2.658333
H -14.216997 0.898265 -2.279889
H -9.571995 -0.314461 -4.628914
H -4.845338 -4.142054 -1.493632
H -1.360703 -5.774684 0.867644
H -14.861511 -0.754487 -2.220310
H -9.510223 -0.826507 -2.927631
H -0.475553 -4.253609 0.950305
H -3.577588 -4.162509 -2.706032
H -14.399664 0.070521 0.090985
H -13.347661 -1.292569 -0.306307
H -12.420388 1.612460 -0.578099
H -12.257345 0.703550 0.905644
H -7.434906 0.948586 -4.374569
H -8.661357 1.523595 -3.248019
H -8.452878 0.462058 -0.727226
H -4.972762 0.513252 -3.175228
H -6.325454 0.296041 0.793588
H -2.538285 -3.091403 0.494669
H -1.770691 -3.609299 2.993562
H -2.710128 -5.088570 2.871397
H -0.695431 -6.467596 3.170730
H -0.328943 -6.001091 5.582113
H -1.038887 -4.388156 5.397738
H -2.042903 -5.819049 5.157415
H 1.466956 -5.459776 3.927206
H 1.102140 -5.022013 2.254502
H 0.860703 -3.847616 3.543432
H 3.517359 -0.451154 1.571517

H 2.202395 0.575832 1.293245
H 2.048210 -0.680899 2.356244
H 2.508526 -2.257128 0.477594
H 0.354879 -2.323097 -0.447106
H 0.188543 -1.897560 1.239464
H -0.283541 0.452721 0.663250
H 0.001236 0.147129 -1.057158
H -2.298738 1.101854 -1.261749
H -2.611262 1.150411 0.353978
S -2.037065 -1.682708 -1.856992
H -3.921016 0.402332 -0.705250
H -4.409702 -1.861546 -2.285192
H -4.022343 -1.989118 -0.576557
C 3.192671 2.595095 4.238366
C 3.372709 3.929700 3.551623
O 2.777403 4.943263 3.935911
N 4.266770 3.969947 2.533488
C 4.607542 5.224849 1.900330
C 3.487328 5.884519 1.095620
O 3.525601 7.075890 0.808050
N 2.487678 5.050398 0.740724
C 1.323575 5.472443 -0.001078
C 0.302529 6.254949 0.830516
O -0.627063 6.836093 0.270451
C 0.611430 4.240251 -0.586056
C -0.246647 3.438986 0.399149
C 0.525818 2.664421 1.451860
N -0.149412 2.255234 2.538552
O 1.704837 2.333365 1.266843
N 0.399982 6.150186 2.170638
C -0.595767 6.772527 3.015148
C -5.241684 -2.439254 2.500970
C -4.808399 -1.232344 3.314362
O -5.099795 -1.079087 4.495953
N -4.077534 -0.333239 2.597185
C -3.332970 0.751661 3.216327
C -2.271510 0.225764 4.213510
O -2.128123 0.676723 5.342607
C -4.191712 1.881898 3.813086
C -3.363166 3.167640 3.918380
C -5.445393 2.129070 2.973485
N -1.480600 -0.769042 3.722116
C -0.610769 -1.493306 4.604910
C 0.839461 -0.993878 4.678532
O 1.232256 0.001060 4.060979
N 1.637628 -1.738265 5.466922
C 3.068002 -1.501940 5.695853
C 0.684693 -8.610691 -1.000625
C 1.241150 -7.255624 -0.576609
C 0.772964 -6.145497 -1.512379
C 1.442507 -4.815246 -1.221035
N 1.539745 -3.968705 -2.267245
O 1.874258 -4.533587 -0.100279
C 8.882497 -3.297963 1.868734
C 7.368924 -3.073503 1.901475
C 6.987654 -1.735793 2.552700
C 5.493185 -1.438236 2.411307
O 4.679598 -2.157516 3.021264
O 5.156170 -0.465917 1.645935
C -2.065443 3.680814 -4.361860
C -1.125476 2.792791 -3.564687
O -1.529382 2.104249 -2.606375
C -3.156614 4.420186 -3.562322
C -4.291572 3.503482 -3.099382
C -2.582507 5.221872 -2.392429
N 0.144758 2.718412 -3.969831
C 1.080244 1.757533 -3.425918
C 2.395422 1.854201 -4.210711
O 2.428350 2.429109 -5.300579
N 3.415606 1.239660 -3.600464
C 4.785835 1.222676 -4.066656
C 5.207889 -0.127232 -4.682559
O 6.057098 -0.165480 -5.572380
C 5.706749 1.530933 -2.855962
C 7.185397 1.558475 -3.219930
O 5.311147 2.770726 -2.296084
N 4.660994 -1.230042 -4.119580
C 5.167889 -2.571124 -4.358501
C 5.621460 -3.229542 -3.045144
C 6.728411 -2.453168 -2.375551
C 8.041198 -2.537511 -2.841358
C 6.466009 -1.578095 -1.318816
C 9.062205 -1.781353 -2.274443
C 7.470963 -0.804212 -0.750917
C 8.771798 -0.908371 -1.228486
O 9.735671 -0.132232 -0.634365
O -3.079165 0.392775 -4.344663
O 4.862342 1.991186 0.479250
H -0.411243 -8.592721 -1.019703
H 9.129256 -4.265867 1.417522
H 3.806538 2.592666 5.146111
H 5.448036 5.043611 1.224434
H -2.538661 3.024738 -5.105630
H 0.682218 0.738960 -3.521864
H 4.389897 -3.187215 -4.827277
H 0.999527 -9.405092 -0.314103
H 9.310367 -3.275992 2.879426
H 3.668133 -1.847796 4.847002
H 3.227137 -0.429325 5.832108
H -5.921792 -3.049949 3.095618
H -0.365900 6.529849 4.054335
H -0.603091 7.860279 2.887058
H 1.029906 -8.881356 -2.006258
H 5.999772 -2.479463 -5.060925
H -1.594003 6.397909 2.765891
H 3.353798 -2.027542 6.609313
H 9.373542 -2.515299 1.278428
H -1.467327 4.411139 -4.920774
H 1.249072 1.932240 -2.359525
H 4.918516 5.954980 2.651813
H 2.150218 2.472457 4.532979
H 3.491256 1.749830 3.613004
H -5.741848 -2.129699 1.576900
H -4.369264 -3.038372 2.214243
H 2.337146 -7.285319 -0.560023
H 0.928556 -7.016743 0.445699
H 0.965024 -6.427774 -2.556491
H -0.315038 -6.013515 -1.421006
H 6.985440 -3.098499 0.875598
H 6.869868 -3.884189 2.443958
H 7.230691 -1.776275 3.622426
H 7.570184 -0.923825 2.106874
H 5.952415 -4.251620 -3.270957
H 4.759970 -3.314287 -2.372264
H 3.926261 -1.126375 -3.417264
H 1.054340 -4.173091 -3.126344
H 1.902738 -3.017922 -2.128366
H 5.251156 1.176054 0.862625
H 4.031872 1.648399 0.088387
H -2.771076 -0.470390 -4.029627
H -2.667346 0.994715 -3.701846
H -3.950610 2.809412 -2.327160
H -5.112894 4.092927 -2.676785
H -1.742162 5.857226 -2.693982
H -3.345592 5.872303 -1.952961
H -3.587662 5.134497 -4.278063
H 0.472276 3.196748 -4.803290
H 3.262334 0.964140 -2.623011
H 0.286149 1.598949 3.189045
H -1.059369 2.626238 2.764178
H 4.910359 1.986642 -4.837696
H 5.537424 0.735799 -2.118128
H 7.533885 0.586965 -3.580710
H 7.369304 2.307916 -3.996960
H 7.761407 1.826139 -2.328279
H 8.273677 -3.203685 -3.669364
H 10.079371 -1.865602 -2.653313
H 7.238885 -0.120440 0.057383
H 5.457680 -1.486577 -0.922551
H 10.562024 -0.226695 -1.121830
H 5.284357 2.656715 -1.329188
H 4.532226 3.114493 2.040531
H 2.571716 4.063667 0.962049
H 1.239417 5.764505 2.594886
H -2.232809 4.552625 -1.599503
H -4.693007 2.908589 -3.926898
H -6.128013 1.276529 3.014708
H -5.983077 3.006838 3.348343
H -5.194528 2.316496 1.920891
H -3.087497 3.534193 2.916487
H -3.944302 3.964168 4.394801
H -2.459459 3.018297 4.517329
H -4.490487 1.574072 4.818889
H -2.756080 1.185027 2.385752
H -1.617328 -1.082768 2.757529
H -0.592756 -2.541144 4.296448
H -1.033018 -1.448989 5.614944
H 1.242968 -2.589389 5.839946
H -3.624902 -0.710785 1.753077
H -0.042414 4.580975 -1.389997
H 1.367982 3.586036 -1.029510
H 1.634720 6.132886 -0.815655
H -0.780696 2.680589 -0.189347
H -1.011630 4.071236 0.865205

237
active_TS4
H 5.372996 1.332810 0.821612
H 4.121435 1.745479 0.061714
H -2.896546 -0.544823 -4.141441
H -2.769853 0.893504 -3.667999
N 4.307158 4.018373 2.567269
N 2.534538 5.106070 0.772329
N -0.110770 2.249816 2.417456
N 0.403326 6.147267 2.175433
C 3.239612 2.586463 4.231828
C 3.404924 3.939116 3.576579
C 4.633764 5.292979 1.967205
C 3.517145 5.948478 1.154411
C 1.381308 5.521603 0.009641
C 0.319460 6.256938 0.834787
C 0.725195 4.294193 -0.645507
C -0.146137 3.433333 0.276170
C 0.598149 2.674418 1.358933
C -0.596131 6.772811 3.014844
O 2.789464 4.933593 3.977413
O 3.544068 7.144227 0.883989
O -0.615415 6.820194 0.265383
O 1.789976 2.367558 1.219094
H 3.791399 2.597505 5.178292
H 5.490167 5.143230 1.303780
H -0.372193 6.529972 4.055220
H -0.599060 7.860433 2.885301
H -1.593429 6.399964 2.760515
H 4.915836 6.012146 2.740293
H 2.187002 2.413164 4.456199
H 3.616354 1.764238 3.617859
H 0.312904 1.609371 3.091400
H -1.031183 2.611739 2.613519
H 4.591987 3.180612 2.057125
H 2.631930 4.117404 0.978849
H 1.245709 5.773870 2.604279
H -2.324069 1.200938 -1.281092
H -2.428572 1.461332 0.339691
H 0.090065 4.645499 -1.460593
H 1.516708 3.679461 -1.085099
H 1.701741 6.218970 -0.769993
H -0.608191 2.659840 -0.353352
H -0.965373 4.022469 0.705921
H -3.593915 0.549208 -0.403001
N -2.564955 -4.215302 0.414706
N 2.647292 -0.317118 1.512640
C 2.288372 -1.253852 0.421676
C -3.809103 -3.832998 -1.688923
C -2.886959 -4.700392 -0.813405
C -3.633421 -2.322180 -1.534136
C -1.434235 -4.730132 1.183976
C -1.649858 -4.625333 2.686919
C -0.516844 -5.291298 3.485698
C 0.855788 -4.658821 3.220997
C -0.844944 -5.306586 4.982001
C 2.746163 -0.640488 -0.910772
C 0.796324 -1.602747 0.399218
C -0.158752 -0.460771 0.015887
C -1.572313 -1.015959 -0.083891
O -2.495051 -5.792003 -1.229015
O -2.124263 -1.485055 0.921715
O 2.738180 -1.388000 -1.918060
O 3.064718 0.575876 -0.909032
S -1.910072 -1.745834 -1.741477
H -4.851057 -4.087543 -1.457192
H -1.297363 -5.777405 0.897990
H -0.525944 -4.193779 0.883989
H -3.621453 -4.149035 -2.718111
H -2.743951 -3.229778 0.590473
H -1.748333 -3.568069 2.974072
H -2.604691 -5.103578 2.941593
H -0.464084 -6.338952 3.153050
H -0.060747 -5.820669 5.550546
H -0.937809 -4.289079 5.383172
H -1.796268 -5.814297 5.177094
H 1.623809 -5.124253 3.850124

H 1.179581 -4.765237 2.180665
H 0.854809 -3.586303 3.454959
H 0.681717 -2.430593 -0.304351
H 0.513721 -1.997885 1.383106
H -0.172342 0.297058 0.803823
H 0.136040 0.017293 -0.924816
H -4.248808 -1.802588 -2.271418
H -3.976292 -1.964537 -0.558921
H 2.840142 -2.182476 0.596076
H 3.708633 -0.284901 1.621136
H 2.327517 0.645457 1.317774
H 2.265323 -0.585409 2.425143
N 1.714310 -3.984003 -2.239592
C 0.684672 -8.610392 -0.998965
C 1.303894 -7.282894 -0.572996
C 0.901014 -6.155589 -1.517892
C 1.600429 -4.845779 -1.205979
O 2.035217 -4.585461 -0.081728
H -0.408613 -8.537078 -1.029756
H 0.952386 -9.417028 -0.306816
H 1.026183 -8.901514 -2.000109
H 2.396876 -7.366514 -0.543342
H 0.991751 -7.024379 0.444746
H 1.112895 -6.441074 -2.557357
H -0.184383 -5.988503 -1.456897
H 1.230384 -4.168447 -3.104123
H 2.085047 -3.039921 -2.081052
C 8.882443 -3.297469 1.870482
C 7.385072 -3.008027 1.950600
C 7.092205 -1.644485 2.591282
C 5.618479 -1.272540 2.452524
O 4.766717 -1.965939 3.041665
O 5.331329 -0.274109 1.701324
H 9.073777 -4.284405 1.433671
H 9.347287 -3.271696 2.864693
H 9.384540 -2.549569 1.245391
H 6.965157 -3.030993 0.939081
H 6.867924 -3.786102 2.522614
H 7.342641 -1.686467 3.659139
H 7.713748 -0.870702 2.130344
N 0.184889 2.778688 -3.971700
N 3.448183 1.259356 -3.623551
N 4.679688 -1.223675 -4.120955
C -2.064953 3.680852 -4.361653
C -1.108663 2.755227 -3.629518
C -3.168028 4.329223 -3.498805
C -4.266737 3.353597 -3.062821
C -2.592667 5.079977 -2.296127
C 1.121281 1.806314 -3.448311
C 2.439055 1.903321 -4.225567
C 4.817511 1.228056 -4.094330
C 5.224026 -0.129916 -4.703093
C 5.748567 1.545761 -2.894691
C 7.225329 1.542978 -3.267867
C 5.168283 -2.571183 -4.357760
C 5.640807 -3.224656 -3.048585
C 6.756854 -2.447470 -2.394897
C 8.062415 -2.525519 -2.881430
C 6.509985 -1.580639 -1.327633
C 9.090795 -1.772847 -2.322954
C 7.521928 -0.811248 -0.766036
C 8.815745 -0.910699 -1.263932
O -1.508245 1.955433 -2.763045
O 2.485044 2.496542 -5.304272
O 6.056211 -0.181422 -5.608192
O 5.372963 2.803573 -2.362695
O 9.787997 -0.140367 -0.676341
H -2.531767 3.072075 -5.148108
H 0.715534 0.793114 -3.561250
H 4.375712 -3.183963 -4.806264
H 5.987092 -2.493750 -5.077286
H -1.486509 4.465719 -4.864372
H 1.289325 1.958744 -2.377413
H 5.968238 -4.247533 -3.275850
H 4.789177 -3.307261 -2.363306
H 3.971384 -1.109380 -3.393962
H -3.912145 2.671820 -2.285758
H -5.119467 3.903626 -2.649801
H -1.800005 5.781582 -2.579093
H -3.371924 5.653455 -1.784004
H -3.636191 5.069129 -4.163283
H 0.526594 3.324851 -4.755331
H 3.297315 0.984016 -2.646094
H 4.942071 1.984037 -4.873114
H 5.572239 0.769114 -2.138549
H 7.557031 0.558257 -3.608444
H 7.415519 2.271439 -4.063066
H 7.811070 1.822263 -2.386191
H 8.283444 -3.184035 -3.718567
H 10.102046 -1.851915 -2.718333
H 7.300435 -0.137022 0.053563
H 5.505826 -1.492941 -0.920430
H 10.606860 -0.228332 -1.177498
H 5.357171 2.718565 -1.392268
H -2.177760 4.376059 -1.566307
H -4.628357 2.743639 -3.897599
N -7.794026 0.226993 -0.843981
N -5.599321 0.154570 -0.502875
C -8.901015 -0.548219 -3.794676
C -7.998034 0.611866 -3.335956
C -7.212731 0.371015 -2.079069
C -5.857508 0.321042 -1.851190
C -6.801086 0.105545 0.055596
H -8.297760 -1.433097 -4.028524
H -9.449655 -0.258977 -4.698706
H -9.622040 -0.821669 -3.015128
H -7.278822 0.861771 -4.125479
H -8.622016 1.503235 -3.187510
H -8.831159 0.253496 -0.614091
H -5.062690 0.403594 -2.584055
H -6.994978 -0.009471 1.113199
C -13.977703 -0.139183 -2.012441
C -13.752385 -0.837145 -0.671411
C -12.617513 -0.208208 0.134533
C -11.214448 -0.383021 -0.484280
O -11.021483 -1.333375 -1.269341
O -10.350072 0.468572 -0.103070
H -13.072489 -0.204685 -2.623747
H -14.217410 0.922566 -1.867897
H -14.802856 -0.597701 -2.571900
H -14.680224 -0.806753 -0.081197
H -13.511282 -1.890173 -0.851054
H -12.794694 0.862833 0.290758
H -12.575223 -0.660706 1.135112
N -2.587120 0.772692 -0.392481
O -3.299088 0.324773 -4.256124
N -4.124958 -0.293855 2.579151
N -1.448838 -0.636576 3.697821
N 1.626968 -1.687794 5.472927
C -5.241616 -2.437589 2.500830
C -4.550268 -1.356236 3.305254
C -3.339068 0.810495 3.098118
C -2.280234 0.352192 4.128620
C -4.176306 2.000953 3.610912
C -3.330832 3.279179 3.610003
C -5.432237 2.196393 2.758825
C -0.628501 -1.334994 4.648855
C 0.852136 -0.927328 4.675349
C 3.068303 -1.500148 5.696856
O -4.356731 -1.455109 4.514655
O -2.146058 0.895880 5.216477
O 1.288146 0.027147 4.022811
H 3.356924 -2.125261 6.543735
H 3.649015 -1.771636 4.808914
H -6.059966 -2.854300 3.091290
H 3.257651 -0.451173 5.940595
H -5.625437 -2.077281 1.541514
H -4.521341 -3.239739 2.302708
H -6.132959 1.367311 2.883009
H -5.948164 3.117485 3.050499
H -5.193885 2.268166 1.689579
H -3.044830 3.551790 2.581452
H -3.904039 4.122868 4.008158
H -2.433999 3.172362 4.228049
H -4.468713 1.778451 4.642385
H -2.759535 1.161198 2.234904
H -1.632578 -1.052733 2.783429
H -0.690768 -2.407925 4.448794
H -1.043189 -1.158986 5.647589
H 1.192644 -2.497981 5.889782
H -4.301015 -0.303964 1.580677
O 4.946348 2.123397 0.430964

237
inactive_ES
C 0.131158 7.050402 7.018421
C -0.093657 8.373437 6.320230
O -0.890657 9.219956 6.720613
N 0.659128 8.568298 5.201765
C 0.527004 9.767959 4.407145
C -0.752479 9.895271 3.576434
O -1.027299 10.965836 3.043097
N -1.507288 8.779641 3.494412
C -2.727424 8.714446 2.723053
C -3.996645 8.880597 3.583052
O -5.109041 8.711456 3.087375
C -2.789290 7.477513 1.814420
C -2.701773 6.087082 2.483723
C -1.289205 5.648512 2.844205
N -0.712848 4.754546 2.031984
O -0.697517 6.107695 3.839081
N -3.816607 9.289265 4.856181
C -4.942481 9.678298 5.683645
C -4.455207 -5.190604 4.396074
C -3.905102 -3.846273 3.958835
O -3.882627 -3.516326 2.774846
N -3.490778 -3.058264 4.973958
C -3.019880 -1.692165 4.862075
C -1.803963 -1.571036 5.781102
O -1.758173 -2.178040 6.855334
C -4.151987 -0.709919 5.282915
C -3.662020 0.695513 5.642373
C -5.206596 -0.647624 4.174789
N -0.834467 -0.741241 5.348767
C 0.281950 -0.394520 6.185822
C 1.581824 -1.070867 5.749711
O 1.870284 -1.176249 4.560305
N 2.405841 -1.461940 6.742764
C 3.719999 -2.037816 6.506452
C -3.361924 -4.112156 -1.710999
C -2.125700 -4.716062 -1.050280
O -1.283635 -5.290849 -1.750834
C -4.127977 -3.049331 -0.924652
N -1.990339 -4.612228 0.286245
C -0.759530 -5.014700 0.955832
C -0.745757 -4.504324 2.392016
C 0.525728 -4.854020 3.176208
C 1.774703 -4.165784 2.617796
C 0.332767 -4.475553 4.648116
C 2.523243 -7.676857 -1.853818
C 2.528314 -6.312790 -1.162559
C 1.953522 -5.229825 -2.071630
C 2.028559 -3.831376 -1.478224
N 0.955158 -3.044980 -1.710420
O 3.016009 -3.436788 -0.854122
C 9.693856 -1.796739 2.830382
C 8.243913 -1.547725 2.404761
C 7.564183 -0.466144 3.244676
C 6.123019 -0.141135 2.831690
O 5.441940 -1.074942 2.314935
O 5.710416 1.029964 3.045558
C -2.898229 3.983890 -2.585212
C -1.662280 3.873313 -1.701180
O -1.734356 3.776608 -0.466910
C -4.126817 4.653474 -1.946415
C -4.771801 3.811224 -0.839795
C -3.804364 6.070374 -1.463588
N -0.478321 3.830931 -2.332699
C 0.738353 3.475651 -1.644544
C 1.961638 3.744164 -2.516183
O 1.925401 4.562586 -3.431451
N 3.041349 3.034721 -2.135897
C 4.403630 3.394018 -2.468921
C 5.153493 2.305708 -3.256808
O 5.943989 2.608245 -4.150172
C 5.195460 3.699735 -1.159386
C 6.539989 4.348539 -1.452957
O 4.433997 4.534823 -0.308417
N 4.942114 1.037123 -2.837357
C 5.749044 -0.085905 -3.269741
C 6.494996 -0.729816 -2.090797
C 7.390326 0.249859 -1.372374
C 8.594470 0.675169 -1.933066
C 7.010233 0.799442 -0.145849
C 9.399932 1.609543 -1.288927
C 7.801082 1.734060 0.513865
C 9.000117 2.138041 -0.063387
O 9.757787 3.063719 0.611777
C -8.711150 -1.474757 -3.779889
C -8.211545 -0.076082 -3.390309
C -7.408358 -0.062882 -2.127793
C -6.074160 0.156065 -1.884740

N -7.965280 -0.314935 -0.899810
C -6.976288 -0.237841 0.013084
N -5.807238 0.050971 -0.536307
C -13.879017 -2.174846 -2.729062
C -13.710137 -2.482276 -1.240474
C -12.808226 -1.481587 -0.518522
C -11.306020 -1.565958 -0.872223
O -10.880871 -2.587944 -1.447967
O -10.619039 -0.560842 -0.504902
N 2.905721 -0.333833 2.045798
C 2.007839 -0.783120 0.947452
C 2.293700 -0.013782 -0.354190
O 3.288797 0.763456 -0.374271
C 0.537911 -0.650249 1.359581
C 0.093057 0.811627 1.527901
C -1.370292 0.882841 1.909785
N -2.223797 1.227731 0.946990
O -1.736888 0.612863 3.066176
O 1.501380 -0.207675 -1.302072
O 3.847524 2.500876 1.643854
O -1.138037 0.723012 -1.772829
H -12.904562 -2.197765 -3.226638
H -7.863537 -2.153727 -3.931923
H 1.505290 -7.965022 -2.140872
H 10.159390 -2.577572 2.217932
H -0.404504 7.053033 7.968040
H -3.145989 2.952911 -2.872891
H -9.283486 -1.428278 -4.714381
H -14.316955 -1.178891 -2.877764
H -5.463419 10.540174 5.252662
H -4.567257 9.936885 6.675447
H 1.377763 9.833036 3.723476
H 5.111089 -0.836397 -3.752426
H 0.705747 2.424330 -1.336902
H -4.039042 -4.943094 -1.945940
H -0.666903 -6.108734 0.934180
H -4.047448 -5.968326 3.745229
H 3.663802 -3.125742 6.390996
H 4.375646 -1.791218 7.344584
H 9.752152 -2.113307 3.880088
H 2.924675 -8.458797 -1.198999
H -14.532617 -2.907145 -3.219545
H -9.357271 -1.895691 -3.000545
H -3.019537 -3.705175 -2.667198
H 0.083799 -4.611077 0.386648
H 3.134087 -7.656007 -2.764848
H 10.294494 -0.884859 2.725216
H -5.659090 8.856028 5.768213
H 4.132274 -1.614384 5.588346
H 0.845648 4.086143 -0.740582
H -2.627878 4.510680 -3.509545
H 6.451274 0.295900 -4.014920
H 0.556761 10.641416 5.062629
H -4.231986 -5.436489 5.438229
H -5.540977 -5.180610 4.258550
H 4.364570 4.283755 -3.100145
H -2.731921 9.597703 2.073606
H -0.248682 6.241395 6.383899
H 1.197070 6.873574 7.193083
H -14.698767 -2.493765 -0.757733
H -13.279160 -3.482903 -1.128506
H -13.143717 -0.452317 -0.697830
H -12.876948 -1.634040 0.567436
H -7.590275 0.342777 -4.191737
H -9.074262 0.591735 -3.273417
H -8.993217 -0.500239 -0.726781
H -7.144572 -0.398801 1.068495
H -5.289742 0.382593 -2.596230
H -2.660333 -4.080990 0.833310
H -0.887280 -3.414086 2.390916
H -1.611920 -4.923186 2.918903
H 0.671837 -5.943660 3.114257
H 1.220520 -4.723083 5.242404
H 0.161996 -3.396496 4.738917
H -0.530289 -4.987307 5.090246
H 2.668386 -4.462977 3.177943
H 1.959529 -4.391026 1.562739
H 1.677950 -3.079810 2.724554
H -3.474637 -3.427302 5.914795
H -2.738345 -1.503179 3.822586
H -5.590210 -1.639371 3.918317
H -6.052254 -0.023395 4.484488
H -4.774632 -0.215195 3.265354
H -4.607613 -1.148718 6.182488
H -3.145266 1.156760 4.796342
H -4.521132 1.322438 5.906370
H -2.984292 0.684999 6.503430
H -0.981544 -0.234300 4.468666
H 0.027510 -0.640955 7.220329
H 0.456999 0.686108 6.119373
H 2.041543 -1.464963 7.682668
H 3.543759 -6.034239 -0.862353
H 1.930046 -6.363913 -0.243401
H 2.532162 -5.194819 -3.006092
H 0.919382 -5.473374 -2.331878
H 0.141734 -3.425127 -2.172295
H 1.022437 -2.041652 -1.533464
H 7.080974 -1.572784 -2.479472
H 5.767098 -1.151560 -1.387713
H 8.909884 0.277601 -2.895119
H 6.072323 0.486614 0.304369
H 7.481246 2.150627 1.463984
H 10.335280 1.930069 -1.745036
H 10.498605 3.328377 0.054505
H 7.661208 -2.472166 2.470719
H 8.220012 -1.245678 1.351304
H 7.535138 -0.779232 4.299586
H 8.140162 0.465253 3.216752
H 3.930400 -0.663378 1.986443
H 2.962280 0.691778 2.105553
H 2.564508 -0.676771 2.953850
H 2.247360 -1.833690 0.746564
H -0.073200 -1.133974 0.591916
H 0.371515 -1.192879 2.297437
H 0.659344 1.295669 2.331873
H 0.269660 1.368579 0.602920
H -3.216904 1.247104 1.137083
H -1.900995 1.427459 0.001602
H 2.919493 2.330477 -1.410518
H -0.416037 3.950114 -3.333860
H -4.862836 4.738996 -2.758303
H -4.099637 3.715466 0.016982
H -5.702160 4.281657 -0.501596
H -3.394617 6.687793 -2.272302
H -4.704186 6.565745 -1.081736
H -3.066783 6.041816 -0.654863
H -5.020074 2.802559 -1.188636
H 5.374290 2.741288 -0.656667
H 7.155755 3.715716 -2.098811
H 6.387675 5.314238 -1.947659
H 7.071951 4.515103 -0.511596
H -3.746976 7.529675 1.290566
H -2.001532 7.568933 1.057884
H -3.139995 5.354540 1.798099
H -3.299450 6.072843 3.401934
H 0.240078 4.482125 2.232332
H -1.146139 4.389937 1.181048
H 1.115435 7.762088 4.796863
H -2.875979 9.408502 5.210042
H -1.154394 7.903167 3.881331
S -3.055659 -1.640217 -0.444861
H -4.941961 -2.656636 -1.539988
H -4.575696 -3.466336 -0.016678
H 4.586843 2.113024 2.171428
H 3.780991 1.912907 0.863523
H -0.222025 0.391457 -1.688393
H -1.694229 -0.074513 -1.753301
H 4.289705 0.870402 -2.073572
H 4.204614 4.018167 0.487111
H -4.093277 -0.761535 -0.279515

237
inactive_int1
C 0.131045 7.050339 7.018647
C -0.106645 8.449080 6.489329
O -0.948945 9.212295 6.959181
N 0.701241 8.817625 5.455712
C 0.567667 10.113423 4.829467
C -0.662975 10.317930 3.940266
O -0.961999 11.446155 3.560864
N -1.348303 9.196068 3.634990
C -2.512337 9.188678 2.776778
C -3.838863 9.208072 3.563810
O -4.912127 9.094129 2.974682
C -2.466107 8.060665 1.733062
C -2.217292 6.629044 2.263605
C -0.743684 6.321764 2.496214
N -0.093764 5.752593 1.478050
O -0.178906 6.625539 3.565501
N -3.754182 9.429224 4.891932
C -4.942583 9.678203 5.683858
C -4.455194 -5.190663 4.396060
C -4.154861 -3.719954 4.170842
O -4.548811 -3.137255 3.166908
N -3.477471 -3.111643 5.175759
C -3.098245 -1.711961 5.184063
C -1.752996 -1.628864 5.908535
O -1.521351 -2.335681 6.894515
C -4.144884 -0.835098 5.928061
C -3.683910 0.619363 6.073420
C -5.501165 -0.890875 5.221209
N -0.892962 -0.705656 5.445003
C 0.304692 -0.382194 6.164461
C 1.500868 -1.262410 5.796990
O 1.609080 -1.775698 4.684809
N 2.448048 -1.373275 6.745502
C 3.719981 -2.037837 6.506549
C -3.362021 -4.112152 -1.710996
C -2.206726 -4.840392 -1.024847
O -1.594839 -5.730782 -1.630660
C -4.185724 -3.121908 -0.891004
N -1.905448 -4.479918 0.238237
C -0.759339 -5.014622 0.955850
C -0.901727 -4.717790 2.445319
C 0.227088 -5.285029 3.314359
C 1.601004 -4.732160 2.921635
C -0.061316 -4.992851 4.789487
C 2.523281 -7.676775 -1.853794
C 2.488294 -6.295129 -1.215445
C 1.708566 -5.311948 -2.080270
C 1.816379 -3.888326 -1.570261
N 0.708719 -3.130444 -1.710948
O 2.853911 -3.451948 -1.067554
C 9.693847 -1.796678 2.830540
C 8.194694 -1.899298 2.548228
C 7.375094 -1.005870 3.476883
C 5.894666 -0.869952 3.108599
O 5.381084 -1.768042 2.385537
O 5.278589 0.135465 3.567281
C -2.898259 3.983904 -2.585058
C -1.670488 4.070836 -1.682520
O -1.521483 5.009418 -0.887226
C -4.239862 3.855985 -1.834008
C -4.208121 2.699531 -0.832026
C -4.668012 5.160480 -1.156882
N -0.787676 3.067466 -1.828249
C 0.454563 2.931654 -1.109547
C 1.678958 3.428478 -1.894261
O 1.632968 4.442048 -2.578023
N 2.782422 2.663410 -1.707539
C 4.122371 3.137584 -1.984069
C 4.935390 2.217846 -2.916167
O 5.674299 2.699598 -3.774879
C 4.902069 3.328030 -0.648812
C 6.227042 4.046996 -0.858442
O 4.105182 4.042774 0.279164
N 4.847087 0.887931 -2.673272
C 5.749012 -0.085767 -3.269529
C 6.482116 -0.885936 -2.180040
C 7.260199 0.006388 -1.242672
C 8.472463 0.575820 -1.631787
C 6.754097 0.339577 0.016804
C 9.165587 1.444332 -0.793800
C 7.427073 1.215490 0.862015
C 8.637483 1.765670 0.454462
O 9.274920 2.622750 1.317290
C -8.711113 -1.474723 -3.779924
C -8.841509 -0.350846 -2.742855
C -7.926131 -0.558359 -1.576972
C -6.584276 -0.328859 -1.450783
N -8.336269 -1.144366 -0.399153
C -7.283300 -1.271552 0.402039
N -6.208941 -0.769829 -0.205726
C -13.878955 -2.174883 -2.729032
C -13.660843 -3.227225 -1.641234
C -12.856875 -2.703878 -0.451881
C -11.376984 -2.412233 -0.747462
O -10.849615 -2.873197 -1.773948
O -10.783605 -1.704561 0.140612
N 2.820953 -1.029015 2.209255
C 1.868213 -1.275093 1.095992
C 2.234314 -0.429734 -0.140307

O 3.311465 0.225182 -0.120215
C 0.434446 -0.995721 1.553794
C 0.225016 0.480004 1.939077
C -1.243050 0.790614 2.128271
N -1.939075 1.085925 1.023384
O -1.772911 0.769646 3.251001
O 1.405903 -0.421814 -1.079283
O 3.721893 1.836170 2.044687
O -1.215084 0.267524 -1.732609
H -12.915741 -1.839099 -3.125068
H -7.678800 -1.558983 -4.138055
H 1.509164 -8.063127 -2.010173
H 10.270549 -2.457633 2.172918
H -0.557213 6.859336 7.842261
H -2.789091 3.135058 -3.269311
H -9.360649 -1.274409 -4.639224
H -14.410297 -1.300906 -2.330101
H -5.455864 10.584077 5.343149
H -4.642594 9.798945 6.726392
H 1.455389 10.301510 4.219183
H 5.187594 -0.771029 -3.916352
H 0.578752 1.876698 -0.853657
H -4.010855 -4.903814 -2.107036
H -0.708005 -6.093020 0.772371
H -4.288950 -5.726795 3.458890
H 3.552355 -3.072731 6.195294
H 4.296755 -2.025825 7.432928
H 9.927144 -2.068737 3.868567
H 3.065474 -8.395937 -1.228673
H -14.469419 -2.576620 -3.561306
H -9.023275 -2.420950 -3.331632
H -2.932104 -3.602057 -2.582532
H 0.161441 -4.572703 0.554520
H 3.020445 -7.643116 -2.831364
H 10.047145 -0.770933 2.668399
H -5.641372 8.840590 5.600863
H 4.281189 -1.521135 5.720400
H 0.406038 3.507078 -0.180049
H -2.902135 4.894838 -3.194961
H 6.452814 0.473652 -3.890443
H 0.521655 10.884902 5.601289
H -3.848474 -5.643766 5.186278
H -5.512922 -5.295379 4.658339
H 4.025235 4.097651 -2.494344
H -2.514535 10.143375 2.238192
H -0.035307 6.318761 6.219756
H 1.163123 6.943113 7.367589
H -14.635275 -3.589163 -1.283414
H -13.132801 -4.084702 -2.071673
H -13.309090 -1.792324 -0.042056
H -12.870153 -3.438797 0.364321
H -8.614665 0.618747 -3.201874
H -9.873319 -0.314057 -2.381369
H -9.384812 -1.439402 -0.176053
H -7.283301 -1.718939 1.383293
H -5.859944 0.085721 -2.134194
H -2.287627 -3.590392 0.563054
H -0.960839 -3.630046 2.597470
H -1.862238 -5.125490 2.787771
H 0.241681 -6.376867 3.172989
H 0.697354 -5.443968 5.439952
H -0.058191 -3.913157 4.976547
H -1.041817 -5.383382 5.089712
H 2.382457 -5.119163 3.585460
H 1.883690 -4.987517 1.895189
H 1.606553 -3.641623 3.020490
H -3.128500 -3.650863 5.956653
H -3.009046 -1.368000 4.147867
H -5.891899 -1.910145 5.167653
H -6.230092 -0.274652 5.760448
H -5.418863 -0.513286 4.196444
H -4.254901 -1.270903 6.931485
H -3.440396 1.051677 5.096655
H -4.482032 1.218571 6.525669
H -2.798895 0.709981 6.711291
H -1.145964 -0.149321 4.618978
H 0.109856 -0.438475 7.241272
H 0.582754 0.649077 5.918158
H 2.276617 -0.953987 7.645207
H 3.501836 -5.913361 -1.058367
H 2.016926 -6.354455 -0.225639
H 2.122791 -5.311859 -3.099089
H 0.658208 -5.616209 -2.151377
H -0.136687 -3.514660 -2.103069
H 0.779045 -2.133536 -1.510710
H 7.150952 -1.602757 -2.673752
H 5.751461 -1.473817 -1.611872
H 8.884109 0.344643 -2.611845
H 5.811011 -0.092479 0.341454
H 7.010873 1.483853 1.828132
H 10.110587 1.877609 -1.117407
H 10.033444 3.013377 0.868502
H 7.853684 -2.935864 2.642894
H 8.000329 -1.609528 1.510623
H 7.421050 -1.387390 4.508000
H 7.800328 0.003511 3.515169
H 3.819221 -1.394827 2.082772
H 2.941429 -0.023347 2.389914
H 2.475456 -1.439164 3.088574
H 1.978011 -2.323757 0.803321
H -0.243606 -1.276237 0.741887
H 0.193151 -1.631018 2.413471
H 0.741130 0.716497 2.874252
H 0.637349 1.133784 1.163818
H -2.946288 1.058196 1.107270
H -1.568326 0.918744 0.084593
H 2.708030 1.838198 -1.119020
H -1.075713 2.237218 -2.334398
H -4.988092 3.618359 -2.603344
H -3.540481 2.946940 0.001444
H -5.205882 2.508763 -0.420334
H -4.738603 5.978558 -1.882906
H -5.647376 5.042364 -0.679045
H -3.942266 5.457361 -0.394549
H -3.845173 1.770314 -1.289714
H 5.110494 2.329228 -0.248433
H 6.877554 3.494032 -1.542685
H 6.050555 5.046016 -1.271958
H 6.735475 4.148777 0.105242
H -3.431139 8.072847 1.220131
H -1.700809 8.303936 0.988087
H -2.613895 5.921142 1.529869
H -2.751580 6.467699 3.206269
H 0.900442 5.590406 1.569741
H -0.552886 5.494985 0.598096
H 1.170057 8.079808 4.945251
H -2.843039 9.523327 5.321864
H -0.948267 8.293562 3.895884
S -3.270458 -1.636874 -0.305015
H -5.028675 -2.806124 -1.516765
H -4.610849 -3.631667 -0.017550
H 4.401788 1.420537 2.622137
H 3.734864 1.302143 1.222774
H -1.853334 -0.467241 -1.570962
H -0.321340 -0.097742 -1.588613
H 4.244409 0.564486 -1.918283
H 3.942746 3.453623 1.039450
H -5.197796 -0.896560 0.061476

237
inactive_int2
C 0.131165 7.050370 7.018470
C -0.102054 8.351315 6.283019
O -0.896618 9.209041 6.663314
N 0.643867 8.516026 5.155048
C 0.527915 9.706471 4.344624
C -0.758310 9.850746 3.528422
O -1.019839 10.922665 2.991378
N -1.534233 8.748535 3.461165
C -2.762631 8.707276 2.702018
C -4.020799 8.896339 3.571242
O -5.139849 8.749481 3.082189
C -2.863167 7.467837 1.803020
C -2.839361 6.083820 2.489950
C -1.446719 5.575722 2.833925
N -0.951410 4.601866 2.059055
O -0.800223 6.047686 3.786544
N -3.824928 9.289024 4.845935
C -4.942446 9.678266 5.683655
C -4.455197 -5.190609 4.396090
C -4.126522 -3.868690 3.733519
O -4.411265 -3.661331 2.559963
N -3.471611 -2.973513 4.517138
C -2.819755 -1.785794 3.998857
C -1.514503 -1.609398 4.765292
O -1.446301 -1.801560 5.980008
C -3.694397 -0.509547 4.157217
C -2.898479 0.781064 3.929242
C -4.878014 -0.574028 3.188598
N -0.470585 -1.144779 4.040313
C 0.695552 -0.590721 4.685372
C 1.779076 -1.619439 5.024092
O 1.997531 -2.587558 4.295975
N 2.493745 -1.344591 6.135368
C 3.720007 -2.037863 6.506489
C -3.361932 -4.112146 -1.711008
C -2.205534 -4.837720 -1.012301
O -1.699971 -5.838661 -1.534776
C -3.486774 -2.616992 -1.428860
N -1.818823 -4.375720 0.194093
C -0.759550 -5.014705 0.955876
C -0.882651 -4.671043 2.438214
C -0.130176 -5.631076 3.366222
C 1.363009 -5.713919 3.035935
C -0.339202 -5.214566 4.824419
C 2.523239 -7.676897 -1.853779
C 2.765365 -6.205279 -1.524689
C 1.972467 -5.260592 -2.428137
C 2.467450 -3.821271 -2.327317
N 1.568034 -2.852470 -2.614030
O 3.637304 -3.559750 -2.042591
C 9.693822 -1.796771 2.830427
C 8.203750 -1.791067 2.445470
C 7.344546 -0.986873 3.422333
C 5.844901 -0.864538 3.094861
O 5.360083 -1.593919 2.187780
O 5.199437 -0.023651 3.785442
C -2.898105 3.983788 -2.585189
C -1.743398 3.637727 -1.650601
O -1.898198 3.409312 -0.437835
C -4.103112 4.692938 -1.949818
C -4.894439 3.792904 -0.994317
C -3.696858 6.010412 -1.287529
N -0.539893 3.514627 -2.234004
C 0.650936 3.106512 -1.525163
C 1.883530 3.426740 -2.374094
O 1.792702 4.148142 -3.367967
N 3.014255 2.882163 -1.898486
C 4.342692 3.322904 -2.272860
C 5.180507 2.291594 -3.049633
O 6.138087 2.683439 -3.719254
C 5.074326 3.717953 -0.970931
C 6.537934 4.098475 -1.135463
O 4.321054 4.806643 -0.431057
N 4.860622 0.995363 -2.875857
C 5.749017 -0.085929 -3.269660
C 6.234728 -0.873384 -2.040120
C 6.993511 -0.018171 -1.053550
C 8.348992 0.258384 -1.234159
C 6.359368 0.543301 0.058877
C 9.054669 1.057209 -0.337558
C 7.046043 1.345178 0.963705
C 8.398530 1.599569 0.764493
O 9.045547 2.391896 1.682840
C -8.711143 -1.474773 -3.779876
C -8.173203 -0.108960 -3.325104
C -7.212228 -0.197028 -2.181146
C -5.880718 0.099348 -2.083268
N -7.610484 -0.648067 -0.940096
C -6.562188 -0.626389 -0.121110
N -5.498547 -0.170875 -0.786057
C -13.879015 -2.174840 -2.729068
C -13.585093 -2.229854 -1.225803
C -12.510289 -1.247044 -0.747179
C -11.043547 -1.662732 -0.988886
O -10.793085 -2.755111 -1.523645
O -10.184478 -0.801561 -0.586452
N 2.692842 -1.315927 1.780213
C 2.158006 -1.389667 0.393096
C 2.550809 -0.128772 -0.398620
O 2.874277 0.910506 0.243849
C 0.625676 -1.626563 0.373075
C -0.277640 -0.408545 0.139030
C -1.773524 -0.806143 0.118836
N -2.712573 0.332842 -0.021716
O -2.131382 -1.608966 1.051306
O 2.477663 -0.221234 -1.644836
O 2.885520 1.225924 3.054169
O -3.060613 0.788814 -3.464402
H -12.972308 -2.405452 -3.297558
H -7.890137 -2.118162 -4.116218
H 1.460583 -7.932051 -1.766272

H 10.283458 -2.382559 2.116002
H -0.418681 7.069428 7.959643
H -3.214800 3.021903 -3.012077
H -9.404420 -1.339719 -4.617352
H -14.225981 -1.176417 -3.026112
H -5.463224 10.543932 5.260478
H -4.558937 9.931018 6.673755
H 1.371424 9.743033 3.649905
H 5.220009 -0.766416 -3.946053
H 0.624595 2.039641 -1.280579
H -4.290357 -4.608836 -1.399809
H -0.844221 -6.096352 0.803441
H -3.717668 -5.929336 4.062943
H 3.634213 -3.080089 6.195217
H 3.840786 -1.990371 7.591496
H 9.847047 -2.225910 3.829148
H 3.085990 -8.330517 -1.177569
H -14.653338 -2.899179 -3.008910
H -9.250052 -1.985696 -2.975558
H -3.233632 -4.300514 -2.780437
H 0.224867 -4.719290 0.563059
H 2.837485 -7.904317 -2.880235
H 10.094420 -0.775270 2.842157
H -5.661818 8.858538 5.770183
H 4.583163 -1.585052 6.005631
H 0.735857 3.654830 -0.580071
H -2.509299 4.589500 -3.414224
H 6.585874 0.364401 -3.808226
H 0.584761 10.589055 4.986147
H -4.432956 -5.147980 5.489700
H -5.439391 -5.523169 4.060377
H 4.244645 4.203340 -2.912533
H -2.754309 9.587319 2.048490
H -0.225975 6.216657 6.402832
H 1.196763 6.896030 7.214721
H -14.516291 -2.027581 -0.677118
H -13.268953 -3.244635 -0.961338
H -12.655431 -0.258163 -1.202637
H -12.607010 -1.085626 0.334028
H -7.664712 0.398977 -4.152409
H -9.015175 0.524281 -3.021544
H -8.656936 -0.869080 -0.723567
H -6.562014 -0.938557 0.911089
H -5.165551 0.460137 -2.810894
H -2.132432 -3.462743 0.533257
H -0.537452 -3.644758 2.621850
H -1.947290 -4.687702 2.694745
H -0.566956 -6.632554 3.225733
H 0.128385 -5.928618 5.513404
H 0.104147 -4.229383 5.006507
H -1.406878 -5.153396 5.070278
H 1.874357 -6.400552 3.721617
H 1.535378 -6.076376 2.016147
H 1.832817 -4.730370 3.142455
H -3.229127 -3.219449 5.467576
H -2.626866 -1.932525 2.931219
H -5.501459 -1.453047 3.371932
H -5.501180 0.323760 3.283252
H -4.499277 -0.645880 2.162117
H -4.069272 -0.502898 5.189908
H -2.382348 0.758119 2.962268
H -3.573329 1.644027 3.928911
H -2.144368 0.949373 4.704870
H -0.602309 -1.051166 3.039861
H 0.371526 -0.072286 5.594150
H 1.163400 0.150434 4.024670
H 2.296595 -0.472156 6.601233
H 3.828841 -5.965322 -1.617457
H 2.495267 -6.009717 -0.478784
H 2.099806 -5.562252 -3.478156
H 0.897804 -5.325673 -2.214058
H 0.578171 -3.053382 -2.668511
H 1.851776 -1.884116 -2.441688
H 6.869942 -1.694806 -2.394130
H 5.371378 -1.343796 -1.556428
H 8.870255 -0.160075 -2.092386
H 5.307900 0.342569 0.236581
H 6.543971 1.751096 1.836940
H 10.113250 1.255294 -0.498568
H 9.968865 2.482563 1.420944
H 7.825835 -2.817993 2.390182
H 8.088176 -1.372470 1.439599
H 7.413713 -1.419471 4.431702
H 7.731185 0.035884 3.513294
H 3.758788 -1.422895 1.852082
H 2.505097 -0.397765 2.219131
H 2.300672 -2.034122 2.404465
H 2.658612 -2.238917 -0.078139
H 0.409200 -2.350411 -0.414948
H 0.331263 -2.120361 1.306000
H -0.153843 0.336615 0.941887
H -0.002470 0.085980 -0.799131
H -2.744362 0.803618 0.883204
H -2.369247 1.029769 -0.682305
H 2.939882 2.268189 -1.078614
H -0.383099 3.827399 -3.184848
H -4.769625 4.936233 -2.789443
H -4.296301 3.528674 -0.118643
H -5.804947 4.302099 -0.657197
H -3.198532 6.680228 -1.998616
H -4.570958 6.534285 -0.884232
H -3.003604 5.823649 -0.461553
H -5.193044 2.861954 -1.489398
H 5.003809 2.859135 -0.289258
H 7.135407 3.246858 -1.468190
H 6.646708 4.908579 -1.862480
H 6.933404 4.427762 -0.166419
H -3.813034 7.553626 1.270484
H -2.066065 7.521866 1.053031
H -3.333695 5.364655 1.828968
H -3.418259 6.116849 3.419868
H -0.020826 4.263624 2.264792
H -1.417349 4.217749 1.235876
H 1.104818 7.702893 4.770881
H -2.880228 9.392237 5.195292
H -1.192245 7.864747 3.841923
S -1.968965 -1.668244 -1.775605
H -4.515583 -0.063356 -0.430509
H -4.311194 -2.214261 -2.023230
H -3.719566 -2.442664 -0.369741
H 3.751247 0.838697 3.351995
H 3.005581 1.427062 2.107097
H -2.536850 0.752741 -4.273523
H -2.729179 0.026170 -2.927327
H 3.987358 0.737307 -2.411172
H 4.625552 4.954470 0.471503

237
inactive_int3
C 0.131251 7.050732 7.017989
C -0.106665 8.352616 6.285212
O -0.906703 9.204916 6.665603
N 0.641352 8.524995 5.159747
C 0.530830 9.722162 4.358413
C -0.748719 9.874220 3.533189
O -1.002078 10.949839 2.999965
N -1.528013 8.775210 3.454027
C -2.755718 8.745630 2.692982
C -4.015122 8.929302 3.561723
O -5.132531 8.793387 3.066562
C -2.861170 7.514956 1.782350
C -2.851314 6.125707 2.459222
C -1.463467 5.606065 2.802817
N -0.970058 4.636543 2.018284
O -0.814414 6.063837 3.759053
N -3.822177 9.301570 4.842990
C -4.942370 9.678634 5.683128
C -4.455205 -5.190346 4.396164
C -4.023554 -3.925819 3.681890
O -4.186046 -3.796376 2.473766
N -3.417125 -2.992623 4.458329
C -2.765893 -1.813477 3.922598
C -1.482543 -1.596066 4.716584
O -1.430239 -1.791031 5.930856
C -3.670609 -0.551857 4.011707
C -2.914786 0.732810 3.652557
C -4.883073 -0.718859 3.093048
N -0.434769 -1.103917 4.012728
C 0.718046 -0.545963 4.679170
C 1.808147 -1.572290 5.004011
O 2.053559 -2.510318 4.245600
N 2.499156 -1.333313 6.137421
C 3.719967 -2.037511 6.506355
C -3.361941 -4.112141 -1.711012
C -2.169788 -4.798849 -1.032423
O -1.536618 -5.682760 -1.620397
C -3.474274 -2.615106 -1.444230
N -1.871573 -4.422593 0.228936
C -0.759532 -5.014624 0.955920
C -0.819975 -4.658066 2.436557
C 0.005447 -5.587988 3.332716
C 1.486573 -5.615013 2.945361
C -0.168683 -5.181981 4.798390
C 2.523192 -7.676948 -1.853666
C 2.797303 -6.211588 -1.523888
C 2.019793 -5.256142 -2.427424
C 2.503671 -3.817248 -2.298174
N 1.613721 -2.854323 -2.636502
O 3.652204 -3.544978 -1.946406
C 9.693896 -1.796664 2.830265
C 8.211606 -1.748583 2.421983
C 7.357358 -0.932037 3.392685
C 5.863534 -0.793310 3.050845
O 5.378637 -1.534529 2.152298
O 5.221613 0.067383 3.718691
C -2.898139 3.983644 -2.585667
C -1.723254 3.679169 -1.663092
O -1.868956 3.344461 -0.472228
C -4.100736 4.688905 -1.938143
C -4.855979 3.815072 -0.930567
C -3.692094 6.031610 -1.328944
N -0.500188 3.714898 -2.217007
C 0.690611 3.292109 -1.515181
C 1.914946 3.538062 -2.400804
O 1.829267 4.263725 -3.391663
N 3.030841 2.935605 -1.960062
C 4.367888 3.353280 -2.337755
C 5.200545 2.301050 -3.091401
O 6.166543 2.674574 -3.758634
C 5.097580 3.764328 -1.039325
C 6.558400 4.152051 -1.209543
O 4.336255 4.852792 -0.510488
N 4.868236 1.010452 -2.900435
C 5.749019 -0.085985 -3.269904
C 6.236683 -0.845696 -2.024106
C 7.007572 0.027131 -1.062768
C 8.361733 0.296381 -1.263191
C 6.387457 0.609715 0.046519
C 9.079600 1.107833 -0.388021
C 7.086741 1.423010 0.931305
C 8.437797 1.669629 0.712843
O 9.096976 2.474459 1.610890
C -8.711107 -1.474775 -3.779875
C -8.048111 -0.112350 -3.525757
C -7.115665 -0.103584 -2.354177
C -5.768756 0.138432 -2.230775
N -7.557196 -0.358197 -1.080774
C -6.494947 -0.261836 -0.255884
N -5.382624 0.040013 -0.908504
C -13.879042 -2.174842 -2.729064
C -13.655941 -1.975679 -1.226748
C -12.588333 -0.933331 -0.878413
C -11.114315 -1.379370 -1.039081
O -10.874450 -2.545874 -1.407408
O -10.259478 -0.477599 -0.761587
N 2.732403 -1.257357 1.738041
C 2.206260 -1.346647 0.351195
C 2.576084 -0.085866 -0.451302
O 2.894828 0.960603 0.178622
C 0.674547 -1.595153 0.326231
C -0.213493 -0.388370 -0.007503
C -1.695838 -0.804858 -0.058284
N -2.555198 0.477125 -0.344707
O -2.151359 -1.426184 0.953906
O 2.478334 -0.186861 -1.696874
O 2.891108 1.285335 3.000165
O -1.743108 1.065942 -3.143466
H -12.947901 -2.501302 -3.202336
H -7.952376 -2.234676 -4.001582
H 1.455304 -7.908265 -1.764422
H 10.279889 -2.389520 2.118753
H -0.417482 7.066852 7.959851
H -3.201396 3.010654 -2.991703
H -9.388852 -1.408835 -4.639160
H -14.198585 -1.238899 -3.206690
H -5.456317 10.556249 5.276586
H -4.562961 9.907184 6.680653
H 1.379913 9.765460 3.670943
H 5.212328 -0.779249 -3.926894
H 0.622626 2.237552 -1.223802
H -4.281388 -4.600545 -1.363833
H -0.810999 -6.101864 0.823415

H -3.729960 -5.977722 4.162737
H 3.629392 -3.077981 6.190219
H 3.838918 -1.995252 7.591471
H 9.819682 -2.241185 3.826029
H 3.072564 -8.343680 -1.179330
H -14.649170 -2.932249 -2.921524
H -9.292174 -1.812031 -2.915775
H -3.260411 -4.317823 -2.779679
H 0.193507 -4.694730 0.509675
H 2.830009 -7.910699 -2.880898
H 10.121259 -0.786447 2.860794
H -5.666209 8.860923 5.747117
H 4.588081 -1.588646 6.010856
H 0.823843 3.871589 -0.593596
H -2.536634 4.576006 -3.435798
H 6.586002 0.345178 -3.823713
H 0.582017 10.599207 5.008057
H -4.512397 -5.079789 5.483578
H -5.426354 -5.501934 4.006748
H 4.280869 4.223049 -2.993196
H -2.742763 9.631635 2.047831
H -0.225233 6.216990 6.402058
H 1.197470 6.898611 7.212615
H -14.608519 -1.678949 -0.762594
H -13.363180 -2.932881 -0.781710
H -12.727626 -0.021411 -1.475027
H -12.702351 -0.618263 0.166926
H -7.484945 0.209385 -4.410637
H -8.833150 0.635002 -3.355088
H -8.570728 -0.533434 -0.850276
H -6.567133 -0.424623 0.809445
H -5.054320 0.377230 -3.008933
H -2.316790 -3.605903 0.640585
H -0.497225 -3.619616 2.594244
H -1.866305 -4.709848 2.749888
H -0.398867 -6.604661 3.206016
H 0.350263 -5.878458 5.468143
H 0.241796 -4.180846 4.968358
H -1.228958 -5.162563 5.080738
H 2.048698 -6.284560 3.607455
H 1.632771 -5.967935 1.918113
H 1.924994 -4.615946 3.038660
H -3.257905 -3.172899 5.440221
H -2.544042 -1.993073 2.867389
H -5.484896 -1.588966 3.367468
H -5.523466 0.169888 3.145637
H -4.549450 -0.859486 2.059026
H -4.011686 -0.473803 5.053801
H -2.449977 0.629946 2.665099
H -3.609975 1.578452 3.614864
H -2.130028 0.978255 4.375536
H -0.564051 -0.972391 3.017310
H 0.381193 -0.046665 5.593867
H 1.184414 0.208742 4.032843
H 2.272929 -0.492962 6.646532
H 3.865555 -5.993042 -1.615592
H 2.531336 -6.010232 -0.478149
H 2.164359 -5.542705 -3.479516
H 0.941718 -5.326307 -2.231700
H 0.630457 -3.069397 -2.732113
H 1.884529 -1.883012 -2.462147
H 6.863832 -1.680077 -2.361657
H 5.374067 -1.299093 -1.523205
H 8.872304 -0.138172 -2.119777
H 5.337188 0.415557 0.237902
H 6.595843 1.843827 1.803789
H 10.136731 1.300124 -0.564392
H 10.018189 2.557138 1.338999
H 7.807818 -2.764454 2.350899
H 8.122756 -1.317526 1.418475
H 7.411769 -1.366835 4.402042
H 7.756518 0.085437 3.487443
H 3.801894 -1.360441 1.816717
H 2.537512 -0.337201 2.170622
H 2.343563 -1.972013 2.368794
H 2.717956 -2.191668 -0.116030
H 0.469992 -2.372234 -0.412906
H 0.361750 -2.018352 1.287278
H -0.113512 0.395268 0.759431
H 0.097872 0.050008 -0.962525
H -2.471321 1.139095 0.429752
H -2.256100 0.972038 -1.195762
H 2.951394 2.326394 -1.137039
H -0.342741 4.089673 -3.145637
H -4.791113 4.895995 -2.767524
H -4.252367 3.632779 -0.037464
H -5.785110 4.309650 -0.625247
H -3.224542 6.687972 -2.072740
H -4.560547 6.554605 -0.913228
H -2.972115 5.880791 -0.516873
H -5.120159 2.840855 -1.356397
H 5.032725 2.911722 -0.349215
H 7.158248 3.302541 -1.543005
H 6.660306 4.960798 -1.939146
H 6.956708 4.485450 -0.242988
H -3.808160 7.611701 1.246474
H -2.059949 7.569327 1.036676
H -3.350541 5.415475 1.792166
H -3.432696 6.156669 3.387538
H -0.042716 4.290151 2.224680
H -1.440922 4.252616 1.199408
H 1.113756 7.718154 4.776851
H -2.879016 9.400314 5.197494
H -1.193393 7.889426 3.835357
S -1.908085 -1.753460 -1.789394
H -3.578751 0.227824 -0.452779
H -4.275287 -2.178064 -2.042973
H -3.706418 -2.408646 -0.392118
H 3.761616 0.909259 3.300463
H 3.018754 1.500095 2.057886
H -0.814742 1.202157 -3.371051
H -1.865646 0.094716 -3.089810
H 3.988459 0.766703 -2.441780
H 4.677247 5.048483 0.369715

237
inactive_int4
C 0.130775 7.049461 7.020279
C -0.104297 8.394222 6.369385
O -0.926000 9.210270 6.781898
N 0.669630 8.646847 5.276048
C 0.541162 9.877301 4.529677
C -0.707862 10.021196 3.656155
O -0.962223 11.103642 3.136606
N -1.459290 8.908855 3.524720
C -2.662706 8.865086 2.726024
C -3.949779 9.003043 3.563825
O -5.050603 8.864226 3.034143
C -2.707347 7.649077 1.788110
C -2.684193 6.245516 2.432878
C -1.307233 5.761208 2.864516
N -0.741938 4.811852 2.109377
O -0.734952 6.222554 3.869176
N -3.797598 9.342099 4.860435
C -4.942653 9.677610 5.685125
C -4.455635 -5.191660 4.396853
C -3.956853 -3.963765 3.662159
O -4.013554 -3.901869 2.434684
N -3.427679 -2.988514 4.434024
C -2.771556 -1.811063 3.897966
C -1.533553 -1.554045 4.753272
O -1.534981 -1.755962 5.967015
C -3.699381 -0.563964 3.883284
C -2.975811 0.685060 3.363858
C -4.935080 -0.835284 3.022686
N -0.464677 -1.034708 4.102845
C 0.668369 -0.496692 4.815939
C 1.753154 -1.535680 5.109159
O 1.946607 -2.490252 4.355392
N 2.501763 -1.299529 6.203598
C 3.719155 -2.038730 6.508553
C -3.362515 -4.111893 -1.711202
C -2.116990 -4.737887 -1.062219
O -1.270135 -5.319799 -1.747504
C -3.476654 -2.612765 -1.414891
N -1.973162 -4.584322 0.272244
C -0.759537 -5.015371 0.957246
C -0.822501 -4.771369 2.457877
C 0.197529 -5.602314 3.246293
C 1.634809 -5.383194 2.766093
C 0.061284 -5.303016 4.741199
C 2.523368 -7.677438 -1.852439
C 2.991815 -6.256580 -1.554080
C 2.203157 -5.214325 -2.340411
C 2.731646 -3.804391 -2.124247
N 2.004835 -2.804936 -2.681171
O 3.765544 -3.581707 -1.493429
C 9.693669 -1.797687 2.832888
C 8.195220 -1.718203 2.523618
C 7.436620 -0.833362 3.511523
C 5.932505 -0.693884 3.248294
O 5.418837 -1.458116 2.382426
O 5.309544 0.172598 3.921938
C -2.895994 3.985188 -2.580297
C -1.708400 3.796257 -1.643619
O -1.790390 3.906577 -0.417297
C -4.089992 4.773533 -2.019389
C -4.842975 4.034589 -0.907167
C -3.694467 6.189349 -1.588370
N -0.550970 3.395316 -2.240619
C 0.662938 3.166800 -1.481194
C 1.883238 3.403481 -2.374826
O 1.777705 4.055516 -3.412748
N 3.023884 2.891364 -1.882765
C 4.345894 3.329512 -2.289823
C 5.176724 2.296298 -3.073653
O 6.110722 2.687651 -3.775007
C 5.100010 3.739012 -1.005895
C 6.558882 4.122133 -1.203349
O 4.351871 4.829149 -0.463155
N 4.874899 1.001405 -2.866289
C 5.749278 -0.086373 -3.268039
C 6.267923 -0.860881 -2.042971
C 7.036588 0.009889 -1.077791
C 8.378905 0.319908 -1.298715
C 6.423101 0.556277 0.053156
C 9.090219 1.137299 -0.423910
C 7.114347 1.378760 0.935564
C 8.453496 1.666886 0.695940
O 9.105533 2.478106 1.592522
C -8.710893 -1.475036 -3.779647
C -8.168095 -0.131750 -3.271431
C -7.330396 -0.259751 -2.038481
C -6.006853 0.010815 -1.779665
N -7.840832 -0.729733 -0.856376
C -6.834979 -0.721207 0.044576
N -5.700222 -0.280062 -0.467776
C -13.878683 -2.174830 -2.729103
C -13.689394 -2.730644 -1.316911
C -12.718665 -1.905508 -0.472599
C -11.233124 -1.994218 -0.893641
O -10.876020 -2.935418 -1.631345
O -10.490691 -1.083859 -0.409492
N 2.840225 -1.213449 1.948877
C 2.460443 -1.379989 0.528343
C 2.729326 -0.092067 -0.267302
O 2.940706 0.979708 0.360563
C 0.988396 -1.820670 0.376549
C -0.018929 -0.703484 0.042310
C -1.385193 -1.333735 -0.046994
N -2.888996 1.061204 -0.221691
O -1.994825 -1.683208 0.936742
O 2.666739 -0.212956 -1.514354
O 2.926417 1.338159 3.207150
O -2.055162 1.052849 -3.537443
H -12.919080 -2.162264 -3.255010
H -7.884037 -2.152739 -4.023508
H 1.458959 -7.798063 -1.619002
H 10.213058 -2.439634 2.112213
H -0.401851 7.015754 7.971034
H -3.212635 2.978005 -2.880146
H -9.307762 -1.325274 -4.687667
H -14.266958 -1.148073 -2.698966
H -5.462109 10.557517 5.290665
H -4.588910 9.885074 6.696591
H 1.415701 9.991896 3.883527
H 5.203331 -0.773456 -3.924901
H 0.675482 2.159261 -1.051483
H -4.270689 -4.604958 -1.344025
H -0.614608 -6.086752 0.761277
H -3.755601 -6.012191 4.206112
H 3.587681 -3.070763 6.180668
H 3.886723 -2.017294 7.587945
H 9.874770 -2.202747 3.837111
H 3.085521 -8.412017 -1.264702
H -14.581743 -2.784295 -3.310907
H -9.345528 -1.962841 -3.030730
H -3.277302 -4.299779 -2.783358
H 0.103170 -4.510363 0.507501
H 2.657875 -7.923027 -2.913310
H 10.154284 -0.802600 2.789006
H -5.653938 8.846807 5.711168

H 4.576228 -1.601584 5.984256
H 0.724448 3.875974 -0.647242
H -2.534325 4.465357 -3.499766
H 6.573602 0.353343 -3.834459
H 0.527921 10.719973 5.224991
H -4.543740 -5.048629 5.477908
H -5.425795 -5.477462 3.984858
H 4.233157 4.203171 -2.936370
H -2.653242 9.763892 2.098789
H -0.248979 6.262165 6.359002
H 1.197803 6.870685 7.185339
H -14.665131 -2.775113 -0.810135
H -13.307638 -3.755002 -1.384075
H -13.009178 -0.847392 -0.464932
H -12.762107 -2.236532 0.574312
H -7.560725 0.352607 -4.046176
H -9.012961 0.537960 -3.064530
H -8.851363 -0.978025 -0.687542
H -6.972842 -1.048857 1.065492
H -5.259813 0.406933 -2.458497
H -2.683521 -4.107636 0.821141
H -0.682079 -3.703816 2.679042
H -1.823676 -5.035940 2.810456
H -0.055269 -6.661751 3.082943
H 0.726209 -5.941651 5.334900
H 0.324163 -4.260094 4.946936
H -0.967674 -5.469829 5.084998
H 2.337606 -5.977350 3.361479
H 1.763927 -5.672903 1.717274
H 1.921322 -4.333447 2.878742
H -3.354661 -3.114367 5.434466
H -2.490413 -2.033416 2.869075
H -5.506691 -1.693471 3.387868
H -5.595372 0.039039 3.029651
H -4.648844 -1.030863 1.983850
H -4.015173 -0.385000 4.921432
H -2.578745 0.512785 2.355424
H -3.684838 1.517185 3.297031
H -2.156564 1.002304 4.017374
H -0.538758 -0.903399 3.103716
H 0.311711 -0.044807 5.746932
H 1.141818 0.288954 4.212337
H 2.339130 -0.436923 6.700675
H 4.057740 -6.150478 -1.780318
H 2.891638 -6.046821 -0.482766
H 2.252887 -5.431317 -3.417194
H 1.137039 -5.250820 -2.074369
H 1.074261 -2.991846 -3.026659
H 2.245242 -1.844811 -2.418110
H 6.905650 -1.677305 -2.404440
H 5.421061 -1.337401 -1.536347
H 8.884685 -0.085192 -2.172354
H 5.384029 0.329178 0.264784
H 6.626594 1.778017 1.819977
H 10.137967 1.361862 -0.616946
H 10.015447 2.602242 1.299082
H 7.755702 -2.721370 2.526359
H 8.049302 -1.328069 1.510420
H 7.550574 -1.217767 4.535936
H 7.859233 0.179026 3.526379
H 3.914477 -1.287860 2.104454
H 2.586749 -0.284593 2.324258
H 2.423185 -1.920074 2.568607
H 3.106173 -2.160643 0.115345
H 0.939330 -2.562897 -0.423487
H 0.672073 -2.331837 1.294477
H -0.060380 0.069904 0.816736
H 0.254156 -0.225586 -0.902418
H -2.956470 1.956744 0.255306
H -2.928622 1.285193 -1.214867
H 2.963489 2.324885 -1.030522
H -0.385587 3.657344 -3.208806
H -4.783598 4.868913 -2.866418
H -4.243915 3.985180 0.006181
H -5.776912 4.558135 -0.673641
H -3.210260 6.736780 -2.406321
H -4.576403 6.759886 -1.275668
H -2.997717 6.156695 -0.744434
H -5.096981 3.008922 -1.197385
H 5.047199 2.886523 -0.314060
H 7.153559 3.266551 -1.531118
H 6.652862 4.919749 -1.945923
H 6.970936 4.468462 -0.247111
H -3.638796 7.731368 1.222210
H -1.883859 7.738071 1.070243
H -3.103920 5.536661 1.711976
H -3.331197 6.229703 3.317380
H 0.175413 4.480811 2.375184
H -1.154796 4.447777 1.250657
H 1.149345 7.864738 4.852407
H -2.865563 9.439398 5.242803
H -1.126636 8.023726 3.910230
S -1.903400 -1.738431 -1.708910
H -3.761902 0.552130 -0.038620
H -4.237807 -2.140535 -2.038967
H -3.750533 -2.405475 -0.376234
H 3.803133 0.983109 3.508594
H 3.055476 1.568364 2.269494
H -1.311901 1.512055 -3.118736
H -2.077613 0.178856 -3.117676
H 4.023904 0.748650 -2.359842
H 4.708974 5.024664 0.410709

237
inactive_TS1
C 0.131080 7.050210 7.018690
C -0.098006 8.369307 6.314821
O -0.899361 9.214310 6.710175
N 0.655039 8.562299 5.196239
C 0.516635 9.756343 4.394340
C -0.765224 9.873218 3.565732
O -1.045812 10.939371 3.026374
N -1.515629 8.754135 3.492680
C -2.738401 8.679761 2.726547
C -4.004631 8.856074 3.589130
O -5.119137 8.683571 3.098832
C -2.802896 7.432424 1.832393
C -2.709022 6.049694 2.516406
C -1.294305 5.617558 2.877244
N -0.719327 4.716146 2.073169
O -0.701639 6.088232 3.866382
N -3.820067 9.277843 4.857221
C -4.942531 9.678109 5.683928
C -4.455222 -5.190746 4.396013
C -3.938914 -3.815628 4.018422
O -3.964194 -3.420974 2.856836
N -3.501040 -3.071640 5.059837
C -3.045778 -1.698031 4.980860
C -1.813807 -1.588977 5.879212
O -1.746036 -2.216125 6.941024
C -4.172576 -0.734253 5.451987
C -3.681251 0.678044 5.781936
C -5.278523 -0.685741 4.394320
N -0.857294 -0.745606 5.446310
C 0.272465 -0.409242 6.268174
C 1.563536 -1.081786 5.803015
O 1.825374 -1.191349 4.607954
N 2.412547 -1.464000 6.777981
C 3.719962 -2.037982 6.506491
C -3.361940 -4.112166 -1.710988
C -2.139640 -4.739866 -1.048893
O -1.344873 -5.397030 -1.734182
C -4.182237 -3.117102 -0.893629
N -1.972843 -4.577521 0.277815
C -0.759529 -5.014757 0.955800
C -0.763003 -4.506898 2.393675
C 0.483335 -4.878051 3.206996
C 1.755834 -4.210688 2.676333
C 0.264161 -4.494466 4.673968
C 2.523267 -7.676843 -1.853894
C 2.532270 -6.311390 -1.165907
C 1.890160 -5.243726 -2.046787
C 1.966217 -3.842670 -1.460236
N 0.895448 -3.056555 -1.702798
O 2.950541 -3.445524 -0.832242
C 9.693833 -1.796811 2.830480
C 8.232797 -1.587150 2.423956
C 7.538951 -0.519552 3.269699
C 6.093207 -0.213253 2.859228
O 5.427169 -1.151242 2.332337
O 5.662965 0.949672 3.086304
C -2.898260 3.983904 -2.585061
C -1.683331 3.853325 -1.672022
O -1.774563 3.809825 -0.437522
C -4.166878 4.575345 -1.950432
C -4.799161 3.655516 -0.899438
C -3.920439 5.982026 -1.398003
N -0.499006 3.739819 -2.294916
C 0.709980 3.373099 -1.600724
C 1.941916 3.687159 -2.445884
O 1.903860 4.521459 -3.346707
N 3.029521 2.992187 -2.062689
C 4.387213 3.369099 -2.393029
C 5.141423 2.300612 -3.202582
O 5.930225 2.624511 -4.090024
C 5.180692 3.660800 -1.082079
C 6.526111 4.310624 -1.369218
O 4.422266 4.489139 -0.222449
N 4.935680 1.022052 -2.810535
C 5.748982 -0.085859 -3.269574
C 6.489888 -0.758388 -2.103029
C 7.379218 0.205351 -1.356121
C 8.593686 0.632297 -1.892720
C 6.982623 0.740649 -0.128314
C 9.393321 1.554829 -1.224670
C 7.766673 1.664754 0.553966
C 8.976293 2.070438 0.000566
O 9.727036 2.984692 0.698728
C -8.711156 -1.474730 -3.779914
C -8.394201 -0.084178 -3.211481
C -7.519945 -0.168269 -2.002556
C -6.166627 -0.028513 -1.847432
N -8.002861 -0.547225 -0.773053
C -6.963672 -0.628497 0.068845
N -5.829371 -0.309265 -0.543248
C -13.878994 -2.174862 -2.729049
C -13.687408 -2.618607 -1.277784
C -12.791698 -1.679909 -0.469301
C -11.289314 -1.725206 -0.819664
O -10.857343 -2.657758 -1.523636
O -10.600983 -0.779274 -0.312361
N 2.885481 -0.408744 2.079321
C 1.964159 -0.830298 0.988834
C 2.250042 -0.055920 -0.310668
O 3.267852 0.692662 -0.340447
C 0.504701 -0.669250 1.426466
C 0.106556 0.801069 1.635963
C -1.350220 0.906397 2.032708
N -2.205738 1.238943 1.067845
O -1.711816 0.682760 3.201072
O 1.436918 -0.216758 -1.246520
O 3.812243 2.429305 1.684077
O -1.260080 0.635861 -1.642727
H -12.911017 -2.136879 -3.238328
H -7.785134 -2.004177 -4.032524
H 1.499211 -7.987009 -2.092149
H 10.170691 -2.569886 2.216788
H -0.398664 7.057894 7.971606
H -3.105436 2.967378 -2.946725
H -9.315844 -1.386358 -4.690493
H -14.332590 -1.176102 -2.778420
H -5.465670 10.533338 5.242548
H -4.562962 9.951365 6.670113
H 1.365666 9.820219 3.708421
H 5.116836 -0.825327 -3.776412
H 0.689401 2.310403 -1.332156
H -4.002659 -4.946520 -2.025375
H -0.699241 -6.110293 0.930410
H -4.064722 -5.921009 3.682843
H 3.652225 -3.115125 6.318436
H 4.373768 -1.855477 7.362028
H 9.775304 -2.104213 3.881429
H 2.971958 -8.448575 -1.217792
H -14.528728 -2.867702 -3.277940
H -9.276324 -2.065679 -3.050572
H -2.996663 -3.636541 -2.627362
H 0.106021 -4.632238 0.404875
H 3.089572 -7.645491 -2.793051
H 10.269208 -0.870606 2.710348
H -5.658504 8.856928 5.783929
H 4.143497 -1.560657 5.620193
H 0.791918 3.954257 -0.675513
H -2.613480 4.576654 -3.464349
H 6.454430 0.318617 -3.999453
H 0.544393 10.634227 5.044009
H -4.186770 -5.495882 5.411854
H -5.545782 -5.188292 4.303313
H 4.338369 4.269133 -3.008781
H -2.745898 9.555560 2.067069
H -0.253216 6.238497 6.390349
H 1.197943 6.873435 7.187397
H -14.669928 -2.688819 -0.788274

H -13.244100 -3.620040 -1.265202
H -13.130645 -0.640142 -0.560799
H -12.863897 -1.923079 0.599472
H -7.886760 0.531759 -3.963480
H -9.329230 0.424143 -2.949838
H -9.039732 -0.725796 -0.563696
H -7.046608 -0.924452 1.103672
H -5.410155 0.230038 -2.575143
H -2.565054 -3.920233 0.775267
H -0.882344 -3.413760 2.390295
H -1.650951 -4.906753 2.899438
H 0.612724 -5.969980 3.148762
H 1.132530 -4.759111 5.289289
H 0.112754 -3.412054 4.760657
H -0.618995 -4.988464 5.096174
H 2.632512 -4.524749 3.254153
H 1.957626 -4.435936 1.624305
H 1.675495 -3.123286 2.783742
H -3.440079 -3.482594 5.981137
H -2.786705 -1.477018 3.941162
H -5.673062 -1.680836 4.169951
H -6.108459 -0.057399 4.736957
H -4.890397 -0.265624 3.459521
H -4.581324 -1.176768 6.371994
H -3.198361 1.135913 4.913787
H -4.533478 1.301909 6.074281
H -2.969730 0.679132 6.615136
H -1.013702 -0.224812 4.575317
H 0.036581 -0.666817 7.304459
H 0.446864 0.671966 6.209716
H 2.070311 -1.469877 7.725973
H 3.554615 -6.010417 -0.915034
H 1.981280 -6.372993 -0.218304
H 2.417511 -5.200572 -3.011064
H 0.847899 -5.506344 -2.252494
H 0.080754 -3.436204 -2.162261
H 0.954575 -2.055468 -1.513395
H 7.079868 -1.590138 -2.509365
H 5.758632 -1.198360 -1.414711
H 8.922135 0.245038 -2.854681
H 6.037179 0.425254 0.304425
H 7.433875 2.072548 1.503452
H 10.337312 1.876331 -1.661910
H 10.477612 3.252741 0.156361
H 7.674397 -2.525689 2.500686
H 8.187352 -1.289922 1.370067
H 7.516225 -0.837196 4.323394
H 8.101768 0.419954 3.245294
H 3.903802 -0.746921 2.002888
H 2.954990 0.615347 2.150552
H 2.551262 -0.754007 2.989063
H 2.179880 -1.883354 0.775127
H -0.134111 -1.118612 0.659967
H 0.339661 -1.226687 2.355863
H 0.695894 1.249223 2.443603
H 0.288945 1.373111 0.720613
H -3.196422 1.209641 1.263483
H -1.900660 1.324219 0.096937
H 2.911472 2.267429 -1.356547
H -0.435815 3.827218 -3.299070
H -4.887052 4.666065 -2.776227
H -4.140944 3.545531 -0.033753
H -5.756265 4.068110 -0.560409
H -3.522744 6.651580 -2.170385
H -4.850245 6.419442 -1.016976
H -3.199266 5.949575 -0.575838
H -4.995284 2.656801 -1.306387
H 5.358305 2.697249 -0.588903
H 7.142204 3.682808 -2.019551
H 6.375457 5.280481 -1.856245
H 7.056819 4.468909 -0.425699
H -3.763781 7.477655 1.313642
H -2.019748 7.516334 1.070231
H -3.146946 5.308369 1.840148
H -3.303266 6.043715 3.436998
H 0.233614 4.444833 2.274976
H -1.153245 4.350560 1.222901
H 1.115029 7.755902 4.795897
H -2.878118 9.396667 5.207984
H -1.159235 7.881528 3.885525
S -3.247780 -1.626165 -0.365010
H -5.031958 -2.790156 -1.502599
H -4.598054 -3.600490 -0.002060
H 4.551361 2.040993 2.211498
H 3.748579 1.843926 0.901182
H -0.342253 0.308229 -1.574284
H -1.821812 -0.161029 -1.577899
H 4.283752 0.834150 -2.051049
H 4.175156 3.957989 0.558281
H -4.570390 -0.782008 -0.304946

237
inactive_TS2
C 0.131201 7.050418 7.018374
C -0.103113 8.344275 6.270605
O -0.894683 9.206905 6.645985
N 0.639287 8.497504 5.138994
C 0.527976 9.684430 4.322681
C -0.763648 9.835112 3.516584
O -1.026342 10.909176 2.984697
N -1.543268 8.735140 3.451033
C -2.774822 8.700783 2.696707
C -4.028998 8.895409 3.569786
O -5.150285 8.752881 3.084432
C -2.886804 7.461725 1.799567
C -2.867177 6.079156 2.490236
C -1.472561 5.559668 2.806661
N -0.997706 4.583180 2.021138
O -0.804854 6.027032 3.746254
N -3.827719 9.286029 4.844012
C -4.942447 9.678310 5.683608
C -4.455096 -5.190579 4.396047
C -4.134713 -3.840320 3.791549
O -4.421796 -3.583011 2.628632
N -3.487766 -2.969533 4.610530
C -2.856776 -1.758352 4.123245
C -1.537031 -1.586082 4.865490
O -1.450749 -1.773227 6.079344
C -3.737548 -0.495475 4.335127
C -2.960045 0.804321 4.094601
C -4.962278 -0.554410 3.418859
N -0.506631 -1.122973 4.120874
C 0.676541 -0.574200 4.737867
C 1.758052 -1.609009 5.059151
O 1.955120 -2.580115 4.328872
N 2.493372 -1.336132 6.156402
C 3.720017 -2.037852 6.506438
C -3.362010 -4.112136 -1.711012
C -2.201693 -4.837381 -1.022900
O -1.687585 -5.833817 -1.543758
C -3.459647 -2.607615 -1.459887
N -1.819963 -4.382891 0.190540
C -0.759511 -5.014642 0.955872
C -0.902581 -4.681974 2.439730
C -0.144389 -5.633598 3.371670
C 1.351418 -5.699203 3.049755
C -0.366097 -5.219503 4.828847
C 2.523291 -7.676788 -1.853858
C 2.714747 -6.208018 -1.480235
C 1.919039 -5.258730 -2.376887
C 2.396652 -3.815381 -2.251486
N 1.500838 -2.852572 -2.559709
O 3.556281 -3.546350 -1.927812
C 9.693938 -1.796696 2.830309
C 8.200034 -1.800574 2.462803
C 7.348633 -0.992263 3.442919
C 5.847070 -0.874929 3.124569
O 5.363138 -1.600045 2.213926
O 5.199503 -0.042402 3.823788
C -2.898142 3.983757 -2.585277
C -1.743501 3.592513 -1.670803
O -1.899358 3.300137 -0.469479
C -4.094282 4.682785 -1.924217
C -4.884606 3.767018 -0.982894
C -3.674345 5.981808 -1.236031
N -0.539964 3.501944 -2.255672
C 0.640539 3.015445 -1.580555
C 1.886363 3.404243 -2.379872
O 1.803927 4.146263 -3.358922
N 3.016403 2.874205 -1.886248
C 4.346251 3.318346 -2.248155
C 5.184958 2.290407 -3.029137
O 6.145670 2.683963 -3.692960
C 5.072510 3.702667 -0.940305
C 6.537191 4.082771 -1.096885
O 4.319236 4.788526 -0.395042
N 4.861012 0.992936 -2.867235
C 5.749024 -0.085956 -3.269886
C 6.230665 -0.885989 -2.046655
C 6.988503 -0.039883 -1.051419
C 8.345349 0.234258 -1.225555
C 6.351562 0.517685 0.061308
C 9.049472 1.027435 -0.322764
C 7.036487 1.314698 0.971660
C 8.390206 1.567054 0.778745
O 9.035247 2.354353 1.702769
C -8.711097 -1.474757 -3.779893
C -8.197286 -0.111216 -3.292811
C -7.232052 -0.216099 -2.154523
C -5.899564 0.075089 -2.061647
N -7.623830 -0.693165 -0.920602
C -6.571069 -0.692761 -0.108495
N -5.511954 -0.224171 -0.772949
C -13.878983 -2.174865 -2.729047
C -13.585869 -2.274549 -1.227998
C -12.514098 -1.303536 -0.719733
C -11.046465 -1.706929 -0.975172
O -10.792166 -2.783588 -1.538566
O -10.190060 -0.853136 -0.550730
N 2.700436 -1.321043 1.810488
C 2.149482 -1.384605 0.428914
C 2.537826 -0.121050 -0.362412
O 2.872403 0.914716 0.281496
C 0.617276 -1.609308 0.433174
C -0.267526 -0.368917 0.270059
C -1.757692 -0.714028 0.353080
N -2.712412 0.349845 0.133862
O -2.116288 -1.582571 1.176196
O 2.448679 -0.206525 -1.607389
O 2.885443 1.212939 3.094781
O -3.121826 0.804873 -3.406141
H -12.971275 -2.385377 -3.303707
H -7.878388 -2.096838 -4.127118
H 1.466515 -7.964174 -1.801494
H 10.277858 -2.386005 2.114071
H -0.428257 7.073150 7.953758
H -3.222780 3.036071 -3.037554
H -9.404183 -1.333045 -4.616483
H -14.228811 -1.168967 -2.995917
H -5.462637 10.544382 5.260574
H -4.556429 9.931587 6.672626
H 1.366022 9.708462 3.620848
H 5.220933 -0.758294 -3.955101
H 0.609213 1.927287 -1.460169
H -4.289212 -4.594906 -1.372501
H -0.830608 -6.095864 0.794363
H -3.725293 -5.914187 4.016420
H 3.623229 -3.078082 6.191770
H 3.857155 -1.996336 7.589785
H 9.860969 -2.216471 3.830827
H 3.087206 -8.332449 -1.180440
H -14.650861 -2.892748 -3.031293
H -9.244379 -2.010948 -2.988286
H -3.253262 -4.318437 -2.778991
H 0.224746 -4.703940 0.574938
H 2.870268 -7.867252 -2.877289
H 10.090117 -0.773415 2.828479
H -5.663186 8.860066 5.772715
H 4.578089 -1.587506 5.994649
H 0.710968 3.453773 -0.579733
H -2.507979 4.611364 -3.397105
H 6.587618 0.369295 -3.801447
H 0.598130 10.570534 4.958036
H -4.415552 -5.199470 5.489945
H -5.445218 -5.505412 4.060590
H 4.252587 4.203501 -2.881972
H -2.764216 9.580607 2.042906
H -0.212829 6.208192 6.406937
H 1.195891 6.906299 7.227118
H -14.517841 -2.091453 -0.673928
H -13.266991 -3.295935 -0.994029
H -12.662803 -0.301420 -1.143958
H -12.610358 -1.176362 0.366107
H -7.698715 0.425636 -4.107697
H -9.048976 0.499800 -2.971495
H -8.671649 -0.917437 -0.702351
H -6.563115 -1.029154 0.916139
H -5.180435 0.442394 -2.783387
H -2.152853 -3.483407 0.529968
H -0.575485 -3.651063 2.632022
H -1.969894 -4.715625 2.685657
H -0.568718 -6.639961 3.228647

H 0.106812 -5.927412 5.520417
H 0.064708 -4.228997 5.012980
H -1.435785 -5.172473 5.069503
H 1.866661 -6.379812 3.738444
H 1.533699 -6.059541 2.031070
H 1.809632 -4.710548 3.158844
H -3.233747 -3.249472 5.548241
H -2.686496 -1.878649 3.050124
H -5.568108 -1.443564 3.610293
H -5.590616 0.333068 3.560069
H -4.636902 -0.599764 2.372648
H -4.069417 -0.506767 5.382184
H -2.475907 0.800577 3.110146
H -3.640197 1.662261 4.129604
H -2.179466 0.968653 4.844116
H -0.638647 -1.064473 3.118758
H 0.374009 -0.047732 5.649243
H 1.136300 0.159212 4.062930
H 2.313592 -0.459249 6.620983
H 3.773253 -5.937739 -1.539768
H 2.414414 -6.048963 -0.436248
H 2.060080 -5.542115 -3.430318
H 0.842733 -5.337372 -2.176111
H 0.510066 -3.045948 -2.645663
H 1.784486 -1.883108 -2.399733
H 6.866193 -1.704561 -2.406820
H 5.365564 -1.359498 -1.568801
H 8.868844 -0.181322 -2.083816
H 5.299067 0.318667 0.234748
H 6.531943 1.718972 1.844258
H 10.109149 1.223624 -0.478789
H 9.959498 2.445142 1.444235
H 7.826045 -2.829557 2.420139
H 8.070739 -1.390860 1.455050
H 7.426629 -1.418596 4.454232
H 7.734282 0.031690 3.524858
H 3.766724 -1.429071 1.873871
H 2.515862 -0.406959 2.259229
H 2.312120 -2.043023 2.431970
H 2.636652 -2.234863 -0.054632
H 0.365929 -2.290414 -0.382023
H 0.342933 -2.138502 1.353214
H -0.074930 0.367076 1.070392
H -0.048297 0.130148 -0.676597
H -2.942993 0.749775 1.043055
H -2.345864 1.105627 -0.443144
H 2.936337 2.255603 -1.070519
H -0.385891 3.836457 -3.199335
H -4.765900 4.948911 -2.752713
H -4.285599 3.489767 -0.111743
H -5.795340 4.270312 -0.637413
H -3.184409 6.667989 -1.937150
H -4.540070 6.497963 -0.805733
H -2.968164 5.772253 -0.426361
H -5.181437 2.843393 -1.492311
H 4.997903 2.838966 -0.265381
H 7.135073 3.233601 -1.435022
H 6.648819 4.898987 -1.816615
H 6.929790 4.403678 -0.123909
H -3.838855 7.552937 1.272370
H -2.093397 7.510238 1.045509
H -3.383413 5.363436 1.842558
H -3.426207 6.121926 3.431803
H -0.062305 4.245121 2.204198
H -1.478976 4.198498 1.207618
H 1.100258 7.681688 4.760686
H -2.881620 9.388137 5.190142
H -1.199151 7.848639 3.823322
S -1.950901 -1.670201 -1.877049
H -4.536848 -0.131214 -0.421642
H -4.303184 -2.223938 -2.041702
H -3.687489 -2.419377 -0.400409
H 3.751611 0.826265 3.391870
H 3.005518 1.417962 2.148179
H -2.744889 0.676024 -4.284657
H -2.755985 0.038986 -2.882236
H 3.988338 0.734156 -2.402374
H 4.611876 4.920618 0.513815

237
inactive_TS3
C 0.131361 7.050407 7.017995
C -0.107159 8.350623 6.282767
O -0.908986 9.202651 6.660456
N 0.642118 8.521554 5.158020
C 0.526074 9.714328 4.350981
C -0.757493 9.858542 3.530596
O -1.018713 10.931694 2.996111
N -1.531477 8.755359 3.457019
C -2.760631 8.717076 2.698865
C -4.018570 8.907153 3.568357
O -5.137180 8.766100 3.076880
C -2.865232 7.477920 1.800068
C -2.843890 6.094902 2.489466
C -1.450619 5.582916 2.823580
N -0.959933 4.611869 2.040679
O -0.797265 6.049557 3.773313
N -3.823486 9.291900 4.845524
C -4.942209 9.678501 5.683124
C -4.455450 -5.190459 4.396215
C -4.061365 -3.902342 3.701931
O -4.274425 -3.738966 2.506593
N -3.431403 -2.986868 4.482726
C -2.779830 -1.802494 3.957490
C -1.493417 -1.598716 4.749378
O -1.433550 -1.819783 5.958880
C -3.676864 -0.536663 4.069780
C -2.914807 0.751694 3.737288
C -4.883168 -0.680715 3.139724
N -0.452549 -1.085316 4.051627
C 0.701724 -0.537698 4.722366
C 1.785945 -1.573093 5.035234
O 2.005738 -2.522016 4.282924
N 2.498031 -1.328590 6.154255
C 3.719812 -2.037870 6.506320
C -3.361655 -4.112189 -1.710989
C -2.180323 -4.803381 -1.021481
O -1.608440 -5.750357 -1.574441
C -3.514335 -2.624156 -1.454396
N -1.836220 -4.378817 0.211907
C -0.760009 -5.014857 0.955634
C -0.840557 -4.656545 2.435890
C -0.053010 -5.601681 3.350013
C 1.435532 -5.656040 2.994707
C -0.248343 -5.190696 4.811673
C 2.522899 -7.677299 -1.853658
C 2.759793 -6.207938 -1.510036
C 1.955343 -5.260027 -2.399127
C 2.424381 -3.814789 -2.273540
N 1.509013 -2.859899 -2.558299
O 3.584755 -3.533105 -1.970576
C 9.693728 -1.797230 2.830311
C 8.207650 -1.773390 2.419052
C 7.333904 -0.976963 3.391238
C 5.834568 -0.840775 3.054579
O 5.337853 -1.566634 2.151980
O 5.201051 0.012892 3.742198
C -2.897646 3.983165 -2.585310
C -1.736319 3.645075 -1.658729
O -1.886532 3.368126 -0.455402
C -4.106597 4.676571 -1.938773
C -4.884382 3.769003 -0.979315
C -3.707738 5.996081 -1.275952
N -0.524045 3.582087 -2.236731
C 0.669301 3.183522 -1.525878
C 1.895486 3.472677 -2.394466
O 1.801482 4.190453 -3.390941
N 3.022764 2.911662 -1.931181
C 4.353196 3.339524 -2.313738
C 5.185564 2.293464 -3.075676
O 6.147528 2.672372 -3.745800
C 5.087664 3.751140 -1.018327
C 6.551162 4.128524 -1.189886
O 4.334674 4.846782 -0.492679
N 4.858916 0.999759 -2.890445
C 5.749012 -0.086526 -3.269895
C 6.232421 -0.858659 -2.030120
C 6.988510 0.009727 -1.053152
C 8.342511 0.290182 -1.238596
C 6.353305 0.578976 0.054630
C 9.046119 1.100115 -0.350496
C 7.038350 1.390749 0.951935
C 8.389506 1.649027 0.748059
O 9.034339 2.452406 1.657998
C -8.711215 -1.474799 -3.779845
C -8.012785 -0.137824 -3.469797
C -7.029534 -0.185113 -2.337295
C -5.691912 0.109860 -2.249275
N -7.420485 -0.530097 -1.064214
C -6.352934 -0.441202 -0.258823
N -5.279703 -0.051549 -0.940351
C -13.879219 -2.174771 -2.729117
C -13.569027 -1.966641 -1.241459
C -12.440707 -0.968770 -0.946078
C -10.993029 -1.503759 -1.063308
O -10.808516 -2.674478 -1.443600
O -10.091525 -0.656254 -0.748424
N 2.641153 -1.265270 1.756704
C 2.074291 -1.324504 0.380436
C 2.483812 -0.074119 -0.419691
O 2.830615 0.960746 0.216860
C 0.533386 -1.537118 0.383813
C -0.366396 -0.334933 0.046526
C -1.853172 -0.766565 -0.022106
N -2.785433 0.411992 -0.275848
O -2.269211 -1.476760 0.957331
O 2.399705 -0.172668 -1.665146
O 2.883054 1.251158 3.040070
O -2.348679 0.987967 -3.360303
H -12.990398 -2.548351 -3.247485
H -7.980366 -2.224067 -4.105794
H 1.461914 -7.938062 -1.762930
H 10.289762 -2.376469 2.116020
H -0.419928 7.066543 7.958367
H -3.202466 3.024020 -3.021965
H -9.436584 -1.331447 -4.588195
H -14.183102 -1.232182 -3.203471
H -4.560549 9.923001 6.676000
H -5.664073 8.860235 5.761703
H 1.372146 9.755373 3.659676
H 5.221766 -0.774198 -3.940289
H 0.633184 2.124658 -1.250706
H -4.275570 -4.623237 -1.379946
H -0.851727 -6.098120 0.817850
H -3.722158 -5.959316 4.128336
H 4.582163 -1.596645 5.993932
H 3.617192 -3.079115 6.196620
H 9.825549 -2.244069 3.824172
H 3.092603 -8.335638 -1.187938
H -14.690025 -2.900321 -2.867760
H -9.245919 -1.869721 -2.911610
H -3.244211 -4.318327 -2.778472
H 0.214032 -4.728938 0.531307
H 2.831963 -7.892193 -2.884334
H 10.102078 -0.779440 2.865681
H -5.459340 10.548662 5.264780
H 3.857069 -1.991886 7.589264
H 0.771619 3.756182 -0.596349
H -2.519906 4.599694 -3.411516
H 6.586319 0.358853 -3.811562
H 0.578176 10.595078 4.995489
H -4.487919 -5.106046 5.487088
H -5.430043 -5.510198 4.022497
H 4.258646 4.210759 -2.966322
H -2.751158 9.597181 2.045577
H -0.221441 6.215302 6.401752
H 1.197363 6.900800 7.215678
H -14.484505 -1.622003 -0.738313
H -13.301348 -2.931425 -0.797163
H -12.521003 -0.088764 -1.599344
H -12.543901 -0.581535 0.075334
H -7.484526 0.230936 -4.357070
H -8.779654 0.605694 -3.219787
H -8.435950 -0.735386 -0.824422
H -6.371067 -0.671598 0.794802
H -4.997542 0.422991 -3.017097
H -2.214828 -3.507526 0.585062
H -0.497615 -3.625558 2.599816
H -1.895122 -4.682630 2.726285
H -0.473417 -6.610851 3.215625
H 0.243848 -5.896207 5.492163
H 0.177683 -4.197188 4.989023
H -1.313337 -5.150868 5.073222
H 1.970868 -6.334836 3.669575
H 1.597930 -6.012521 1.971182
H 1.889685 -4.664635 3.096369
H -3.232840 -3.197959 5.451459
H -2.563281 -1.965875 2.897110
H -5.501430 -1.542225 3.404408
H -5.508934 0.219199 3.183188
H -4.526715 -0.830378 2.114489
H -4.022425 -0.476701 5.111359

H -2.445682 0.671538 2.749392
H -3.606179 1.601151 3.715302
H -2.131276 0.979801 4.466928
H -0.584751 -0.932788 3.060359
H 0.368768 -0.047744 5.643664
H 1.170016 0.221642 4.083000
H 2.298834 -0.471871 6.647377
H 3.821623 -5.961408 -1.605102
H 2.494300 -6.025005 -0.460648
H 2.090509 -5.541068 -3.453936
H 0.881126 -5.345949 -2.189802
H 0.524961 -3.079728 -2.634317
H 1.778701 -1.884655 -2.406070
H 6.869276 -1.683653 -2.372717
H 5.369264 -1.324030 -1.541081
H 8.864202 -0.134024 -2.093709
H 5.302929 0.374466 0.235253
H 6.536576 1.800771 1.823366
H 10.103473 1.301511 -0.515214
H 9.957572 2.542672 1.395610
H 7.824033 -2.796791 2.341346
H 8.114570 -1.339201 1.417241
H 7.389598 -1.420706 4.396709
H 7.722917 0.043273 3.498276
H 3.704313 -1.385138 1.805800
H 2.477340 -0.346542 2.207088
H 2.255846 -1.980987 2.388673
H 2.549888 -2.182762 -0.099331
H 0.308454 -2.323346 -0.340183
H 0.233694 -1.949503 1.353459
H -0.282659 0.451204 0.813092
H -0.053236 0.108945 -0.905523
H -2.780536 1.002053 0.558796
H -2.452497 1.005602 -1.040449
H 2.945622 2.303811 -1.106737
H -0.360991 3.954302 -3.165624
H -4.780515 4.915100 -2.773520
H -4.287110 3.527441 -0.096258
H -5.805999 4.263731 -0.651623
H -3.223289 6.674342 -1.988631
H -4.583123 6.508869 -0.861671
H -3.004677 5.814079 -0.456648
H -5.164030 2.826407 -1.462962
H 5.018229 2.901582 -0.324908
H 7.147012 3.272314 -1.513421
H 6.659639 4.929720 -1.926718
H 6.948935 4.468578 -0.225432
H -3.815656 7.565733 1.268911
H -2.068884 7.529270 1.049073
H -3.346903 5.376624 1.834096
H -3.415525 6.132447 3.423615
H -0.028428 4.272060 2.239326
H -1.432278 4.227328 1.222291
H 1.113507 7.713362 4.776433
H -2.879529 9.392127 5.197849
H -1.190083 7.871699 3.837858
S -1.987139 -1.706613 -1.808456
H -4.045315 0.116484 -0.554797
H -4.334469 -2.229676 -2.057297
H -3.745934 -2.426633 -0.400081
H 3.753173 0.861502 3.327465
H 2.999682 1.474458 2.098064
H -1.463456 1.159749 -3.704233
H -2.325551 0.049815 -3.060817
H 3.981652 0.751184 -2.429504
H 4.662238 5.027928 0.395666

237
inactive_TS4
C 0.131258 7.050330 7.018344
C -0.108300 8.381728 6.341527
O -0.924278 9.207766 6.745528
N 0.655801 8.610889 5.236512
C 0.531238 9.831470 4.473452
C -0.726030 9.975418 3.612101
O -0.980052 11.055158 3.087088
N -1.484775 8.865997 3.496058
C -2.697078 8.824920 2.710498
C -3.974208 8.982342 3.559546
O -5.081347 8.850352 3.041424
C -2.765131 7.598941 1.787845
C -2.733966 6.204886 2.453392
C -1.344425 5.722276 2.843275
N -0.789073 4.792956 2.055551
O -0.750866 6.173318 3.839862
N -3.806749 9.327771 4.852371
C -4.942284 9.678284 5.683483
C -4.455405 -5.190576 4.396329
C -3.959751 -3.964746 3.656579
O -4.040123 -3.895297 2.431555
N -3.403141 -3.000006 4.423585
C -2.750423 -1.823576 3.882828
C -1.494388 -1.575976 4.713485
O -1.474504 -1.772612 5.928030
C -3.676151 -0.574648 3.906121
C -2.943852 0.697026 3.461061
C -4.896533 -0.806848 3.011942
N -0.434440 -1.067032 4.040275
C 0.704551 -0.512179 4.733003
C 1.796710 -1.540095 5.040709
O 2.032825 -2.473155 4.273126
N 2.500715 -1.315930 6.167769
C 3.719771 -2.037952 6.506728
C -3.361976 -4.112093 -1.711054
C -2.116136 -4.731819 -1.061109
O -1.288617 -5.350938 -1.738418
C -3.482357 -2.612311 -1.425570
N -1.956372 -4.548834 0.267194
C -0.759637 -5.014946 0.956076
C -0.815934 -4.739989 2.451994
C 0.168161 -5.589737 3.265134
C 1.615965 -5.431935 2.793300
C 0.032737 -5.252570 4.752118
C 2.523065 -7.677272 -1.853411
C 2.879593 -6.225227 -1.545880
C 2.120113 -5.245090 -2.436080
C 2.603539 -3.811913 -2.265488
N 1.740603 -2.844838 -2.660051
O 3.723539 -3.545341 -1.828063
C 9.693767 -1.797207 2.830751
C 8.208295 -1.726418 2.447504
C 7.386098 -0.885718 3.424206
C 5.889904 -0.747124 3.104207
O 5.401075 -1.493768 2.210938
O 5.249037 0.110916 3.774606
C -2.897545 3.983430 -2.584610
C -1.698095 3.788452 -1.662051
O -1.795544 3.719356 -0.426181
C -4.130535 4.656551 -1.961584
C -4.828589 3.791971 -0.905657
C -3.803120 6.053501 -1.425871
N -0.508350 3.605433 -2.268450
C 0.699630 3.294027 -1.538342
C 1.918288 3.493481 -2.441354
O 1.828210 4.151978 -3.476745
N 3.043549 2.938495 -1.959540
C 4.375757 3.356870 -2.352268
C 5.206361 2.302197 -3.104321
O 6.167681 2.671172 -3.780743
C 5.113760 3.780504 -1.062951
C 6.574518 4.163409 -1.244903
O 4.357403 4.875691 -0.541315
N 4.874726 1.013787 -2.900768
C 5.749072 -0.086408 -3.269493
C 6.242311 -0.842420 -2.023252
C 7.016397 0.033608 -1.067386
C 8.368927 0.305673 -1.275186
C 6.400747 0.617621 0.043628
C 9.089170 1.121208 -0.405833
C 7.102101 1.435898 0.922143
C 8.451465 1.685225 0.696304
O 9.112830 2.494420 1.588589
C -8.711086 -1.474856 -3.779801
C -8.081486 -0.104481 -3.494373
C -7.208575 -0.096818 -2.279618
C -5.866321 0.134543 -2.094945
N -7.707084 -0.356491 -1.029273
C -6.677188 -0.269787 -0.160757
N -5.536589 0.026469 -0.760010
C -13.879028 -2.174809 -2.729096
C -13.704498 -2.159978 -1.208806
C -12.693025 -1.123693 -0.714755
C -11.202970 -1.451283 -0.974779
O -10.903301 -2.574921 -1.425985
O -10.399497 -0.512039 -0.675737
N 2.785340 -1.224938 1.793405
C 2.309211 -1.348018 0.394265
C 2.632569 -0.074362 -0.407981
O 2.914045 0.982010 0.221159
C 0.797016 -1.672426 0.325689
C -0.145465 -0.490815 0.025797
C -1.566149 -1.024293 -0.062153
N -2.592861 0.784384 -0.409844
O -2.118081 -1.496178 0.922476
O 2.536872 -0.184426 -1.653124
O 2.902211 1.318158 3.056591
O -1.916538 1.015278 -3.482305
H -12.922192 -2.402638 -3.208668
H -7.930531 -2.225291 -3.952993
H 1.448350 -7.853762 -1.727670
H 10.257618 -2.406722 2.115405
H -0.407387 7.030471 7.966035
H -3.152589 2.979735 -2.948512
H -9.340491 -1.424770 -4.676594
H -14.223932 -1.198333 -3.094778
H -5.456456 10.561071 5.288512
H -4.579370 9.887415 6.691362
H 1.399492 9.926990 3.815817
H 5.207320 -0.781617 -3.920560
H 0.669580 2.273355 -1.138767
H -4.265441 -4.610689 -1.338831
H -0.654888 -6.093890 0.777732
H -3.759296 -6.013130 4.199779
H 3.603920 -3.078942 6.200935
H 3.869842 -1.989777 7.587628
H 9.828852 -2.235065 3.828320
H 3.060104 -8.366435 -1.191739
H -14.611307 -2.929888 -3.041738
H -9.334293 -1.815945 -2.945982
H -3.278645 -4.308750 -2.782115
H 0.122051 -4.547081 0.500227
H 2.782640 -7.934516 -2.888009
H 10.140342 -0.794911 2.844957
H -5.661085 8.854358 5.718675
H 4.580875 -1.605754 5.984887
H 0.812194 3.970928 -0.682866
H -2.568484 4.551000 -3.465297
H 6.584432 0.338785 -3.830473
H 0.534461 10.684763 5.155812
H -4.532867 -5.046657 5.478149
H -5.430056 -5.473945 3.993533
H 4.281167 4.221046 -3.014270
H -2.686373 9.716623 2.073400
H -0.237893 6.247275 6.370097
H 1.198502 6.883222 7.193975
H -14.679134 -1.964410 -0.736720
H -13.376184 -3.151491 -0.878564
H -12.901875 -0.137945 -1.151198
H -12.794023 -0.992318 0.370920
H -7.478738 0.225357 -4.349715
H -8.882624 0.634069 -3.363311
H -8.725801 -0.532680 -0.827110
H -6.799556 -0.433798 0.900280
H -5.120228 0.382616 -2.840943
H -2.643000 -4.028452 0.805786
H -0.636023 -3.674127 2.651015
H -1.827371 -4.959078 2.805582
H -0.121317 -6.642862 3.123074
H 0.667931 -5.902793 5.365519
H 0.333893 -4.216078 4.937264
H -1.004400 -5.370290 5.091522
H 2.291930 -6.039321 3.406405
H 1.742146 -5.747980 1.751761
H 1.939274 -4.390860 2.887091
H -3.311773 -3.132539 5.421650
H -2.492874 -2.032378 2.843696
H -5.472276 -1.682304 3.324204
H -5.558509 0.065905 3.051960
H -4.586372 -0.959001 1.972475
H -4.010162 -0.442355 4.945374
H -2.508488 0.551177 2.465723
H -3.652742 1.529257 3.396585
H -2.147522 0.991752 4.152163
H -0.534080 -0.930333 3.043468
H 0.353237 -0.038808 5.655551
H 1.171216 0.260186 4.107628
H 2.292388 -0.476363 6.685970
H 3.954703 -6.060549 -1.666806
H 2.653986 -6.002130 -0.495477
H 2.269928 -5.509740 -3.493213
H 1.040231 -5.308538 -2.249136
H 0.768996 -3.067540 -2.827804

H 1.996005 -1.874151 -2.459907
H 6.868625 -1.677488 -2.360736
H 5.382080 -1.295074 -1.517677
H 8.876143 -0.129537 -2.133406
H 5.352094 0.422076 0.241500
H 6.614135 1.860433 1.794477
H 10.144891 1.315598 -0.588136
H 10.031245 2.583165 1.309227
H 7.784739 -2.735171 2.393950
H 8.108683 -1.303744 1.441494
H 7.457612 -1.303717 4.439479
H 7.794455 0.130226 3.493032
H 3.858260 -1.317262 1.899049
H 2.567550 -0.300524 2.204054
H 2.388924 -1.934344 2.424320
H 2.873452 -2.169635 -0.055408
H 0.651822 -2.422628 -0.454434
H 0.489901 -2.151581 1.263849
H -0.113499 0.264109 0.820143
H 0.142272 -0.006615 -0.911937
H -2.447085 1.566307 0.226529
H -2.428040 1.141253 -1.351228
H 2.968085 2.361537 -1.114726
H -0.379767 3.840826 -3.245560
H -4.836842 4.779651 -2.794512
H -4.204870 3.680567 -0.014876
H -5.776522 4.254379 -0.607647
H -3.368007 6.691505 -2.204950
H -4.705773 6.547764 -1.049075
H -3.084776 5.991291 -0.601667
H -5.053095 2.788831 -1.284091
H 5.052181 2.935241 -0.363127
H 7.171294 3.308711 -1.570485
H 6.675014 4.962796 -1.984873
H 6.977933 4.507684 -0.284183
H -3.707088 7.679195 1.239623
H -1.955509 7.674440 1.052926
H -3.183109 5.486921 1.759493
H -3.349876 6.208258 3.359715
H 0.139139 4.468911 2.291122
H -1.220905 4.427155 1.205865
H 1.132741 7.821173 4.824174
H -2.870622 9.422859 5.225283
H -1.151659 7.982859 3.885089
S -1.916676 -1.738069 -1.746613
H -3.584419 0.495261 -0.360900
H -4.271957 -2.157492 -2.026569
H -3.719850 -2.411937 -0.375353
H 3.775249 0.950360 3.356957
H 3.031296 1.544074 2.117432
H -0.966204 1.173216 -3.541100
H -2.004031 0.082580 -3.211627
H 3.999708 0.775535 -2.430161
H 4.715983 5.092803 0.326820

237
inactive_fGln123_ES
H 5.491513 1.422296 0.573613
H 4.273880 1.675136 -0.308608
H -4.043473 -1.373478 -4.526260
H -5.514603 -1.041079 -4.217449
N -2.351852 -4.070881 0.277802
N 2.701226 -0.115754 1.501866
C 2.221112 -1.062284 0.464103
C -3.808646 -3.833117 -1.688972
C -2.924395 -4.681522 -0.778419
C -3.664220 -2.319378 -1.722249
C -1.434670 -4.731184 1.185231
C -1.890161 -4.662413 2.641253
C -0.980443 -5.464285 3.585793
C 0.493706 -5.045398 3.508687
C -1.503136 -5.386861 5.023262
C 2.678687 -0.517546 -0.899168
C 0.698326 -1.248338 0.506970
C -0.104946 0.032952 0.263744
C -1.601636 -0.216688 0.293868
O -2.801626 -5.886120 -1.012209
O -2.070654 -1.198666 0.897169
O 2.765162 -1.321933 -1.852352
O 2.889324 0.726909 -0.959489
S -2.211133 -1.862222 -2.748580
H -4.852050 -4.087674 -1.456625
H -1.297446 -5.777730 0.899005
H -0.453918 -4.254877 1.079372
H -3.656663 -4.242451 -2.694788
H -2.380403 -3.058938 0.354981
H -1.937284 -3.611097 2.964045
H -2.913588 -5.053461 2.714261
H -1.037735 -6.516139 3.267114
H -0.895146 -6.001013 5.698374
H -1.481977 -4.355139 5.397134
H -2.540978 -5.732085 5.089334
H 1.093635 -5.629160 4.217213
H 0.923418 -5.187643 2.512187
H 0.628779 -3.986620 3.764856
H 0.437305 -2.001894 -0.243170
H 0.427144 -1.674062 1.478563
H 0.098443 0.764286 1.059350
H 0.169802 0.513830 -0.682155
H -4.560366 -1.886207 -2.167790
H -3.567178 -1.876884 -0.729964
H 2.695489 -2.028419 0.654273
H 3.777585 -0.177680 1.579546
H 2.486761 0.838030 1.203184
H 2.290302 -0.280307 2.433999
N 1.645666 -3.930573 -2.078648
C 0.684467 -8.610837 -0.998779
C 1.227509 -7.264630 -0.517529
C 0.795321 -6.115618 -1.428370
C 1.443189 -4.788973 -1.056726
O 1.760245 -4.517282 0.103076
H -0.408540 -8.582331 -1.078061
H 0.951575 -9.417038 -0.305861
H 1.084303 -8.867921 -1.987263
H 2.322462 -7.298255 -0.465557
H 0.882224 -7.057717 0.501156
H 1.028171 -6.357901 -2.473943
H -0.295944 -5.991744 -1.373626
H 1.290143 -4.135704 -2.998612
H 2.034369 -2.997622 -1.908388
C 8.882493 -3.298668 1.873146
C 7.392392 -2.963928 1.867494
C 7.107965 -1.587786 2.482750
C 5.635149 -1.211166 2.346318
O 4.787612 -1.861663 2.986861
O 5.345663 -0.251327 1.545333
H 9.073394 -4.285161 1.435831
H 9.287150 -3.296225 2.893501
H 9.443427 -2.559800 1.288009
H 7.026639 -2.980464 0.834651
H 6.823041 -3.721368 2.417499
H 7.361735 -1.612105 3.550296
H 7.731648 -0.827258 2.003067
N 0.060455 2.538997 -4.088278
N 3.421844 1.263075 -3.705249
N 4.655137 -1.229519 -4.160872
C -2.065454 3.680480 -4.359997
C -1.106514 2.848504 -3.504218
C -3.243575 4.342406 -3.624417
C -4.275224 3.333954 -3.115121
C -2.776064 5.287340 -2.511980
C 1.089577 1.768175 -3.431593
C 2.342528 1.765858 -4.318498
C 4.754626 1.212846 -4.270606
C 5.123528 -0.163369 -4.850266
C 5.766958 1.564508 -3.150752
C 7.219819 1.486255 -3.603279
C 5.168947 -2.571543 -4.356404
C 5.656248 -3.171366 -3.028044
C 6.790852 -2.377140 -2.428347
C 8.084447 -2.489921 -2.939728
C 6.576075 -1.466188 -1.391042
C 9.132585 -1.726651 -2.435438
C 7.609143 -0.687246 -0.882963
C 8.890298 -0.820152 -1.405680
O -1.374221 2.464776 -2.355173
O 2.290794 2.183260 -5.476773
O 5.878764 -0.254203 -5.816909
O 5.466132 2.857663 -2.664574
O 9.884198 -0.038183 -0.872175
H -2.451281 3.019895 -5.148016
H 0.767336 0.730950 -3.269240
H 4.394141 -3.217813 -4.789802
H 5.987106 -2.494173 -5.075724
H -1.486528 4.465058 -4.863694
H 1.317475 2.188479 -2.447390
H 5.972594 -4.205519 -3.216412
H 4.815674 -3.217704 -2.325652
H 3.986563 -1.088265 -3.402956
H -3.861233 2.715257 -2.315711
H -5.159767 3.845318 -2.719832
H -2.057599 6.025002 -2.889915
H -3.628307 5.832542 -2.090472
H -3.740853 4.958486 -4.387425
H 0.294031 2.842165 -5.028112
H 3.319946 1.067299 -2.704385
H 4.825524 1.940555 -5.082291
H 5.616582 0.833475 -2.343996
H 7.505853 0.468380 -3.882826
H 7.388865 2.145691 -4.461020
H 7.857490 1.811369 -2.775269
H 8.280145 -3.185424 -3.753091
H 10.134044 -1.833213 -2.849030
H 7.414599 0.021573 -0.086348
H 5.583903 -1.352139 -0.961251
H 10.692651 -0.164345 -1.381961
H 5.478219 2.802288 -1.689691
H -2.294820 4.725327 -1.705718
H -4.609223 2.669899 -3.920765
N -7.637980 0.197111 -0.899674
N -5.427668 0.279632 -0.655419
C -8.900826 -0.548327 -3.794615
C -7.987934 0.617080 -3.368684
C -7.127475 0.389909 -2.157559
C -5.760521 0.431522 -1.986881
C -6.596814 0.144949 -0.045274
H -8.304846 -1.437664 -4.033760
H -9.453807 -0.263992 -4.697260
H -9.622141 -0.821284 -3.015190
H -7.318949 0.885697 -4.196526
H -8.613525 1.498790 -3.178058
H -5.002064 0.608159 -2.738839
H -6.731573 0.023899 1.020882
C -13.978350 -0.138956 -2.012453
C -13.677732 -0.812787 -0.672419
C -12.500467 -0.176698 0.066563
C -11.113959 -0.436319 -0.560302
O -10.981202 -1.393203 -1.348510
O -10.197727 0.360762 -0.177557
H -13.106611 -0.207135 -2.670685
H -14.217411 0.921888 -1.867662
H -14.828663 -0.611761 -2.519355
H -14.573152 -0.766902 -0.035167
H -13.450859 -1.870320 -0.843410
H -12.636930 0.907492 0.163253
H -12.450602 -0.567057 1.092467
O -4.880219 -1.112899 -4.943527
N -4.088848 -0.309466 2.682041
N -1.499339 -0.705297 3.797515
N 1.627099 -1.699095 5.505179
C -5.242163 -2.438557 2.501299
C -4.601045 -1.364705 3.358738
C -3.384435 0.794484 3.311393
C -2.286008 0.294089 4.282442
C -4.320271 1.823428 3.984602
C -3.612353 3.175442 4.105392
C -5.624491 1.981681 3.199383
C -0.627755 -1.427443 4.677764
C 0.849246 -1.003468 4.652896
C 3.067546 -1.501393 5.698976
O -4.541946 -1.453334 4.582751
O -2.112400 0.784329 5.389647
O 1.275348 -0.113541 3.915676
H 3.356452 -2.126895 6.545341
H 3.635544 -1.776502 4.804420
H -6.059836 -2.854279 3.092070
H 3.265327 -0.452694 5.938540
H -5.614010 -2.062548 1.544055
H -4.509834 -3.228408 2.301851
H -6.237200 1.077494 3.245825
H -6.215433 2.806445 3.612627
H -5.433776 2.202194 2.140778
H -3.433631 3.591542 3.104783
H -4.237913 3.890253 4.651685
H -2.655586 3.088729 4.626412
H -4.547529 1.453491 4.989075
H -2.864339 1.300761 2.485400
H -1.700732 -1.081301 2.874544
H -0.678715 -2.491433 4.427122
H -1.002842 -1.308095 5.700173
H 1.178520 -2.427056 6.040581
H -4.088767 -0.355508 1.672988

O 5.080529 2.114667 0.023172
C 0.131958 7.049034 7.019989
C -0.139626 8.391122 6.378790
O -1.027226 9.151878 6.760648
N 0.663812 8.709107 5.325533
C 0.527913 9.963577 4.620847
C -0.702639 10.119821 3.723453
O -0.965494 11.218636 3.245616
N -1.430297 9.001483 3.522062
C -2.629586 8.987233 2.715824
C -3.923833 9.158007 3.535464
O -5.015494 9.114954 2.970876
C -2.716381 7.748361 1.813409
C -2.789132 6.378902 2.527658
C -1.432950 5.800578 2.895906
N -1.011512 4.746539 2.171837
O -0.730482 6.283071 3.793061
N -3.787970 9.379067 4.856458
C -4.941125 9.676844 5.684910
H -0.408943 6.993708 7.965084
H -5.480085 10.546848 5.297202
H -4.578845 9.886217 6.693880
H 1.415446 10.117366 4.001391
H -5.632784 8.829254 5.711128
H 0.480815 10.781826 5.343866
H -2.591120 9.871495 2.070698
H -0.230194 6.257668 6.353664
H 1.199473 6.881998 7.195799
H -3.630214 7.862731 1.225577
H -1.872767 7.762114 1.114200
H -3.326893 5.678883 1.881208
H -3.373106 6.460238 3.451405
H -0.132524 4.311391 2.413926
H -1.592703 4.296212 1.482558
H 1.240494 7.980447 4.930799
H -2.862621 9.411272 5.267418
H -1.094129 8.108831 3.883903
N -2.392684 0.692069 -0.297252
H -3.412324 0.541130 -0.330881
H -1.998259 1.399227 -0.916746
H -2.205348 -0.550575 -2.453815
H -8.673233 0.171683 -0.642158

237
inactive_fGln123_TS2
H 5.426754 1.383429 0.621742
H 4.212221 1.610985 -0.266251
H -2.643196 -0.298343 -3.474686
H -2.681137 1.194097 -3.206091
N 1.398883 -3.893164 -2.154292
C 0.683921 -8.611231 -0.998826
C 1.195167 -7.252012 -0.521984
C 0.727282 -6.112069 -1.424869
C 1.470602 -4.808391 -1.163085
O 2.127910 -4.623993 -0.135601
H -0.411536 -8.626183 -1.040530
H 1.009341 -9.415193 -0.328562
H 1.058186 -8.839893 -2.004380
H 2.290136 -7.255903 -0.485312
H 0.861655 -7.059573 0.504325
H 0.872024 -6.382584 -2.479255
H -0.351565 -5.941796 -1.301961
H 0.638034 -3.932391 -2.818265
H 1.850816 -2.985528 -2.019913
C 8.882932 -3.299291 1.872839
C 7.394403 -2.958935 1.898404
C 7.125936 -1.574694 2.503080
C 5.647186 -1.211109 2.399885
O 4.831877 -1.828308 3.112666
O 5.318510 -0.309517 1.550588
H 9.055173 -4.300642 1.461532
H 9.315475 -3.268579 2.881361
H 9.430282 -2.581531 1.249943
H 7.003500 -2.987988 0.875079
H 6.836826 -3.707670 2.472031
H 7.409442 -1.584399 3.563152
H 7.733317 -0.820268 1.993842
N 0.118969 2.668123 -4.013920
N 3.431572 1.261969 -3.670360
N 4.669696 -1.225594 -4.152679
C -2.064950 3.680232 -4.359607
C -1.078169 2.919946 -3.480586
C -3.065136 4.582263 -3.612592
C -4.149808 3.796445 -2.867278
C -2.359407 5.581781 -2.690574
C 1.113621 1.831801 -3.380908
C 2.375345 1.839449 -4.256483
C 4.770567 1.218277 -4.223138
C 5.151762 -0.151398 -4.815204
C 5.772027 1.565555 -3.091009
C 7.228311 1.511742 -3.535683
C 5.169021 -2.572044 -4.356485
C 5.662785 -3.179984 -3.033584
C 6.797558 -2.388160 -2.431464
C 8.090337 -2.490498 -2.947017
C 6.582213 -1.487134 -1.385598
C 9.136748 -1.727155 -2.438682
C 7.612887 -0.707056 -0.874329
C 8.893081 -0.829931 -1.401323
O -1.389317 2.509428 -2.345135
O 2.345910 2.329856 -5.387379
O 5.928083 -0.224067 -5.768044
O 5.456578 2.848713 -2.586101
O 9.886147 -0.046642 -0.865240
H -2.611341 2.916193 -4.928578
H 0.748025 0.801079 -3.284492
H 4.378372 -3.207215 -4.775917
H 5.978601 -2.509240 -5.087959
H -1.503706 4.279541 -5.087090
H 1.340336 2.190876 -2.373072
H 5.979844 -4.212409 -3.230081
H 4.824111 -3.231551 -2.329681
H 3.975386 -1.098582 -3.414640
H -3.723932 3.231435 -2.033689
H -4.911345 4.477624 -2.469653
H -1.604552 6.163077 -3.233294
H -3.080203 6.285111 -2.257420
H -3.571261 5.160756 -4.398341
H 0.367823 2.970424 -4.950739
H 3.314775 1.014272 -2.682286
H 4.844440 1.954813 -5.026379
H 5.624825 0.821055 -2.296346
H 7.526546 0.502967 -3.834068
H 7.394574 2.189933 -4.379387
H 7.857692 1.827510 -2.697647
H 8.286445 -3.177472 -3.767481
H 10.137645 -1.825425 -2.856036
H 7.415582 -0.002788 -0.074244
H 5.590669 -1.380402 -0.952877
H 10.689929 -0.159775 -1.385288
H 5.427078 2.770092 -1.612813
H -1.856339 5.059272 -1.869542
H -4.648991 3.083181 -3.531761
N -7.538958 0.533483 -1.059167
N -5.375368 0.398978 -1.136231
C -8.900542 -0.548507 -3.794502
C -8.177405 0.767560 -3.481850
C -7.170455 0.627140 -2.385700
C -5.808440 0.534774 -2.435718
C -6.440825 0.398337 -0.326053
H -8.181796 -1.317987 -4.097655
H -9.611172 -0.401145 -4.615588
H -9.456975 -0.904343 -2.920986
H -7.657390 1.139630 -4.371741
H -8.911523 1.527231 -3.190279
H -8.615389 0.621359 -0.727228
H -5.088202 0.542435 -3.245715
H -6.398278 0.314301 0.748800
C -13.978176 -0.139021 -2.012446
C -13.524546 -0.174980 -0.549774
C -12.316212 0.713594 -0.239242
C -10.940997 0.158466 -0.652210
O -10.846199 -0.976060 -1.142021
O -9.965901 0.963685 -0.420158
H -13.168256 -0.477104 -2.666805
H -14.259378 0.878896 -2.312398
H -14.844598 -0.790960 -2.175245
H -14.362976 0.136659 0.089135
H -13.277163 -1.206377 -0.276709
H -12.429370 1.703976 -0.699607
H -12.255714 0.899693 0.840654
O -3.068842 0.533647 -3.799698
N -4.053639 -0.349350 2.716993
N -1.597280 -0.876546 4.071204
N 1.621752 -1.708602 5.661190
C -5.242092 -2.438879 2.500929
C -4.863270 -1.244514 3.356471
C -3.338235 0.708739 3.420601
C -2.396136 0.135321 4.508525
C -4.225662 1.850812 3.948373
C -3.375482 3.104387 4.180632
C -5.368025 2.155478 2.978069
C -0.686144 -1.536718 4.958152
C 0.784294 -1.138098 4.774123
C 3.068130 -1.502437 5.699200
O -5.267881 -1.082805 4.501216
O -2.358233 0.561695 5.654920
O 1.148938 -0.387915 3.869131
H 3.511226 -2.306210 6.290096
H 3.494316 -1.540288 4.692564
H -5.810101 -3.146025 3.106082
H 3.309918 -0.537727 6.159047
H -5.860309 -2.122950 1.652763
H -4.354959 -2.935054 2.089183
H -6.079324 1.327001 2.925255
H -5.916133 3.045720 3.305076
H -4.992848 2.352073 1.963102
H -2.936234 3.449965 3.233931
H -3.991919 3.918303 4.578914
H -2.563444 2.914359 4.886475
H -4.649124 1.527666 4.903331
H -2.668085 1.141279 2.661379
H -1.588204 -1.114749 3.082709
H -0.759927 -2.620394 4.818105
H -0.999881 -1.306502 5.980854
H 1.205028 -2.276256 6.383477
H -3.555729 -0.714642 1.903512
O 4.999377 2.074564 0.081816
N 0.629733 8.708602 5.317141
N -1.446540 8.986403 3.488146
N -0.887467 4.930718 1.933370
N -3.790797 9.365327 4.857120
C 0.132934 7.048172 7.019498
C -0.179764 8.372980 6.359901
C 0.487552 9.964495 4.616379
C -0.735147 10.112291 3.707964
C -2.657091 8.976119 2.698725
C -3.939259 9.154514 3.535667
C -2.764457 7.737279 1.799039
C -2.761605 6.371445 2.525437
C -1.363028 5.845726 2.801085
C -4.940102 9.676142 5.684911
O -1.097628 9.107882 6.721096
O -1.008618 11.211523 3.238100
O -5.036416 9.125702 2.980543
O -0.678261 6.255754 3.745123
H -0.415005 6.981025 7.959581
H -5.408361 10.612970 5.365015
H -4.603429 9.769837 6.718784
H 1.379891 10.129916 4.007029
H -5.688257 8.881237 5.615383
H 0.423983 10.779988 5.341534
H -2.624563 9.860454 2.053651
H -0.186613 6.235054 6.357653
H 1.205509 6.936893 7.205059
H -3.709872 7.831877 1.259727
H -1.958303 7.767398 1.057166
H -3.315817 5.647078 1.920232
H -3.284217 6.445055 3.485045
H 0.048849 4.573442 2.060666
H -1.425884 4.592430 1.151335
H 1.240951 7.998754 4.940832
H -2.864262 9.394020 5.266910
H -1.108902 8.100539 3.862309
N -2.424563 -4.067279 0.348574
C -3.809101 -3.832873 -1.689093
C -2.998586 -4.693248 -0.716541
C -3.545633 -2.327911 -1.675370
C -1.433947 -4.729217 1.183028
C -1.725816 -4.642262 2.676876
C -0.715714 -5.450144 3.508251
C 0.713955 -4.903052 3.403184
C -1.164028 -5.553394 4.969106
O -2.928721 -5.912731 -0.884220
S -1.836087 -1.859149 -2.111123
H -4.867760 -4.031128 -1.466494
H -1.411759 -5.779481 0.878253
H -0.449651 -4.300292 0.959726
H -3.611018 -4.241902 -2.683640
H -2.331900 -3.058267 0.302983
H -1.725802 -3.589600 2.997455
H -2.739359 -5.023531 2.857981

H -0.708498 -6.471132 3.097582
H -0.465612 -6.164730 5.553064
H -1.215720 -4.565180 5.443775
H -2.160233 -6.003397 5.048274
H 1.101078 -4.925911 2.379378
H 0.771540 -3.864025 3.753010
H 1.399214 -5.491577 4.024649
H -4.250078 -1.861110 -2.372638
H -3.791143 -1.925435 -0.678446
N 2.653018 -0.309511 1.472256
C 2.219313 -1.231186 0.388635
C 2.684149 -0.619956 -0.941415
O 2.778333 -1.378616 -1.927980
C 0.700624 -1.450589 0.389992
C -0.110803 -0.225899 -0.023695
C -1.603749 -0.410259 0.146829
N -2.448429 0.629184 -0.303353
O -2.047738 -1.160771 1.039031
O 2.883485 0.629420 -0.944628
H 2.715668 -2.191563 0.550080
H 0.470601 -2.269738 -0.296763
H 0.396935 -1.778879 1.390462
H 0.130000 0.631706 0.628465
H 0.111918 0.090679 -1.044953
H -2.006335 1.257577 -0.975649
H -2.832351 1.142615 0.486529
H 3.725829 -0.324019 1.564234
H 2.403687 0.646050 1.208577
H 2.236369 -0.526274 2.389028
H -4.386714 0.299176 -0.868263

237
inactive_fGln123_TS3
H 5.347576 1.296040 0.580379
H 4.160438 1.581883 -0.328723
H -2.876827 -0.474967 -4.101556
H -2.654877 0.958310 -3.691549
N -2.469124 -4.127460 0.361737
N 2.508705 -0.253798 1.384141
C 2.070467 -1.180779 0.307014
C -3.808841 -3.833076 -1.688937
C -2.906382 -4.687624 -0.789240
C -3.683029 -2.320393 -1.585455
C -1.434502 -4.731159 1.186400
C -1.767517 -4.678254 2.673362
C -0.766743 -5.473285 3.528841
C 0.689953 -5.027267 3.345484
C -1.172709 -5.443488 5.006165
C 2.565102 -0.586348 -1.020440
C 0.554359 -1.430145 0.285729
C -0.334750 -0.255586 -0.140978
C -1.821145 -0.676061 -0.232169
O -2.646141 -5.847242 -1.122300
O -2.260767 -1.316756 0.826292
O 2.672858 -1.351892 -2.003097
O 2.788817 0.657692 -1.030535
S -1.997776 -1.683112 -1.867138
H -4.851896 -4.087051 -1.456246
H -1.297742 -5.777730 0.899674
H -0.485720 -4.220078 0.987616
H -3.620274 -4.178216 -2.709240
H -2.541223 -3.112650 0.481655
H -1.819580 -3.632175 3.006152
H -2.772752 -5.096384 2.822550
H -0.828447 -6.519597 3.192795
H -0.501431 -6.063550 5.611492
H -1.135033 -4.421844 5.407414
H -2.195786 -5.809078 5.146317
H 1.355000 -5.629468 3.975763
H 1.036831 -5.122110 2.311993
H 0.837448 -3.978178 3.635740
H 0.388149 -2.270942 -0.392969
H 0.246578 -1.779727 1.278335
H -0.280546 0.547392 0.610872
H -0.003259 0.173309 -1.094777
H -4.370776 -1.854930 -2.294718
H -3.979333 -1.981778 -0.588539
H 2.565086 -2.139500 0.485150
H 3.577374 -0.319889 1.499681
H 2.304524 0.705606 1.095903
H 2.070850 -0.424908 2.302057
N 1.640225 -3.979936 -2.215732
C 0.684002 -8.610384 -0.998189
C 1.243725 -7.264897 -0.543623
C 0.808604 -6.140028 -1.480012
C 1.481343 -4.815287 -1.166338
O 1.862602 -4.526361 -0.030146
H -0.409199 -8.572224 -1.063953
H 0.951462 -9.417271 -0.304874
H 1.067731 -8.881173 -1.990039
H 2.338910 -7.305268 -0.499424
H 0.906636 -7.034132 0.473542
H 1.020221 -6.416842 -2.521319
H -0.279523 -5.998639 -1.409788
H 1.191316 -4.184413 -3.093759
H 2.004689 -3.032006 -2.072546
C 8.882501 -3.298280 1.874920
C 7.381190 -2.989531 1.835143
C 7.026567 -1.620615 2.431429
C 5.530280 -1.316418 2.298594
O 4.724505 -1.998647 2.958300
O 5.186086 -0.381910 1.488358
H 9.073432 -4.284659 1.437202
H 9.266408 -3.295681 2.902728
H 9.445718 -2.555232 1.298575
H 7.041837 -3.025409 0.794345
H 6.821564 -3.760499 2.375928
H 7.279786 -1.617799 3.500007
H 7.611711 -0.835335 1.943523
N 0.155119 2.721973 -4.046846
N 3.449627 1.272989 -3.733505
N 4.664545 -1.223468 -4.168721
C -2.065621 3.681138 -4.359353
C -1.105895 2.768647 -3.614387
C -3.124443 4.381284 -3.481059
C -4.241347 3.454842 -2.997258
C -2.481757 5.139136 -2.314537
C 1.129714 1.816876 -3.481272
C 2.413507 1.891305 -4.315835
C 4.796621 1.221678 -4.260352
C 5.172608 -0.157686 -4.830192
C 5.785280 1.571352 -3.116733
C 7.245418 1.530834 -3.550315
C 5.169420 -2.570939 -4.355264
C 5.649421 -3.171402 -3.024290
C 6.781674 -2.377437 -2.419473
C 8.079448 -2.498768 -2.917667
C 6.560004 -1.455319 -1.394321
C 9.125100 -1.733060 -2.411995
C 7.589713 -0.671517 -0.887153
C 8.875358 -0.813081 -1.396660
O -1.482067 2.064445 -2.656812
O 2.416011 2.454333 -5.412188
O 5.964227 -0.250962 -5.767749
O 5.455488 2.847199 -2.605410
O 9.865167 -0.025125 -0.864920
H -2.557178 3.075035 -5.130199
H 0.750904 0.787076 -3.496660
H 4.389192 -3.211681 -4.787483
H 5.987122 -2.493971 -5.074307
H -1.486808 4.465231 -4.862735
H 1.332383 2.065238 -2.435807
H 5.964897 -4.205735 -3.210409
H 4.804840 -3.216112 -2.326791
H 3.958525 -1.085731 -3.444885
H -3.874249 2.738848 -2.258215
H -5.047129 4.032678 -2.531605
H -1.699862 5.826165 -2.660575
H -3.233677 5.726767 -1.775962
H -3.587252 5.127282 -4.141961
H 0.458928 3.206768 -4.885557
H 3.306991 1.000453 -2.755950
H 4.886885 1.949876 -5.069519
H 5.636037 0.821317 -2.327921
H 7.552787 0.526874 -3.855746
H 7.412839 2.217840 -4.386473
H 7.866679 1.843681 -2.705939
H 8.281054 -3.203758 -3.721860
H 10.130668 -1.846877 -2.815044
H 7.389289 0.051643 -0.105773
H 5.565470 -1.335687 -0.969674
H 10.677697 -0.158396 -1.366824
H 5.413658 2.757276 -1.633928
H -2.027426 4.436105 -1.606253
H -4.671426 2.881974 -3.825428
N -7.456395 0.307877 -0.966029
N -5.254906 0.354734 -0.975472
C -8.900931 -0.548257 -3.794660
C -8.044816 0.667159 -3.405094
C -7.074721 0.465164 -2.278111
C -5.704786 0.482576 -2.273202
C -6.344454 0.249786 -0.216743
H -8.263614 -1.416672 -3.998127
H -9.457302 -0.317344 -4.710482
H -9.622221 -0.820599 -3.014555
H -7.455616 0.996454 -4.269220
H -8.709519 1.498955 -3.141296
H -5.014560 0.567546 -3.103272
H -6.339204 0.145593 0.857936
C -13.978046 -0.139066 -2.012444
C -13.549883 -0.753782 -0.676125
C -12.357681 -0.043107 -0.026464
C -10.970449 -0.375796 -0.620651
O -10.854860 -1.388708 -1.335907
O -10.037390 0.426092 -0.284064
H -13.164837 -0.209393 -2.742802
H -14.217703 0.922331 -1.867133
H -14.860147 -0.640704 -2.427127
H -14.403545 -0.724262 0.015689
H -13.285201 -1.806404 -0.823937
H -12.489216 1.045961 -0.045096
H -12.298943 -0.317033 1.035610
O -3.148602 0.423361 -4.336292
N -4.037334 -0.325056 2.574035
N -1.480591 -0.827428 3.721184
N 1.634026 -1.727390 5.490468
C -5.242758 -2.437864 2.501774
C -4.769464 -1.223162 3.286940
C -3.285626 0.739507 3.226786
C -2.251285 0.171592 4.229958
C -4.146213 1.860584 3.836664
C -3.303745 3.129275 3.999590
C -5.376184 2.146255 2.973174
C -0.592154 -1.556474 4.575998
C 0.858274 -1.057057 4.617662
C 3.066560 -1.501513 5.700551
O -5.048883 -1.072797 4.472657
O -2.123061 0.585153 5.374521
O 1.271866 -0.144070 3.902974
H 3.356279 -2.126809 6.546786
H 3.647333 -1.764255 4.809194
H -6.060143 -2.853934 3.092707
H 3.240435 -0.450053 5.947678
H -5.584421 -2.179596 1.494591
H -4.433569 -3.170764 2.410320
H -6.078567 1.309800 2.984903
H -5.903607 3.031255 3.348258
H -5.098899 2.339827 1.927383
H -2.980833 3.497751 3.014401
H -3.889903 3.924123 4.473244
H -2.414590 2.948717 4.609116
H -4.474662 1.524861 4.823726
H -2.687465 1.191183 2.420904
H -1.597578 -1.103877 2.743861
H -0.570669 -2.599695 4.251796
H -0.998194 -1.531147 5.593137
H 1.200937 -2.484467 5.997559
H -3.576933 -0.704952 1.734982
O 4.962048 2.007463 0.033924
C 0.131495 7.048801 7.021570
C -0.148256 8.400492 6.402536
O -1.053217 9.140847 6.783787
N 0.666036 8.750078 5.367959
C 0.525560 10.017626 4.687065
C -0.689116 10.178008 3.768825
O -0.950075 11.280766 3.299199
N -1.408285 9.057963 3.540607
C -2.600699 9.049796 2.723858
C -3.904313 9.202729 3.534424
O -4.989866 9.167618 2.956865
C -2.676916 7.820313 1.808187
C -2.696401 6.446262 2.518809
C -1.310006 5.915836 2.843536
N -0.814373 4.984811 2.004910
O -0.646488 6.340342 3.795977
N -3.781125 9.399780 4.859924
C -4.942476 9.676840 5.685452
H -0.412339 6.974100 7.963213
H -5.490899 10.541774 5.300844
H -4.580152 9.886134 6.694799
H 1.422560 10.197392 4.088647
H -5.621821 8.819308 5.708322

H 0.453162 10.819359 5.425865
H -2.560927 9.942653 2.090875
H -0.225921 6.268256 6.339973
H 1.198725 6.881794 7.197012
H -3.604660 7.918482 1.240275
H -1.846809 7.859342 1.093579
H -3.228590 5.729280 1.885050
H -3.256398 6.507999 3.458107
H 0.095656 4.590422 2.195457
H -1.358884 4.593945 1.253669
H 1.259151 8.037203 4.968300
H -2.860823 9.424795 5.282111
H -1.076209 8.168150 3.909303
H -3.942619 0.377415 -0.677377
N -2.691800 0.552812 -0.486209
H -2.619074 1.148202 0.342329
H -2.320261 1.095278 -1.275825
H -8.478746 0.267567 -0.649374

237
inactive_fGln123_TS4
H 5.434446 1.440408 0.604531
H 4.232472 1.588513 -0.319325
H -3.050260 -0.516395 -4.269687
H -2.761709 0.868245 -3.695928
H -2.279021 1.247966 -1.251730
H -2.414734 1.479304 0.380015
H -3.589009 0.612614 -0.411354
N -2.551328 -4.206878 0.404009
N 2.648288 -0.190733 1.442782
C 2.280414 -1.178895 0.398438
C -3.808673 -3.833091 -1.688994
C -2.883101 -4.698733 -0.817223
C -3.644852 -2.318887 -1.525256
C -1.434641 -4.731046 1.185452
C -1.678077 -4.636946 2.685534
C -0.582509 -5.343098 3.501754
C 0.817370 -4.761863 3.264607
C -0.937145 -5.349191 4.992021
C 2.691099 -0.585825 -0.958729
C 0.792336 -1.549200 0.409883
C -0.176922 -0.422324 0.017542
C -1.588619 -0.988207 -0.078086
O -2.498040 -5.794093 -1.230220
O -2.138659 -1.462294 0.925656
O 2.794092 -1.366675 -1.929473
O 2.853003 0.666399 -1.002478
S -1.931778 -1.709858 -1.738172
H -4.852014 -4.087600 -1.456403
H -1.297424 -5.777578 0.899161
H -0.519021 -4.196950 0.904667
H -3.621686 -4.142557 -2.720225
H -2.731931 -3.222031 0.581256
H -1.757693 -3.581139 2.982458
H -2.649691 -5.094449 2.913818
H -0.562652 -6.391160 3.166162
H -0.184017 -5.895689 5.572176
H -0.993701 -4.329420 5.393685
H -1.911482 -5.818634 5.168951
H 1.558110 -5.280083 3.885120
H 1.143750 -4.850223 2.223420
H 0.863368 -3.698582 3.532446
H 0.678444 -2.391629 -0.277295
H 0.531137 -1.927743 1.405356
H -0.187680 0.349873 0.795401
H 0.107454 0.048885 -0.929542
H -4.272539 -1.800799 -2.253543
H -3.987127 -1.970713 -0.546302
H 2.857100 -2.087018 0.594003
H 3.721375 -0.193273 1.561300
H 2.382933 0.743882 1.124627
H 2.221225 -0.366598 2.365049
N 1.761964 -3.990202 -2.223316
C 0.684497 -8.610748 -0.998507
C 1.300923 -7.282158 -0.568160
C 0.903172 -6.150079 -1.511243
C 1.598269 -4.840498 -1.185506
O 1.987102 -4.568945 -0.047987
H -0.408720 -8.538753 -1.032617
H 0.951612 -9.416909 -0.305569
H 1.030041 -8.900423 -1.998671
H 2.393873 -7.366143 -0.533882
H 0.984203 -7.025914 0.448669
H 1.122051 -6.430934 -2.550459
H -0.182738 -5.984474 -1.455135
H 1.318436 -4.180312 -3.107867
H 2.137706 -3.050450 -2.061935
C 8.882497 -3.298443 1.873297
C 7.387401 -2.975952 1.864702
C 7.078327 -1.601904 2.474092
C 5.599674 -1.240115 2.329976
O 4.754030 -1.924771 2.936728
O 5.306544 -0.253422 1.563401
H 9.073411 -4.284936 1.435992
H 9.286557 -3.293725 2.893806
H 9.438848 -2.557239 1.286925
H 7.025552 -3.001179 0.830653
H 6.825045 -3.739176 2.413846
H 7.324220 -1.620213 3.543898
H 7.696073 -0.833216 1.999907
N 0.185527 2.773938 -4.038360
N 3.468432 1.282322 -3.735412
N 4.676081 -1.220650 -4.163046
C -2.065415 3.680489 -4.360044
C -1.100465 2.727467 -3.677424
C -3.086864 4.350263 -3.414499
C -4.198521 3.413267 -2.931432
C -2.393972 5.043384 -2.235741
C 1.155570 1.850092 -3.493938
C 2.445992 1.927990 -4.314742
C 4.818502 1.223858 -4.256711
C 5.186087 -0.157576 -4.827168
C 5.805289 1.574702 -3.113322
C 7.266608 1.507924 -3.539124
C 5.168981 -2.571461 -4.356267
C 5.650072 -3.182724 -3.030776
C 6.780195 -2.392825 -2.417698
C 8.079896 -2.507543 -2.912589
C 6.554422 -1.481352 -1.383254
C 9.122863 -1.746087 -2.394736
C 7.581418 -0.702745 -0.863040
C 8.868909 -0.838213 -1.369033
O -1.484026 1.914508 -2.816442
O 2.466454 2.511272 -5.399478
O 5.970105 -0.254869 -5.770725
O 5.489584 2.863359 -2.625894
O 9.857106 -0.056750 -0.823965
H -2.596855 3.105917 -5.129057
H 0.766043 0.825686 -3.534367
H 4.384912 -3.205019 -4.790954
H 5.987107 -2.494180 -5.075637
H -1.486589 4.465063 -4.863817
H 1.355112 2.068962 -2.440214
H 5.968406 -4.214565 -3.227979
H 4.806318 -3.237643 -2.333341
H 3.990005 -1.078801 -3.421472
H -3.828640 2.691005 -2.200090
H -4.999513 3.987754 -2.452883
H -1.615997 5.738868 -2.572502
H -3.120931 5.608767 -1.641150
H -3.562170 5.131724 -4.023497
H 0.508204 3.326859 -4.824876
H 3.321776 1.005607 -2.759838
H 4.912958 1.950918 -5.066145
H 5.643244 0.837978 -2.314300
H 7.560687 0.495931 -3.831033
H 7.448256 2.182409 -4.382606
H 7.888375 1.821702 -2.694801
H 8.284600 -3.203125 -3.723614
H 10.129369 -1.854477 -2.795432
H 7.376674 0.007609 -0.070248
H 5.557191 -1.367211 -0.965715
H 10.671380 -0.183884 -1.324158
H 5.439412 2.794043 -1.652855
H -1.923611 4.299799 -1.580321
H -4.636714 2.844676 -3.757872
N -7.768400 0.263014 -0.856875
N -5.567785 0.223247 -0.549865
C -8.900803 -0.548360 -3.794581
C -8.013532 0.628040 -3.348897
C -7.208107 0.405026 -2.101702
C -5.848839 0.374581 -1.894824
C -6.759265 0.162504 0.028589
H -8.286139 -1.428558 -4.016417
H -9.449930 -0.276780 -4.703952
H -9.622154 -0.821257 -3.015183
H -7.307890 0.887644 -4.147722
H -8.651337 1.508752 -3.195909
H -8.803709 0.274716 -0.617985
H -5.065730 0.461470 -2.639727
H -6.933146 0.055777 1.090570
C -13.978344 -0.138949 -2.012466
C -13.738599 -0.832198 -0.671230
C -12.599257 -0.197376 0.124105
C -11.198059 -0.377567 -0.497868
O -11.010279 -1.329945 -1.281853
O -10.330470 0.471846 -0.119362
H -13.078989 -0.204826 -2.632426
H -14.217422 0.921895 -1.867688
H -14.808392 -0.600216 -2.562327
H -14.661605 -0.802625 -0.073396
H -13.495914 -1.885125 -0.849336
H -12.775307 0.874898 0.273012
H -12.552857 -0.641725 1.128123
N -2.577334 0.816975 -0.375137
O -3.351286 0.398672 -4.314376
N -4.097239 -0.302293 2.632922
N -1.470783 -0.672100 3.741081
N 1.623064 -1.673463 5.511949
C -5.242143 -2.438379 2.501406
C -4.577054 -1.359413 3.332122
C -3.362569 0.800440 3.227843
C -2.281929 0.308288 4.221714
C -4.257202 1.899130 3.839657
C -3.482604 3.218222 3.907268
C -5.543040 2.078451 3.029644
C -0.616184 -1.392567 4.640548
C 0.863772 -0.980480 4.639401
C 3.067522 -1.501048 5.699107
O -4.460345 -1.450241 4.551833
O -2.138557 0.799159 5.332913
O 1.312973 -0.104555 3.899437
H 3.356442 -2.126610 6.545409
H 3.624444 -1.788838 4.800995
H -6.059861 -2.854079 3.092141
H 3.284766 -0.454666 5.931455
H -5.621617 -2.068825 1.543797
H -4.516455 -3.235248 2.303266
H -6.195857 1.205918 3.114045
H -6.100587 2.949218 3.391659
H -5.334185 2.231640 1.962413
H -3.246118 3.564850 2.890977
H -4.085558 3.996530 4.388371
H -2.549093 3.112719 4.466057
H -4.511363 1.588154 4.857848
H -2.808674 1.251980 2.393853
H -1.659362 -1.046884 2.811325
H -0.665961 -2.457162 4.396165
H -1.009027 -1.266517 5.655396
H 1.164527 -2.400861 6.039660
H -4.193554 -0.317532 1.625428
O 4.992393 2.093192 0.030645
C 0.131896 7.049357 7.019831
C -0.146988 8.402114 6.404691
O -1.051105 9.142353 6.788452
N 0.667278 8.755011 5.370928
C 0.534121 10.028022 4.699896
C -0.677032 10.202343 3.779647
O -0.927843 11.310154 3.316860
N -1.402664 9.089541 3.541287
C -2.592338 9.096562 2.720167
C -3.897385 9.245532 3.526990
O -4.980615 9.226300 2.945336
C -2.672206 7.877154 1.791440
C -2.735427 6.497138 2.486604
C -1.369948 5.932860 2.839895
N -0.895104 4.965480 2.031315
O -0.704684 6.356939 3.791780
N -3.778901 9.415098 4.856617
C -4.941207 9.677090 5.684668
H -0.411763 6.971998 7.961821
H -5.498760 10.537736 5.303356
H -4.578924 9.886512 6.693628
H 1.433471 10.208647 4.105265
H -5.612812 8.813292 5.705094
H 0.463163 10.824145 5.445498
H -2.544611 9.995560 2.096760
H -0.225489 6.270300 6.336488
H 1.199402 6.882330 7.195654
H -3.586582 7.998055 1.205752
H -1.827477 7.907429 1.093863
H -3.267076 5.798975 1.832871

H -3.316510 6.561134 3.412966
H 0.000563 4.548100 2.241420
H -1.440432 4.580575 1.277008
H 1.263989 8.045277 4.971870
H -2.859185 9.430146 5.281247
H -1.080334 8.193178 3.905039

237
inactive_Val51_ES
H 5.542379 1.453361 0.798018
H 4.310934 1.652537 -0.074717
H -4.409603 -1.843107 -4.574631
H -5.785214 -1.331856 -4.117463
N 4.167304 3.951432 2.457057
N 2.271266 4.829664 0.675114
N -0.664399 1.630841 2.440260
N 0.310615 6.061187 2.146621
C 3.192312 2.596285 4.239063
C 3.326457 3.918835 3.518197
C 4.427466 5.176597 1.734720
C 3.278189 5.702348 0.876115
C 1.053939 5.179311 -0.014416
C 0.114419 6.061709 0.812955
C 0.288232 3.906521 -0.435192
C -0.618852 3.260251 0.620352
C 0.097252 2.415081 1.654671
C -0.596900 6.774098 3.015085
O 2.715142 4.925028 3.895613
O 3.307365 6.842287 0.422327
O -0.834430 6.632412 0.275743
O 1.331347 2.421067 1.771794
H 3.447604 2.750760 5.291612
H 5.283668 5.003330 1.077880
H -0.302667 6.584838 4.049188
H -0.570333 7.851952 2.820589
H -1.624382 6.430042 2.858416
H 4.681831 5.976587 2.434695
H 2.154987 2.260300 4.182201
H 3.839387 1.816646 3.824683
H -0.204936 0.975766 3.074192
H -1.647333 1.507696 2.239522
H 4.493370 3.095401 2.012178
H 2.303673 3.908624 1.101276
H 1.153736 5.655581 2.542685
H -0.344913 4.174963 -1.280499
H 1.019959 3.176404 -0.796730
H 1.309711 5.752840 -0.909473
H -1.349301 2.620550 0.114624
H -1.214148 4.016459 1.146166
N -2.304544 -4.027125 0.258053
C -3.809825 -3.832824 -1.688826
C -2.835743 -4.634930 -0.829208
C -3.658156 -2.325817 -1.804030
C -1.434959 -4.729845 1.184264
C -2.092697 -5.027801 2.532503
C -1.368318 -6.136009 3.313946
C 0.134438 -5.885898 3.488642
C -2.040276 -6.358487 4.670845
O -2.633072 -5.823094 -1.091408
S -2.312674 -1.901234 -2.976026
H -4.800368 -4.029355 -1.256767
H -1.152887 -5.670545 0.705016
H -0.517194 -4.148081 1.308558
H -3.817392 -4.304868 -2.675520
H -2.385943 -3.023468 0.390465
H -2.154002 -4.109410 3.136019
H -3.126215 -5.349751 2.349779
H -1.478487 -7.057521 2.723407
H -1.575657 -7.189544 5.214510
H -1.952548 -5.460055 5.296696
H -3.107275 -6.582205 4.555843
H 0.609822 -6.747893 3.971503
H 0.648803 -5.708376 2.538169
H 0.314700 -5.015314 4.130687
H -2.392431 -0.564220 -2.842835
H -4.589458 -1.903718 -2.179600
H -3.479287 -1.850049 -0.840183
N 1.718543 -3.897455 -1.884132
C 0.684466 -8.610544 -0.998852
C 1.230883 -7.290158 -0.459698
C 0.876029 -6.116629 -1.369812
C 1.492291 -4.803625 -0.910528
O 1.760842 -4.583157 0.272975
H -0.405619 -8.565620 -1.104255
H 0.927836 -9.443664 -0.329796
H 1.105388 -8.838994 -1.985747
H 2.320179 -7.349636 -0.347077
H 0.832988 -7.097998 0.542486
H 1.196447 -6.330465 -2.398259
H -0.215740 -5.990595 -1.405050
H 1.411886 -4.072823 -2.827725
H 2.122105 -2.978968 -1.674367
C 8.881896 -3.296149 1.870049
C 7.396762 -2.948318 1.971810
C 7.164557 -1.560703 2.584313
C 5.699533 -1.135298 2.495034
O 4.836617 -1.829795 3.067463
O 5.430493 -0.083043 1.814313
H 9.026727 -4.300505 1.455869
H 9.367686 -3.262223 2.853843
H 9.398387 -2.584187 1.215302
H 6.956205 -2.979944 0.969178
H 6.863102 -3.693441 2.571676
H 7.450000 -1.583377 3.644453
H 7.794930 -0.817098 2.087316
N 0.064906 2.589996 -3.968994
N 3.359923 1.209216 -3.585734
N 4.649558 -1.241203 -4.097054
C -2.065188 3.681482 -4.362213
C -1.218769 2.680353 -3.580822
C -3.183521 4.412867 -3.595632
C -4.365427 3.506772 -3.247316
C -2.668709 5.137923 -2.350911
C 0.987598 1.608755 -3.446699
C 2.299924 1.711634 -4.234323
C 4.712712 1.208819 -4.104915
C 5.126666 -0.139223 -4.722780
C 5.678352 1.557768 -2.945972
C 7.145890 1.517745 -3.353462
C 5.169259 -2.571005 -4.357151
C 5.672075 -3.228227 -3.061229
C 6.773488 -2.429641 -2.408057
C 8.080606 -2.484420 -2.892648
C 6.507658 -1.564279 -1.343649
C 9.093455 -1.710209 -2.334627
C 7.503472 -0.773691 -0.783284
C 8.800254 -0.850253 -1.278785
O -1.671497 1.967552 -2.673340
O 2.306280 2.191924 -5.368345
O 5.905839 -0.179799 -5.673321
O 5.328549 2.840778 -2.462007
O 9.755574 -0.059664 -0.691798
H -2.511112 3.120240 -5.195445
H 0.586387 0.593534 -3.569841
H 4.391971 -3.200717 -4.810064
H 5.979095 -2.461555 -5.082647
H -1.392904 4.423603 -4.810008
H 1.157992 1.757789 -2.376770
H 6.024648 -4.238787 -3.304990
H 4.830668 -3.341830 -2.367382
H 4.001262 -1.130338 -3.316564
H -4.082135 2.758789 -2.503895
H -5.190990 4.094972 -2.831334
H -1.773490 5.737430 -2.550311
H -3.432425 5.812431 -1.950748
H -3.545690 5.175145 -4.300854
H 0.399555 3.084178 -4.788613
H 3.244326 1.021901 -2.584517
H 4.793445 1.962543 -4.891831
H 5.514922 0.812438 -2.154473
H 7.460334 0.511765 -3.645476
H 7.325679 2.198720 -4.191624
H 7.753911 1.837358 -2.500975
H 8.315627 -3.141338 -3.727269
H 10.106704 -1.771106 -2.727982
H 7.270131 -0.099987 0.033582
H 5.498534 -1.497194 -0.944864
H 10.577405 -0.132184 -1.190599
H 5.380349 2.814146 -1.490201
H -2.429540 4.421345 -1.559025
H -4.744268 2.979050 -4.130768
N -7.467508 0.394540 -1.151989
N -5.249747 0.380285 -1.042391
C -8.900921 -0.548049 -3.794953
C -7.959839 0.649575 -3.604337
C -7.023839 0.504063 -2.444905
C -5.650229 0.486343 -2.357691
C -6.378359 0.329176 -0.356264
H -8.329720 -1.459380 -4.008863
H -9.573820 -0.367892 -4.641369
H -9.511324 -0.729218 -2.903049
H -7.358377 0.809062 -4.508092
H -8.561287 1.555992 -3.458303
H -8.481578 0.429316 -0.856673
H -4.930317 0.600703 -3.159150
H -6.432020 0.253770 0.720045
C -13.977073 -0.139527 -2.012262
C -13.615362 -0.323535 -0.537243
C -12.385748 0.477806 -0.108246
C -11.025851 -0.047049 -0.625834
O -10.971575 -1.187064 -1.128915
O -10.054435 0.756351 -0.457554
H -13.142776 -0.462375 -2.642674
H -14.189165 0.914278 -2.236746
H -14.862298 -0.727498 -2.285507
H -14.474033 -0.028728 0.084512
H -13.422661 -1.384722 -0.346348
H -12.480429 1.529178 -0.408771
H -12.311949 0.487838 0.987869
O -5.342374 -1.805625 -4.837271
O 5.048612 2.157195 0.331592
N 2.724718 -0.039310 1.687854
C 2.338828 -1.030757 0.650657
C 2.796831 -0.504696 -0.715209
O 2.903075 -1.332974 -1.647785
C 0.825616 -1.314029 0.671611
C -0.051760 -0.381415 -0.165947
C -1.517227 -0.447208 0.247012
N -2.345272 0.413558 -0.345286
O -1.909060 -1.277575 1.095379
O 2.979160 0.741156 -0.807397
H 2.873828 -1.953941 0.888424
H 0.676191 -2.346195 0.345086
H 0.495361 -1.266065 1.711312
H 0.290776 0.657746 -0.117980
H 0.003882 -0.660059 -1.225816
H -2.011751 0.983255 -1.121769
H -3.372516 0.341732 -0.261445
H 3.785346 -0.004889 1.771079
H 2.360126 0.899947 1.471287
H 2.349348 -0.289083 2.611885
N -1.441248 -1.455663 3.917262
N 1.807090 -2.095364 5.291866
C -5.240756 -2.438027 2.501120
C -3.297039 -0.111391 4.723657
C -2.020366 -0.908744 5.011802
C -3.758166 0.762043 5.894643
C -2.724356 1.862747 6.154867
C -5.133071 1.363690 5.597546
C -0.289614 -2.290143 4.091813
C 0.933771 -1.476118 4.496823
C 3.069078 -1.498687 5.694443
O -1.601517 -1.125535 6.146473
O 1.104914 -0.326659 4.064906
H 3.426475 -2.016982 6.586987
H 3.810163 -1.577309 4.891177
H 2.899538 -0.446437 5.935727
H -5.887778 0.586992 5.444323
H -5.457289 2.000509 6.428138
H -5.102108 1.978097 4.689945
H -2.604736 2.498515 5.267917
H -3.049605 2.504715 6.980700
H -1.747486 1.446071 6.414457
H -3.823776 0.128166 6.788062
H -3.130660 0.538063 3.857214
H -1.765244 -1.272313 2.965808
H -0.071479 -2.787105 3.141968
H -0.498626 -3.061043 4.841142
H 1.605659 -3.042474 5.571918
N -4.272090 -1.094316 4.274819
C -4.699646 -1.111407 2.974107
O -4.597728 -0.134007 2.247566
H -4.424289 -2.971144 1.998475
H -5.619241 -3.063592 3.315164
H -6.032783 -2.270788 1.768142
H -4.302230 -1.966403 4.783513

237
inactive_val51_TS2
H 5.342605 1.296381 0.821280
H 4.111786 1.711233 0.029552
H -2.642425 -0.447638 -3.561369

H -2.733952 1.047647 -3.386658
N 4.258821 3.996452 2.540720
N 2.457601 5.015768 0.732605
N -0.250905 2.049535 2.564292
N 0.393586 6.141655 2.169764
C 3.193943 2.597330 4.236510
C 3.364588 3.937666 3.557191
C 4.569914 5.253311 1.895470
C 3.445636 5.870953 1.062273
C 1.273472 5.401644 0.003815
C 0.276157 6.218316 0.828949
C 0.543544 4.144505 -0.506495
C -0.305979 3.388587 0.520721
C 0.463969 2.603603 1.565758
C -0.594203 6.774179 3.012420
O 2.753212 4.940555 3.946037
O 3.475385 7.053673 0.736824
O -0.657330 6.797684 0.273571
O 1.683263 2.407958 1.478250
H 3.632521 1.770891 3.671269
H 5.426664 5.089022 1.236167
H -1.594880 6.397085 2.776135
H -0.355789 6.546297 4.053222
H -0.603634 7.860355 2.870159
H 4.846159 6.001086 2.643079
H 3.674887 2.649605 5.219378
H 2.132222 2.399214 4.388048
H 0.186169 1.348834 3.166256
H -1.254835 2.134928 2.581537
H 4.538803 3.148020 2.045773
H 2.528981 4.042641 1.014055
H 1.228890 5.746670 2.593578
H -0.125616 4.452423 -1.311120
H 1.285961 3.467052 -0.939332
H 1.559671 6.023903 -0.848871
H -0.900671 2.650894 -0.032328
H -1.024328 4.055581 1.011843
N -1.374748 -1.083512 3.711981
N 1.740288 -1.952701 5.322219
C -5.240761 -2.438032 2.501123
C -3.299680 0.299970 4.194738
C -2.028045 -0.411595 4.679075
C -3.794307 1.387522 5.152357
C -2.788328 2.541975 5.186235
C -5.181821 1.882912 4.742474
C -0.321153 -1.984179 4.066392
C 0.943340 -1.260833 4.503617
C 3.069072 -1.498686 5.694455
O -1.711231 -0.468230 5.865988
O 1.227365 -0.137957 4.067400
H 3.377575 -2.035360 6.594677
H 3.783289 -1.675098 4.882387
H 3.024722 -0.429295 5.913746
H -5.915017 1.071062 4.749747
H -5.526216 2.663405 5.429978
H -5.164207 2.304547 3.730614
H -2.739367 3.044272 4.210283
H -3.093130 3.295888 5.919417
H -1.783480 2.201957 5.454733
H -3.846751 0.946561 6.155173
H -3.125702 0.745750 3.207782
H -1.682139 -1.071472 2.730108
H -0.075804 -2.577652 3.180813
H -0.651462 -2.674824 4.852219
H 1.463168 -2.889888 5.571157
N -2.449688 -4.086412 0.364650
C -3.809534 -3.832908 -1.688846
C -2.980863 -4.696097 -0.730233
C -3.547875 -2.332417 -1.663174
C -1.433599 -4.728836 1.183244
C -1.866829 -4.951502 2.630762
C -0.932916 -5.902518 3.397290
C 0.544506 -5.492610 3.355210
C -1.405994 -6.064835 4.844131
O -2.855304 -5.906133 -0.937711
S -1.822799 -1.891002 -2.037607
H -4.866214 -4.037286 -1.459968
H -1.211617 -5.697961 0.726630
H -0.520659 -4.125587 1.131076
H -3.617299 -4.237498 -2.686311
H -2.412030 -3.070136 0.379372
H -1.946906 -3.986559 3.153103
H -2.876749 -5.382017 2.622961
H -1.010785 -6.882772 2.903941
H -0.789493 -6.791118 5.387159
H -1.343484 -5.108040 5.380941
H -2.448259 -6.402087 4.888780
H 0.927955 -5.372166 2.336458
H 0.708201 -4.544181 3.882190
H 1.163348 -6.247469 3.854735
H -4.224579 -1.860131 -2.383081
H -3.811934 -1.923535 -0.675191
N 1.623420 -3.954762 -2.155431
C 0.683657 -8.610711 -0.998721
C 1.216803 -7.263143 -0.519522
C 0.793003 -6.121560 -1.441163
C 1.511795 -4.819094 -1.123456
O 1.988583 -4.592307 -0.008482
H -0.410740 -8.597434 -1.061888
H 0.975030 -9.419075 -0.318024
H 1.071403 -8.855384 -1.995394
H 2.310916 -7.292344 -0.458182
H 0.864184 -7.056077 0.496918
H 0.992394 -6.390092 -2.486997
H -0.291041 -5.953520 -1.369734
H 2.018021 -3.022545 -1.995114
H 1.008029 -4.055580 -2.949280
C 8.882305 -3.297379 1.866667
C 7.380936 -3.016405 1.929547
C 7.069510 -1.656573 2.570132
C 5.588146 -1.302871 2.445220
O 4.753201 -1.993541 3.061489
O 5.279338 -0.319685 1.683701
H 9.083717 -4.282136 1.429523
H 9.334961 -3.271400 2.866508
H 9.387033 -2.544997 1.248995
H 6.973266 -3.039438 0.912991
H 6.862857 -3.799557 2.493980
H 7.328274 -1.693923 3.636319
H 7.676714 -0.874672 2.103872
N 0.100320 2.635686 -4.002239
N 3.396517 1.233442 -3.622759
N 4.666032 -1.229220 -4.128424
C -2.064768 3.680198 -4.364681
C -1.132012 2.824073 -3.515385
C -3.180604 4.430907 -3.614831
C -4.299553 3.508961 -3.119120
C -2.639028 5.305415 -2.481106
C 1.043963 1.693853 -3.441507
C 2.345653 1.774203 -4.251851
C 4.757675 1.219257 -4.116766
C 5.178977 -0.132802 -4.729682
C 5.698498 1.541719 -2.926218
C 7.173033 1.541417 -3.308561
C 5.168513 -2.571471 -4.359473
C 5.640744 -3.216278 -3.046046
C 6.752310 -2.431242 -2.394089
C 8.059004 -2.506629 -2.877972
C 6.499955 -1.559505 -1.331941
C 9.083492 -1.746380 -2.322409
C 7.508101 -0.782446 -0.773876
C 8.803020 -0.879045 -1.269232
O -1.504799 2.282655 -2.458989
O 2.348057 2.277105 -5.377449
O 5.998720 -0.173027 -5.647390
O 5.325739 2.799638 -2.390762
O 9.772270 -0.101036 -0.684910
H -2.513311 2.995870 -5.098320
H 0.649323 0.670322 -3.499861
H 4.384061 -3.193779 -4.808808
H 5.990155 -2.489589 -5.075599
H -1.459026 4.398413 -4.931330
H 1.227488 1.906520 -2.385163
H 5.973741 -4.238605 -3.267809
H 4.787591 -3.298944 -2.362947
H 3.961948 -1.117991 -3.395752
H -3.946078 2.861230 -2.312274
H -5.139591 4.098795 -2.734326
H -1.805894 5.937954 -2.808016
H -3.421234 5.964581 -2.089684
H -3.622751 5.098265 -4.368842
H 0.383195 3.020092 -4.898005
H 3.267119 1.002561 -2.631524
H 4.864882 1.977731 -4.895988
H 5.530032 0.764916 -2.168226
H 7.506145 0.555915 -3.645358
H 7.356653 2.265788 -4.109028
H 7.762556 1.827101 -2.431364
H 8.283758 -3.169131 -3.711034
H 10.095892 -1.823499 -2.715549
H 7.282864 -0.103020 0.040248
H 5.494566 -1.474126 -0.927539
H 10.589228 -0.183783 -1.189984
H 5.326227 2.717429 -1.420675
H -2.285415 4.688999 -1.649184
H -4.676206 2.866230 -3.922520
N -7.632644 0.697227 -1.192454
N -5.498731 0.366730 -1.085805
C -8.900602 -0.548118 -3.795003
C -8.087993 0.743924 -3.655012
C -7.161745 0.665424 -2.489012
C -5.814870 0.454223 -2.423298
C -6.613113 0.514738 -0.364948
H -8.232178 -1.402848 -3.946776
H -9.574797 -0.484513 -4.657095
H -9.505132 -0.733057 -2.899943
H -7.494664 0.930127 -4.556763
H -8.765701 1.595407 -3.524121
H -8.686145 0.859608 -0.920433
H -5.043874 0.361534 -3.181851
H -6.650189 0.482239 0.710491
H -4.555610 0.190535 -0.723072
C -13.977686 -0.139373 -2.012191
C -13.585309 -0.031793 -0.537256
C -12.411747 0.914729 -0.280682
C -11.023613 0.390073 -0.694151
O -10.887041 -0.795356 -1.039838
O -10.089809 1.263104 -0.615664
H -13.133173 -0.515088 -2.598243
H -14.266396 0.840394 -2.414845
H -14.823221 -0.823792 -2.151633
H -14.454037 0.313014 0.041983
H -13.319939 -1.026491 -0.163403
H -12.570168 1.879352 -0.779878
H -12.343328 1.145425 0.790760
O -3.101140 0.339095 -3.938544
O 4.925767 2.089128 0.424277
N 2.585773 -0.316498 1.490983
C 2.210760 -1.234250 0.383651
C 2.723432 -0.618202 -0.921550
O 2.788739 -1.372411 -1.918313
C 0.703278 -1.506244 0.313382
C -0.158931 -0.310671 -0.072584
C -1.651070 -0.567033 0.095661
N -2.536871 0.460613 -0.280849
O -2.048112 -1.311915 1.033150
O 3.000620 0.612130 -0.907421
H 2.721003 -2.185087 0.561945
H 0.533351 -2.316967 -0.398476
H 0.378042 -1.885794 1.287020
H 0.054852 0.518647 0.609763
H 0.054676 0.044187 -1.083926
H -2.174093 1.139729 -0.945982
H -3.008675 0.850227 0.529375
H 3.645471 -0.318287 1.614572
H 2.301808 0.655522 1.296441
H 2.175610 -0.570403 2.394421
N -4.243891 -0.787250 3.964408
C -4.783916 -1.020200 2.736481
O -4.850466 -0.140582 1.882286
H -4.440210 -2.954172 1.954423
H -5.444505 -2.982976 3.427412
H -6.132257 -2.441599 1.870296
H -4.172129 -1.577168 4.589776

237
inactive_val51_TS3
H 5.240661 1.163526 0.805141
H 4.040432 1.659184 0.015107
H -2.680405 -0.476793 -4.081010
H -2.621303 0.970766 -3.684164
N -2.436969 -4.086149 0.352894
N 2.428091 -0.378862 1.369119
N -2.778146 0.562759 -0.351913
C 2.006233 -1.243222 0.238616
C -3.809524 -3.832913 -1.688843
C -2.911334 -4.669614 -0.772457
C -3.784680 -2.323112 -1.521463
C -1.434586 -4.729764 1.183223
C -1.894133 -4.947177 2.623506
C -0.997055 -5.928172 3.396459
C 0.493891 -5.570689 3.358121

C -1.481236 -6.069677 4.841694
C 2.566910 -0.621829 -1.043927
C 0.491282 -1.476656 0.148537
C -0.407273 -0.268674 -0.125909
C -1.905170 -0.672176 -0.150320
O -2.681986 -5.847283 -1.069950
O -2.303974 -1.332500 0.908705
O 2.603990 -1.350204 -2.063898
O 2.923952 0.585005 -0.990013
S -2.129449 -1.623881 -1.817912
H -4.835491 -4.194284 -1.536902
H -1.207738 -5.697865 0.727578
H -0.515253 -4.135926 1.144317
H -3.534437 -4.116945 -2.709468
H -2.501011 -3.068497 0.478722
H -1.954289 -3.982934 3.149173
H -2.915431 -5.349473 2.601896
H -1.107571 -6.906173 2.904776
H -0.893297 -6.816378 5.388572
H -1.386560 -5.114692 5.376662
H -2.534963 -6.369573 4.882764
H 1.085091 -6.350018 3.853374
H 0.879869 -5.455472 2.339874
H 0.689999 -4.631669 3.889700
H 0.339332 -2.226846 -0.631899
H 0.160724 -1.952598 1.074841
H -0.308486 0.449566 0.694145
H -0.124454 0.241915 -1.055358
H -2.361819 1.194838 -1.046646
H -2.836650 1.037037 0.551346
H -3.971301 0.418020 -0.707734
H -4.499868 -1.869076 -2.209978
H -4.083622 -2.023044 -0.511124
H 2.471157 -2.222191 0.389868
H 3.485903 -0.449327 1.501671
H 2.210959 0.613757 1.191329
H 1.995053 -0.613408 2.266575
N -7.448179 0.651384 -1.268166
N -5.259156 0.474939 -1.168319
C -8.900610 -0.548113 -3.795006
C -7.945592 0.651446 -3.728271
C -7.003697 0.592116 -2.568316
C -5.641079 0.478974 -2.495868
C -6.381193 0.579990 -0.462620
H -8.335479 -1.479367 -3.918072
H -9.579058 -0.445626 -4.650220
H -9.504889 -0.634547 -2.885231
H -7.349312 0.719149 -4.645825
H -8.534765 1.574544 -3.658980
H -8.467967 0.763013 -0.988187
H -4.914570 0.416069 -3.296069
H -6.424790 0.586339 0.613883
C -13.977684 -0.139374 -2.012191
C -13.566526 0.022052 -0.547063
C -12.353010 0.931270 -0.344185
C -10.984036 0.334558 -0.743250
O -10.913083 -0.871403 -1.049612
O -10.018088 1.163753 -0.698864
H -13.151638 -0.572567 -2.584563
H -14.233268 0.831089 -2.458058
H -14.849741 -0.797364 -2.113678
H -14.416771 0.426310 0.022229
H -13.335782 -0.964358 -0.130459
H -12.479432 1.877039 -0.887336
H -12.267958 1.209174 0.714870
N 4.258991 3.959635 2.512999
N 2.456909 5.012136 0.726511
N -0.209773 2.083549 2.537954
N 0.389790 6.134713 2.168518
C 3.192963 2.595451 4.236385
C 3.375847 3.925438 3.540891
C 4.587314 5.209133 1.861667
C 3.459770 5.852330 1.052358
C 1.278331 5.413965 -0.002809
C 0.274984 6.219058 0.827755
C 0.552691 4.167639 -0.543283
C -0.297828 3.389320 0.467130
C 0.479955 2.600388 1.504544
C -0.594190 6.772268 3.014251
O 2.786498 4.942291 3.926374
O 3.499648 7.037367 0.738310
O -0.658041 6.798807 0.272618
O 1.688641 2.366650 1.372584
H 3.729642 2.632573 5.190710
H 5.426256 5.025115 1.184736
H -0.364702 6.528782 4.053484
H -0.587420 7.860065 2.884788
H -1.597938 6.411604 2.767307
H 4.896442 5.950188 2.603077
H 2.134583 2.438195 4.448226
H 3.566687 1.750400 3.652547
H 0.235145 1.389186 3.142030
H -1.199670 2.243431 2.638408
H 4.521276 3.101733 2.024202
H 2.530397 4.033263 0.985321
H 1.231223 5.748756 2.588413
H -0.112570 4.491826 -1.344501
H 1.298649 3.500112 -0.985062
H 1.572568 6.051743 -0.841307
H -0.871780 2.649213 -0.105273
H -1.030721 4.041038 0.957061
N -1.290591 -1.140748 3.529361
N 1.771779 -1.971655 5.237747
C -5.240558 -2.439959 2.500157
C -3.199304 0.208943 4.191074
C -1.892867 -0.500848 4.553421
C -3.627450 1.257969 5.222204
C -2.640101 2.428615 5.209562
C -5.051781 1.742867 4.949424
C -0.211184 -2.024936 3.856459
C 1.001979 -1.277949 4.394966
C 3.067945 -1.502553 5.694315
O -1.491450 -0.588150 5.713938
O 1.273608 -0.132612 4.010551
H 3.835114 -1.706151 4.939035
H 3.005551 -0.426677 5.870845
H 3.309784 -2.006135 6.633413
H -5.768809 0.918243 4.994683
H -5.345410 2.496102 5.689322
H -5.129901 2.192165 3.952853
H -2.691726 2.967689 4.252907
H -2.887276 3.150800 5.994611
H -1.609509 2.098195 5.374256
H -3.587327 0.785074 6.210739
H -3.104061 0.689657 3.209604
H -1.671057 -1.134457 2.562970
H 0.091746 -2.559668 2.951620
H -0.535682 -2.773145 4.590651
H 1.511113 -2.925414 5.436196
N 1.628922 -3.988433 -2.242095
C 0.684684 -8.611670 -1.001701
C 1.232942 -7.256548 -0.566412
C 0.784907 -6.143144 -1.509368
C 1.435979 -4.809328 -1.187913
O 1.771227 -4.507828 -0.039939
H -0.410719 -8.593493 -1.039558
H 0.987869 -9.405981 -0.309879
H 1.047561 -8.881771 -2.001204
H 2.328583 -7.286875 -0.527880
H 0.899795 -7.020377 0.449767
H 1.009762 -6.420216 -2.548129
H -0.306092 -6.018530 -1.448740
H 1.222047 -4.216784 -3.135241
H 1.977266 -3.031474 -2.108738
C 8.883325 -3.298282 1.867578
C 7.368028 -3.069718 1.868104
C 6.971414 -1.729924 2.507254
C 5.473176 -1.441376 2.363576
O 4.664919 -2.141874 3.001694
O 5.129958 -0.493150 1.570825
H 9.135915 -4.268764 1.425374
H 9.290030 -3.273067 2.886791
H 9.388070 -2.519297 1.283863
H 7.006106 -3.097294 0.834286
H 6.857882 -3.878863 2.402868
H 7.212571 -1.760061 3.577755
H 7.548808 -0.917537 2.055654
N 0.125211 2.688468 -3.998095
N 3.413084 1.244993 -3.665071
N 4.664539 -1.225714 -4.147929
C -2.064787 3.680173 -4.362785
C -1.130214 2.797554 -3.548406
C -3.164782 4.426798 -3.584329
C -4.302058 3.510496 -3.127676
C -2.606174 5.240522 -2.414932
C 1.073284 1.734308 -3.468303
C 2.375849 1.831519 -4.276208
C 4.780037 1.225185 -4.139000
C 5.203977 -0.133252 -4.736812
C 5.705715 1.541594 -2.933118
C 7.183183 1.570450 -3.302090
C 5.168496 -2.571454 -4.359554
C 5.624726 -3.205010 -3.035233
C 6.738823 -2.422071 -2.385486
C 8.047164 -2.515052 -2.862124
C 6.487897 -1.534847 -1.336226
C 9.075116 -1.755572 -2.312575
C 7.500163 -0.758005 -0.785426
C 8.796466 -0.870822 -1.273283
O -1.517174 2.153839 -2.553356
O 2.388214 2.380401 -5.379424
O 6.049646 -0.185133 -5.629415
O 5.310492 2.782040 -2.374068
O 9.768003 -0.091329 -0.696091
H -2.528420 3.016847 -5.106101
H 0.679789 0.712517 -3.552143
H 4.388570 -3.196294 -4.813802
H 5.998513 -2.496769 -5.066502
H -1.458753 4.404910 -4.920622
H 1.260307 1.915505 -2.406586
H 5.949331 -4.233239 -3.241971
H 4.765637 -3.271992 -2.357408
H 3.925844 -1.109327 -3.451519
H -3.964061 2.812672 -2.357735
H -5.125058 4.098063 -2.705432
H -1.755144 5.865136 -2.709544
H -3.372788 5.902467 -1.999063
H -3.588277 5.134411 -4.311553
H 0.421017 3.137654 -4.858645
H 3.268740 0.990930 -2.681637
H 4.902225 1.980502 -4.918952
H 5.541141 0.747899 -2.192426
H 7.531754 0.598555 -3.661817
H 7.363196 2.318188 -4.081691
H 7.761899 1.841580 -2.413254
H 8.270514 -3.190782 -3.684881
H 10.088616 -1.846493 -2.699620
H 7.277888 -0.063503 0.016466
H 5.484066 -1.436326 -0.930509
H 10.588330 -0.190193 -1.192762
H 5.294202 2.673032 -1.406925
H -2.276379 4.579917 -1.606708
H -4.701756 2.918743 -3.958041
O -2.943014 0.398725 -4.401287
O 4.867554 1.987037 0.424057
N -4.147670 -0.880459 3.996525
C -4.795591 -1.031494 2.803889
O -4.953823 -0.088912 2.040813
H -4.434061 -2.924236 1.935644
H -5.439702 -3.026469 3.402499
H -6.133101 -2.418568 1.871842
H -3.984064 -1.717481 4.537707

237
inactive_val51_TS3
H 5.240661 1.163526 0.805141
H 4.040432 1.659184 0.015107
H -2.680405 -0.476793 -4.081010
H -2.621303 0.970766 -3.684164
N -2.436969 -4.086149 0.352894
N 2.428091 -0.378862 1.369119
N -2.778146 0.562759 -0.351913
C 2.006233 -1.243222 0.238616
C -3.809524 -3.832913 -1.688843
C -2.911334 -4.669614 -0.772457
C -3.784680 -2.323112 -1.521463
C -1.434586 -4.729764 1.183223
C -1.894133 -4.947177 2.623506
C -0.997055 -5.928172 3.396459
C 0.493891 -5.570689 3.358121
C -1.481236 -6.069677 4.841694
C 2.566910 -0.621829 -1.043927
C 0.491282 -1.476656 0.148537
C -0.407273 -0.268674 -0.125909
C -1.905170 -0.672176 -0.150320
O -2.681986 -5.847283 -1.069950
O -2.303974 -1.332500 0.908705
O 2.603990 -1.350204 -2.063898
O 2.923952 0.585005 -0.990013
S -2.129449 -1.623881 -1.817912
H -4.835491 -4.194284 -1.536902
H -1.207738 -5.697865 0.727578

H -0.515253 -4.135926 1.144317
H -3.534437 -4.116945 -2.709468
H -2.501011 -3.068497 0.478722
H -1.954289 -3.982934 3.149173
H -2.915431 -5.349473 2.601896
H -1.107571 -6.906173 2.904776
H -0.893297 -6.816378 5.388572
H -1.386560 -5.114692 5.376662
H -2.534963 -6.369573 4.882764
H 1.085091 -6.350018 3.853374
H 0.879869 -5.455472 2.339874
H 0.689999 -4.631669 3.889700
H 0.339332 -2.226846 -0.631899
H 0.160724 -1.952598 1.074841
H -0.308486 0.449566 0.694145
H -0.124454 0.241915 -1.055358
H -2.361819 1.194838 -1.046646
H -2.836650 1.037037 0.551346
H -3.971301 0.418020 -0.707734
H -4.499868 -1.869076 -2.209978
H -4.083622 -2.023044 -0.511124
H 2.471157 -2.222191 0.389868
H 3.485903 -0.449327 1.501671
H 2.210959 0.613757 1.191329
H 1.995053 -0.613408 2.266575
N -7.448179 0.651384 -1.268166
N -5.259156 0.474939 -1.168319
C -8.900610 -0.548113 -3.795006
C -7.945592 0.651446 -3.728271
C -7.003697 0.592116 -2.568316
C -5.641079 0.478974 -2.495868
C -6.381193 0.579990 -0.462620
H -8.335479 -1.479367 -3.918072
H -9.579058 -0.445626 -4.650220
H -9.504889 -0.634547 -2.885231
H -7.349312 0.719149 -4.645825
H -8.534765 1.574544 -3.658980
H -8.467967 0.763013 -0.988187
H -4.914570 0.416069 -3.296069
H -6.424790 0.586339 0.613883
C -13.977684 -0.139374 -2.012191
C -13.566526 0.022052 -0.547063
C -12.353010 0.931270 -0.344185
C -10.984036 0.334558 -0.743250
O -10.913083 -0.871403 -1.049612
O -10.018088 1.163753 -0.698864
H -13.151638 -0.572567 -2.584563
H -14.233268 0.831089 -2.458058
H -14.849741 -0.797364 -2.113678
H -14.416771 0.426310 0.022229
H -13.335782 -0.964358 -0.130459
H -12.479432 1.877039 -0.887336
H -12.267958 1.209174 0.714870
N 4.258991 3.959635 2.512999
N 2.456909 5.012136 0.726511
N -0.209773 2.083549 2.537954
N 0.389790 6.134713 2.168518
C 3.192963 2.595451 4.236385
C 3.375847 3.925438 3.540891
C 4.587314 5.209133 1.861667
C 3.459770 5.852330 1.052358
C 1.278331 5.413965 -0.002809
C 0.274984 6.219058 0.827755
C 0.552691 4.167639 -0.543283
C -0.297828 3.389320 0.467130
C 0.479955 2.600388 1.504544
C -0.594190 6.772268 3.014251
O 2.786498 4.942291 3.926374
O 3.499648 7.037367 0.738310
O -0.658041 6.798807 0.272618
O 1.688641 2.366650 1.372584
H 3.729642 2.632573 5.190710
H 5.426256 5.025115 1.184736
H -0.364702 6.528782 4.053484
H -0.587420 7.860065 2.884788
H -1.597938 6.411604 2.767307
H 4.896442 5.950188 2.603077
H 2.134583 2.438195 4.448226
H 3.566687 1.750400 3.652547
H 0.235145 1.389186 3.142030
H -1.199670 2.243431 2.638408
H 4.521276 3.101733 2.024202
H 2.530397 4.033263 0.985321
H 1.231223 5.748756 2.588413
H -0.112570 4.491826 -1.344501
H 1.298649 3.500112 -0.985062
H 1.572568 6.051743 -0.841307
H -0.871780 2.649213 -0.105273
H -1.030721 4.041038 0.957061
N -1.290591 -1.140748 3.529361
N 1.771779 -1.971655 5.237747
C -5.240558 -2.439959 2.500157
C -3.199304 0.208943 4.191074
C -1.892867 -0.500848 4.553421
C -3.627450 1.257969 5.222204
C -2.640101 2.428615 5.209562
C -5.051781 1.742867 4.949424
C -0.211184 -2.024936 3.856459
C 1.001979 -1.277949 4.394966
C 3.067945 -1.502553 5.694315
O -1.491450 -0.588150 5.713938
O 1.273608 -0.132612 4.010551
H 3.835114 -1.706151 4.939035
H 3.005551 -0.426677 5.870845
H 3.309784 -2.006135 6.633413
H -5.768809 0.918243 4.994683
H -5.345410 2.496102 5.689322
H -5.129901 2.192165 3.952853
H -2.691726 2.967689 4.252907
H -2.887276 3.150800 5.994611
H -1.609509 2.098195 5.374256
H -3.587327 0.785074 6.210739
H -3.104061 0.689657 3.209604
H -1.671057 -1.134457 2.562970
H 0.091746 -2.559668 2.951620
H -0.535682 -2.773145 4.590651
H 1.511113 -2.925414 5.436196
N 1.628922 -3.988433 -2.242095
C 0.684684 -8.611670 -1.001701
C 1.232942 -7.256548 -0.566412
C 0.784907 -6.143144 -1.509368
C 1.435979 -4.809328 -1.187913
O 1.771227 -4.507828 -0.039939
H -0.410719 -8.593493 -1.039558
H 0.987869 -9.405981 -0.309879
H 1.047561 -8.881771 -2.001204
H 2.328583 -7.286875 -0.527880
H 0.899795 -7.020377 0.449767
H 1.009762 -6.420216 -2.548129
H -0.306092 -6.018530 -1.448740
H 1.222047 -4.216784 -3.135241
H 1.977266 -3.031474 -2.108738
C 8.883325 -3.298282 1.867578
C 7.368028 -3.069718 1.868104
C 6.971414 -1.729924 2.507254
C 5.473176 -1.441376 2.363576
O 4.664919 -2.141874 3.001694
O 5.129958 -0.493150 1.570825
H 9.135915 -4.268764 1.425374
H 9.290030 -3.273067 2.886791
H 9.388070 -2.519297 1.283863
H 7.006106 -3.097294 0.834286
H 6.857882 -3.878863 2.402868
H 7.212571 -1.760061 3.577755
H 7.548808 -0.917537 2.055654
N 0.125211 2.688468 -3.998095
N 3.413084 1.244993 -3.665071
N 4.664539 -1.225714 -4.147929
C -2.064787 3.680173 -4.362785
C -1.130214 2.797554 -3.548406
C -3.164782 4.426798 -3.584329
C -4.302058 3.510496 -3.127676
C -2.606174 5.240522 -2.414932
C 1.073284 1.734308 -3.468303
C 2.375849 1.831519 -4.276208
C 4.780037 1.225185 -4.139000
C 5.203977 -0.133252 -4.736812
C 5.705715 1.541594 -2.933118
C 7.183183 1.570450 -3.302090
C 5.168496 -2.571454 -4.359554
C 5.624726 -3.205010 -3.035233
C 6.738823 -2.422071 -2.385486
C 8.047164 -2.515052 -2.862124
C 6.487897 -1.534847 -1.336226
C 9.075116 -1.755572 -2.312575
C 7.500163 -0.758005 -0.785426
C 8.796466 -0.870822 -1.273283
O -1.517174 2.153839 -2.553356
O 2.388214 2.380401 -5.379424
O 6.049646 -0.185133 -5.629415
O 5.310492 2.782040 -2.374068
O 9.768003 -0.091329 -0.696091
H -2.528420 3.016847 -5.106101
H 0.679789 0.712517 -3.552143
H 4.388570 -3.196294 -4.813802
H 5.998513 -2.496769 -5.066502
H -1.458753 4.404910 -4.920622
H 1.260307 1.915505 -2.406586
H 5.949331 -4.233239 -3.241971
H 4.765637 -3.271992 -2.357408
H 3.925844 -1.109327 -3.451519
H -3.964061 2.812672 -2.357735
H -5.125058 4.098063 -2.705432
H -1.755144 5.865136 -2.709544
H -3.372788 5.902467 -1.999063
H -3.588277 5.134411 -4.311553
H 0.421017 3.137654 -4.858645
H 3.268740 0.990930 -2.681637
H 4.902225 1.980502 -4.918952
H 5.541141 0.747899 -2.192426
H 7.531754 0.598555 -3.661817
H 7.363196 2.318188 -4.081691
H 7.761899 1.841580 -2.413254
H 8.270514 -3.190782 -3.684881
H 10.088616 -1.846493 -2.699620
H 7.277888 -0.063503 0.016466
H 5.484066 -1.436326 -0.930509
H 10.588330 -0.190193 -1.192762
H 5.294202 2.673032 -1.406925
H -2.276379 4.579917 -1.606708
H -4.701756 2.918743 -3.958041
O -2.943014 0.398725 -4.401287
O 4.867554 1.987037 0.424057
N -4.147670 -0.880459 3.996525
C -4.795591 -1.031494 2.803889
O -4.953823 -0.088912 2.040813
H -4.434061 -2.924236 1.935644
H -5.439702 -3.026469 3.402499
H -6.133101 -2.418568 1.871842
H -3.984064 -1.717481 4.537707
H -5.173369 -3.201647 -0.131003

References

(1) Czapinska, H.; Bochtler, M. The Nϵ-Rule for Serine, but Not Cysteine Catalytic Triads. *Angew Chem Int Ed* **2022**, *61* (42).

(2) Arafet, K.; Świderek, K.; Moliner, V. Computational Study of the Michaelis Complex Formation and the Effect on the Reaction Mechanism of Cruzain Cysteine Protease. *ACS Omega* **2018**, *3* (12), 18613–18622.

(3) Roux, B.; Walsh, C. T. P-Aminobenzoate Synthesis in Escherichia Coli: Mutational Analysis of Three Conserved Amino Acid Residues of the Amidotransferase PabA. *Biochemistry* **1993**, *32* (14), 3763–3768.

(4) Humphrey, W.; Dalke, A.; Schulten, K. VMD: Visual Molecular Dynamics. *Journal of Molecular Graphics* **1996**, *14* (1), 33–38.

(5) Chen, S.-L.; Fang, W.-H.; Himo, F. Technical Aspects of Quantum Chemical Modeling of Enzymatic Reactions: The Case of Phosphotriesterase. *Theor Chem Account* **2008**, *120* (4–6), 515–522.

(6) Kneuttinger, A. C.; Rajendran, C.; Simeth, N. A.; Bruckmann, A.; König, B.; Sterner, R. Significance of the Protein Interface Configuration for Allostery in Imidazole Glycerol Phosphate Synthase. *Biochemistry* **2020**, *59* (29), 2729–2742.

(7) Lisi, G. P.; Currier, A. A.; Loria, J. P. Glutamine Hydrolysis by Imidazole Glycerol Phosphate Synthase Displays Temperature Dependent Allosteric Activation. *Front. Mol. Biosci.* **2018**, *5*, 4.

(8) Calvó-Tusell, C.; Maria-Solano, M. A.; Osuna, S.; Feixas, F. Time Evolution of the Millisecond Allosteric Activation of Imidazole Glycerol Phosphate Synthase. *J. Am. Chem. Soc.* **2022**, *144* (16), 7146–7159.

(9) Rishavy, M. A.; Cleland, W. W.; Lusty, C. J. ^{15}N Isotope Effects in Glutamine Hydrolysis Catalyzed by Carbamyl Phosphate Synthetase: Evidence for a Tetrahedral Intermediate in the Mechanism. *Biochemistry* **2000**, *39* (24), 7309–7315.

(10) O'Leary, M. H.; Urberg, M.; Young, A. P. Nitrogen Isotope Effects on the Papain-Catalyzed Hydrolysis of N-Benzoyl-L-Argininamide. *Biochemistry* **1974**, *13* (10), 2077–2081.

(11) Glendening, E. D.; Landis, C. R.; Weinhold, F. *NBO 7.0* : New Vistas in Localized and Delocalized Chemical Bonding Theory. *J Comput Chem* **2019**, jcc.25873.

(12) Epifanovsky, E.; Gilbert, A. T. B.; Feng, X. Software for the Frontiers of Quantum Chemistry: An Overview of Developments in the Q-Chem 5 Package. *The Journal of Chemical* **2021**.

(13) Horn, P. R.; Mao, Y.; Head-Gordon, M. Probing Non-Covalent Interactions with a Second Generation Energy Decomposition Analysis Using Absolutely Localized Molecular Orbitals. *Phys. Chem. Chem. Phys.* **2016**, *18* (33), 23067–23079.

(14) Strohmeier, M.; Raschle, T.; Mazurkiewicz, J.; Rippe, K.; Sinning, I.; Fitzpatrick, T. B.; Tews, I. Structure of a Bacterial Pyridoxal 5′-Phosphate Synthase Complex. *Proc. Natl. Acad. Sci. U.S.A.* **2006**, *103* (51), 19284–19289.

(15) Thoden, J. B.; Huang, X.; Raushel, F. M.; Holden, H. M. The Small Subunit of Carbamoyl Phosphate Synthetase: Snapshots along the Reaction Pathway. *Biochemistry* **1999**, *38* (49), 16158–16166.
